# Informing the development of the SUCCEED reporting guideline for studies on the scaling of health interventions: A systematic review

**DOI:** 10.1097/MD.0000000000037079

**Published:** 2024-02-16

**Authors:** Amédé Gogovor, Hervé Tchala Vignon Zomahoun, Ali Ben Charif, Giraud Ekanmian, David Moher, Robert K. D. McLean, Andrew Milat, Luke Wolfenden, Karina Prévost, Emmanuelle Aubin, Paula Rochon, Nathalie Rheault, France Légaré

**Affiliations:** a VITAM – Centre de recherche en santé durable, Centre intégré universitaire de santé et services sociaux de la Capitale-Nationale, Quebec City, QC; b Department of Family Medicine and Emergency Medicine, Université Laval, Quebec City, QC; c Department of Social and Preventive Medicine, Université Laval, Quebec City, QC; d CubecXpert, Quebec City, QC; e Ottawa Methods Centre, Ottawa Hospital Research Institute, The Ottawa Hospital, Ottawa, ON; f School of Epidemiology and Public Health, University of Ottawa, Ottawa, ON; g International Development Research Centre, Ottawa, ON; h Integrated Knowledge Translation Research Network, Ottawa Hospital Research Institute, Ottawa, ON; i School of Public Health, University of Sydney, Camperdown, NSW; j School of Medicine and Public Health, The University of Newcastle, Newcastle, NSW; k The National Centre of Implementation Science, The University of Newcastle, Newcastle, NSW; l Patient Partner, Montreal, QC; m Women’s Age Lab, Women’s College Hospital, Toronto, ON; n Department of Medicine and Dalla Lana School of Public Health, University of Toronto, Toronto, ON; o National Assembly Library, Quebec City, QC.

**Keywords:** quality reporting, reporting guideline, scale and spread, scaling, systematic review

## Abstract

**Background:**

Quality reporting contributes to effective translation of health research in practice and policy. As an initial step in the development of a reporting guideline for scaling, the Standards for reporting stUdies of sCaling evidenCEd-informED interventions (SUCCEED), we performed a systematic review to identify relevant guidelines and compile a list of potential items.

**Methods:**

We conducted a systematic review according to Cochrane method guidelines. We searched the following databases: MEDLINE, Embase, PsycINFO, Cochrane Library, CINAHL, Web of Science, from their respective inceptions. We also searched websites of relevant organizations and Google. We included any document that provided instructions or recommendations, e.g., reporting guideline, checklist, guidance, framework, standard; could inform the design or reporting of scaling interventions; and related to the health sector. We extracted characteristics of the included guidelines and assessed their methodological quality using a 3-item internal validity assessment tool. We extracted all items from the guidelines and classified them according to the main sections of reporting guidelines (title, abstract, introduction, methods, results, discussion and other information). We performed a narrative synthesis based on descriptive statistics.

**Results:**

Of 7704 records screened (published between 1999 and 2019), we included 39 guidelines, from which data were extracted from 57 reports. Of the 39 guidelines, 17 were for designing scaling interventions and 22 for reporting implementation interventions. At least one female author was listed in 31 guidelines, and 21 first authors were female. None of the authors belonged to the patient stakeholder group. Only one guideline clearly identified a patient as having participated in the consensus process. More than half the guidelines (56%) had been developed using an evidence-based process. In total, 750 items were extracted from the 39 guidelines and distributed into the 7 main sections.

**Conclusion:**

Relevant items identified could inform the development of a reporting guideline for scaling studies of evidence-based health interventions. This and our assessment of guidelines could contribute to better reporting in the science and practice of scaling.

## 1. Introduction

Health interventions that have been shown to be effective need to be scaled to maximize their potential impact on improving population health outcomes and promoting health equity. Hence there is growing interest in the science and practice of scaling.^[1–8]^ In this project, we define the generic term “scaling” as a systematic process to broaden the reach and impact of evidence-based interventions so as to expand their benefits to individuals and society.^[5,9,10]^

The consistent and transparent reporting of health research can facilitate the use of study findings in making decisions about health policy and practice-based decision making. However, deficiencies in the quality of reporting of health research is well documented in the scientific and medical literature broadly. We also note a number of deficiencies in the reporting of scaling studies, e.g., in the areas of ethical and technical justification, scalability assessment, gender equity, and patient and public involvement.^[1,5]^

Reporting guidelines seek to address these deficiencies and facilitate adequate reporting of studies.^[11]^ For example, a Cochrane systematic review showed that 25 out of 27 items of the Consolidated standards of reporting trials (CONSORT) statement were more completely reported in trials published in journals that endorsed the CONSORT than in trials published in journals that did not.^[12]^ Reporting guidelines are used by authors during protocol development and manuscript preparation, by journal editors and peer-reviewers to assess quality reporting, and by readers when synthesizing the literature.

The Standards for reporting stUdies of sCaling evidenCEd-informED interventions (SUCCEED) project was initiated to help improve the reporting of scaling studies, as detailed in the published protocol.^[5]^ An initial step in the development of the SUCCEED reporting guideline was to systematically compile a list of potential items to include.^[13]^ The aim of this systematic review was to document and analyze guidelines relevant to scaling studies and extract relevant items for the development of the SUCCEED guideline.

## 2. Methods

We conducted the systematic review according to Cochrane method guidelines.^[14]^ The protocol of the review was registered with the Open Science Framework (https://osf.io/vcwfx/) and is published elsewhere.^[5]^ We followed the Preferred Reporting Items for Systematic Reviews and Meta-Analyses (PRISMA) statement to guide the report.^[15]^ This study does not involve human participants and ethical approval was not required.

### 2.1. Eligibility criteria

We included any document that provided instructions or recommendations, e.g. a reporting guideline, checklist, guidance, framework, standard (referred thereafter as “guideline”); that could inform the design or reporting of scaling interventions; and was within the health sector. We included guidelines for reporting implementation interventions with the expectation that they could supply additional items relevant to scaling such as implementation strategies. We excluded documents such as formatting instructions produced by journal editors and publishers (Table 1).

**Table 1:** Selection criteria for guidelines for designing and/or reporting scaling and implementation interventions.

Criteria	Inclusion	Exclusion
Type of document	The document provides instructions or recommendations, e.g., reporting guideline, checklist, guidance, framework, standard. The document includes a list of the recommendations or a link to them.	Instructions to authors by journal editors. Does not include a list of recommendations.
Purpose of guidelines	The document informs the design (conduct, planning) or the reporting of scaling interventions. Synonyms of scaling include scale up, scale out, roll out, spread. The document informs the reporting of implementation of interventions.	Is not about scaling or implementation.
Domain	Health	Other domains

### 2.2. Information sources and search strategy

The search strategy was developed by our information specialist for MEDLINE, with iterative revisions by members of the research team and validation by a second information specialist using a Peer Review of Electronic Search Strategies tool^[16]^ before being translated into the other electronic databases.^[5]^ The full search strategy is provided in File S1, Supplemental Digital Content, http://links.lww.com/MD/L359. MEDLINE (Ovid), Embase (Elsevier), PsycINFO (Ovid), Cochrane Library (Methodology Register), CINAHL (EBSCOhost), and Web of Science were searched from their respective inceptions. The initial search was completed in May 2019.

For gray literature, we searched websites of relevant organizations (File S2, Supplemental Digital Content, http://links.lww.com/MD/L360) using the following terms: scaling up, scaling out, spread, scale up, scale out, upscaling, scalability, dissemination, diffusion, implementation. We also used the Google search engine, limiting results to the first 200 hits, after anonymizing the Google search link and applying the PDF filter to reduce the level of noise. For this search, we used each of the above-mentioned terms in combination with “reporting” and “health.” Finally, we searched the reference lists of identified reports.

### 2.3. Study selection

Results from the electronic databases were managed through Endnote X9 to identify duplicates. Records were then uploaded to Covidence, an Internet-based system, for independent screening. Four reviewers (A.G., A.B.C., G.E., and J.S.) independently screened a random sample of 5% of records to pilot test the eligibility criteria based on titles and abstracts. The eligibility form was validated with the members of the SUCCEED Executive Committee before pairs of the same 4 reviewers independently screened all the remaining records for titles and abstracts. Two reviewers (A.G. and G.E.) independently assessed the full texts. Discrepancies were resolved by consensus through discussion.

### 2.4. Data collection process

First, we developed a data dictionary informed by the Cochrane Checklist of items to consider in data collection^[17]^ and guidelines to design scaling interventions.^[6,9,18]^ The dictionary was enriched with elements supplied by members of the Executive Committee from their experience in the development of reporting guidelines and the science and practice of scaling. We then created an Excel extraction form from the data dictionary that was pilot tested by 3 reviewers (A.G., G.E., and O.A.). Pairs of the same reviewers independently extracted data from included guidelines and related reports. For each guideline, we retrieved all the necessary documents to assimilate the information required for extraction as follows:

1. We systematically searched for the guideline statement, the explanatory document, and any documents related to the development of the guideline (i.e., systematic reviews, Delphi studies).
2. We explored the website of the guideline or of the group that developed the guideline, if available.
3. We also retrieved any cited appendices or supplementary documents.

Extracted data included:

• General characteristics (e.g., title, name of the guideline, number and sex of the authors and if first author, corresponding author and contact information including geographic location).
• Type of the guideline (e.g., for designing scaling interventions).
• Elements of the development process: theoretical framework; type of data collection (e.g., systematic review); consensus process, (e.g., Delphi) and panelists in consensus group (number, sex and type); validation or not.
• Stakeholder involvement, i.e. number, sex and type (clinician, decision maker, journal editor/publisher, funder, patient, researcher).
• Number and description of the items (for each included guideline, we extracted all items).
• Presence of sex- and gender-related words and their correct use.^[19]^
• Other information, e.g., funding source: public (governmental/intergovernmental), private (profit/nonprofit such as charities), public/private, and not provided (no funding as stated by the authors or no information); conflict of interest.

Disagreements were resolved by consensus or by discussion with a third reviewer (H.T.V.Z.).

### 2.5. Methodological quality assessment

We used a 3-item internal validity assessment tool to assess the methodological quality of development of the guidelines. The tool had been developed by members of the research team during a systematic review of sex and gender considerations in reporting guidelines.^[20]^ Each of the following questions was coded “yes,” “no” or “unclear”: Did the developers of the guideline represent more than one stakeholder group? Did the developers report gathering any data for the creation of the guideline? and Did the developers report the use of a consensus process? Pairs of 3 reviewer authors of the tool (A.G., G.E., and H.T.V.Z.) independently conducted the assessment and any disagreements were resolved by consensus.

### 2.6. Data analysis and synthesis

We analyzed extracted data using descriptive statistics (numbers and percentages) and performed a narrative synthesis of the guidelines and items.

For the guidelines, we provided the numbers and percentages by type. We reported numbers and percentages of authors by stakeholder group (e.g., clinician, decision maker, journal editor, funder, patient, researcher) and by sex. We graphically synthesized the 3 criteria for methodological quality of the guidelines’ development, and gave it an overall rating of high internal validity (i.e., low risk of bias) if the number of “yes” ≥ 2 and low internal validity (i.e., high risk of bias) if the number of “yes” < 2.

The items generated were categorized into the main sections of any reporting guideline: title, abstract, introduction, methods, results, discussion and other information. For items extracted from reporting guidelines, we reported the categories as presented in their statements or checklists. For the classification of items extracted from the other type of guidelines (for designing scaling interventions), 4 review authors of several publications on scaling^[1,5,21–24]^ (A.G., A.B.C., G.E., and H.T.V.Z.) conducted 2 pilot rounds. The classification was performed independently by pairs of 3 reviewers (A.G., G.E., and H.T.V.Z.) and disagreements were resolved through discussion. We calculated the percentage of items by type of guidelines included and by the main sections of a reporting guideline. SAS (SAS, version 9.4, Institute, Cary, NC) and Excel (Microsoft) were used to perform the analyses.

### 2.7. Patient and public involvement

We included 2 patient partners (E.A., K.P.) when establishing the Executive Committee, which is the first step recommended for developing health research reporting guidelines.^[13]^ As members of the research team, they were coauthors on the published protocol^[5]^ and provided feedback at each stage described in the methods: search strategy, study selection criteria, data extraction dictionary, and data synthesis. Given the methodological nature of the project and to optimize their participation, with their agreement we held working sessions to go through documents prior to Executive Committee meetings. They will continue to be involved in the subsequent steps of the development of SUCCEED reporting guideline, including the consensus process and the dissemination.

## 3. Results

### 3.1. Study selection

**Figure 1.**
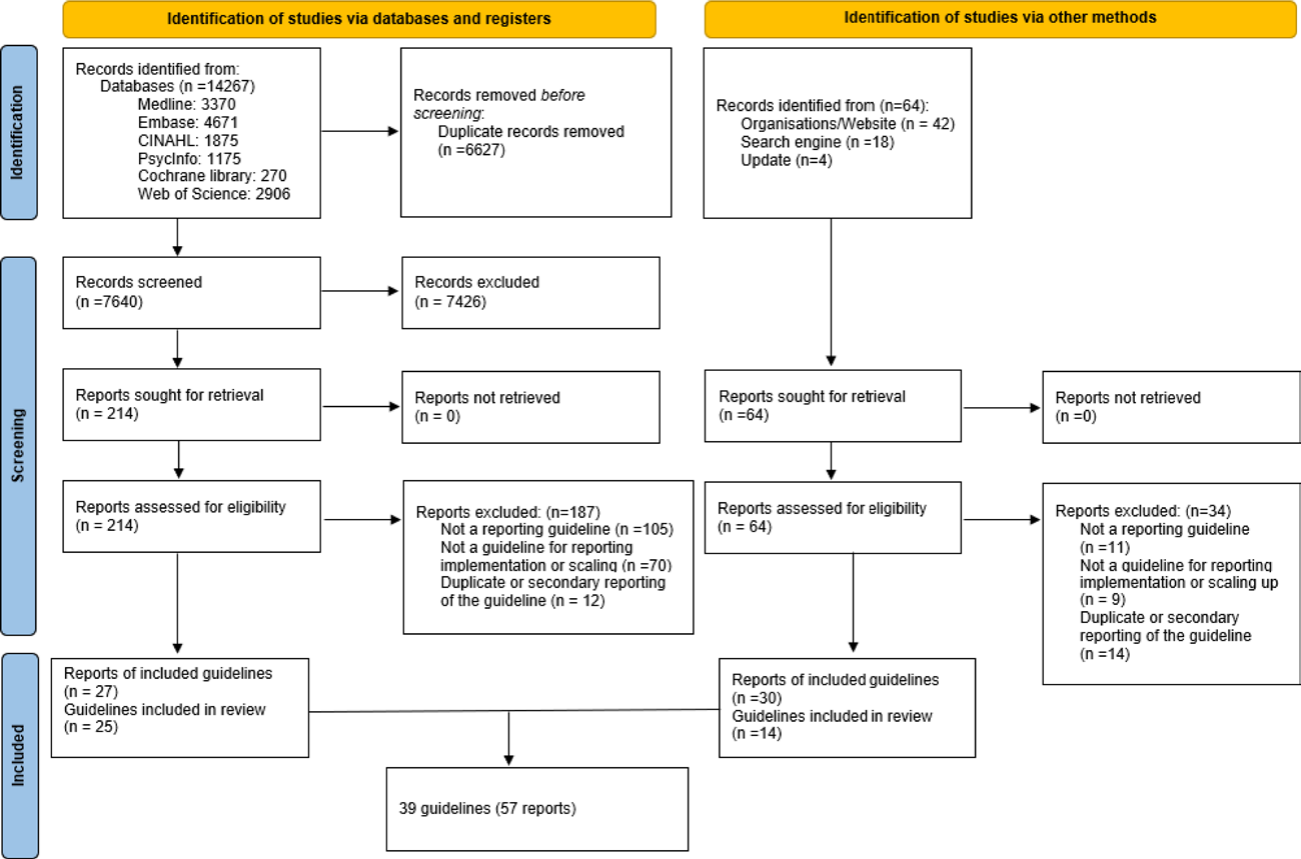
PRISMA 2020 flow diagram of new systematic reviews which included searches of databases, registers and other sources.

The PRISMA flow diagram (Fig. 1) was used to report the selection process. We included 39 guidelines (Table 2) whose data were extracted from 57 reports. The list of excluded reports is provided in File S3, Supplemental Digital Content, http://links.lww.com/MD/L361

**Table 2:** Characteristics of included guidelines and internal validity.

Reference Name* (Year) ^Ref.^ Country	Related reports [Ref.]	Focus i) Designing scaling intervention ii) Reporting implementation intervention	Number of items	Funding source†	Conflict of interest information (Yes, No)	Internal validity‡ (High, Low)
Reach, efficacy, adoption, implementation, and maintenance framework (1999)^[25]^ United States of America	^[26]^	i	31	Public	No	Low
USAID and Management Sciences for Health (2002)^[27]^ United States of America	–	i	10	No info	No	Low
Reviewer Guidelines for Reports of Public Health Interventions (2003)^[28]^ Canada	–	ii	19	No info	No	Low
Transparent Reporting of Evaluations with nonrandomized Designs (2004)^[29]^ United States of America	–	ii	58	No info	No	High
ExpandNet/WHO framework for scaling up (2007)^[8]^ Switzerland	^[18,30–32]^	i	27	Public/private	No	High
Riley et al (2008)^[33]^ Canada	–	ii	16	Public/private	Yes	Low
Egan et al (2009)^[34]^ United Kingdom	–	ii	10	Public	Yes	Low
Framework for reporting health service delivery models for managing rheumatoid arthritis (2010)^[35]^ Canada	^[36]^	ii	10		Yes	High
Bryce et al (2011)^[37]^ United States of America	–	i	7	Public/private	Yes	High
Conn & Groves (2011)^[38]^ United States of America	–	ii	5	Public	No	Low
Eaton et al (2011)^[39]^ Nigeria	–	i	9	No info	Yes	High
WHO/ExpandNet (2011)^[40]^ Switzerland	–	i	12	Public/private	No	High
Framework for explaining successful scale-up (2011)^[41]^ United States of America	–	i	6	No info	Yes	Low
AIDED model for scale-up (2012)^[42]^ United States of America	–	i	5	Private	Yes	Low
Reporting standards for studies of tailored interventions (2012)^[43]^ United States of America	–	ii	7	No info	Yes	Low
Guide for Monitoring Scale-up of Health Practices and Interventions (2013)^[44]^ United States of America	–	i	10	No info	No	Low
Workgroup for Intervention Development and Evaluation Research recommendations (2013)^[45]^ Canada	^[46]^	ii	20	Public	Yes	High
Duncan et al (2013)^[47]^ United States of America	–	ii	21	No info	No	Low
The Oxford Implementation Index (2013)^[48]^ United Kingdom	–	ii	32	Public	Yes	High
Proctor et al (2013)^[49]^ United States of America	–	ii	10	Public	Yes	Low
Dickson et al (2014)^[50]^ United States of America	–	i	4	Public/private	Yes	High
Template for intervention description and replication (2014)^[51]^ United Kingdom	–	ii	12	Public/private	Yes	High
Global framework implementation criteria for pilot test (2014)^[52]^ United States of America	–	ii	17	Public	Yes	High
Multiplicative scale-up framework (2014)^[53]^ Switzerland	–	i	17	No info	No	Low
mHealth Assessment and Planning for Scale (2015)^[54]^ Switzerland	–	i	38	Public/private	No	High
Neta et al (2015)^[55]^ United States of America	–	ii	30	Public	No	High
Guidelines for Reporting Evaluations based on Observational Methodology (2015)^[56]^ Spain	–	ii	14	Public	No	High
CCDR (2016)^[57]^ Canada	–	ii	20	No info	No	Low
Barker et al (2016)^[58]^ United States of America	–	i	4	Private	Yes	Low
Scaling Up Management Framework (2016)^[59]^ United States of America	–	i	14	Private	No	Low
Hales et al (2016)^[60]^ Switzerland	^[61]^	ii	64	Public	Yes	High
Milat et al (2016)^[6]^ Australia	^[62–64]^	i	20	Public	Yes	High
Standards for QUality Improvement Reporting Excellence (2016)^[65]^ United States of America	^[66–69]^	ii	40	Private	Yes	High
Indig et al (2017)^[70]^ Australia	–	i	6	Public	Yes	High
Programme Reporting Standards (2017)^[71]^ Switzerland	–	ii	47	Public	Yes	High
Standards for Reporting Implementation Studies (2017)^[72]^ United Kingdom	^[73,74]^	ii	37	Public	Yes	High
Consolidated advice for reporting ECD implementation research (2018)^[75]^ United States of America	–	ii	21	Public	Yes	High
McLean & Gargani (2019)^[9]^ Canada	^[76]^	i	12	Public	No	High
Reeves et al (2019)^[77]^ Australia	–	ii	8	No info	Yes	Low

AIDED = model for scale up of family health innovations, CCDR = Canada Communicable Disease Report, USAID = United States Agency for International Development, WHO = World Health Organization.

* Authors or organizations if no name was identified.

† Public (governmental/intergovernmental), private (for profit/no-profit); no info (no info or no funding declared).

‡ Based on a 3-item internal validity assessment tool (high if ≥2 “yes” and low if <2 “yes”).

### 3.2. Characteristics of the included guidelines

Included guidelines were published between 1999 and 2019. They originated in 7 countries, which were identified according to the location of the corresponding author’s institution or the location of the institution if no authors were listed (Table 2, File S4A, Supplemental Digital Content, http://links.lww.com/MD/L364). Regarding the type of guidelines, 43.6% (17/39) were guidelines to design scaling interventions and 56.4% (22/39) for reporting implementation interventions. None was found for reporting scaling interventions. Seventy-seven percent (30/39) mentioned the funding source and 59% (23/39) included a statement of conflict of interest. Most guidelines (41%, 16/39) were developed with public funding, while the private sector funded the fewest (13%, 5/39); combined public/private funding was used for 18% (7/39) and no information or source of funding were reported for 28% (11/39).

Regarding sex and patient representation in the study teams, at least one female author was listed on 80% (31/39) of included guidelines and as first author on 54 % (21/39), while at least one male author was listed on 72% (28/39). Seven guidelines were by female authors, and 4 guidelines by male authors only (Files S4 and S5, Supplemental Digital Content, http://links.lww.com/MD/L364 and http://links.lww.com/MD/L368). None of the authors belonged to the patient stakeholder group (File S6, Supplemental Digital Content, http://links.lww.com/MD/L369). Regarding sex and gender in guideline content, only one guideline (3%) mentioned at least one sex-related word and 3 (8%) mentioned at least one gender-related word. Of these 3 gender-related words, only one was used correctly (File S4A, Supplemental Digital Content, http://links.lww.com/MD/L364).

### 3.3. Development process and internal validity assessment of the guidelines

The development of the guidelines was predominantly based on data gathering through literature reviews (49%, 18/39) and systematic reviews (24%, 9/39). Only 13% (5/39) of the guidelines were developed using a theoretical framework. A consensus process was used in the development of 18 (46.2%) guidelines. The most common type of consensus was informal consensus (22%, 8/39) followed by Delphi (15.4%, 6/39) (Table 3, File S4A, Supplemental Digital Content, http://links.lww.com/MD/L364). Only one guideline (3%) clearly identified a patient as a panelist (File S7, Supplemental Digital Content, http://links.lww.com/MD/L371). Twenty-six percent of guidelines (10/39) used a validation process (pilot testing and/or expert feedback).

**Table 3:** Elements of the development process of included guidelines.

	Designing scaling interventions N = 17 (%)	Reporting implementation interventions N = 22 (%)	Total N = 39 (%)
Theoretical framework			
Yes	3 (18)	2 (9)	5 (13)
No	14 (82)	20 (91)	34 (87)
Type of data gathering*			
Systematic review	2 (13)	7 (32)	9 (24)
Other literature review	8 (53)	10 (45)	18 (49)
Qualitative	2 (13)	0 (0)	2 (5)
Other data source†	5 (29)	2 (9)	7 (18)
Consensus process*			
Delphi	1 (6)	5 (23)	6 (15)
Informal consensus	4 (27)	4 (18)	8 (22)
Consensus conference	0 (0)	4 (18)	4 (11)
Validation			
Yes	3 (18)	7 (32)	10 (26)
No	14 (82)	15 (68)	29 (74)

* Did not add up for only ‘yes’ value was reported.

† e.g., expert opinion, field work.

**Figure 2.**
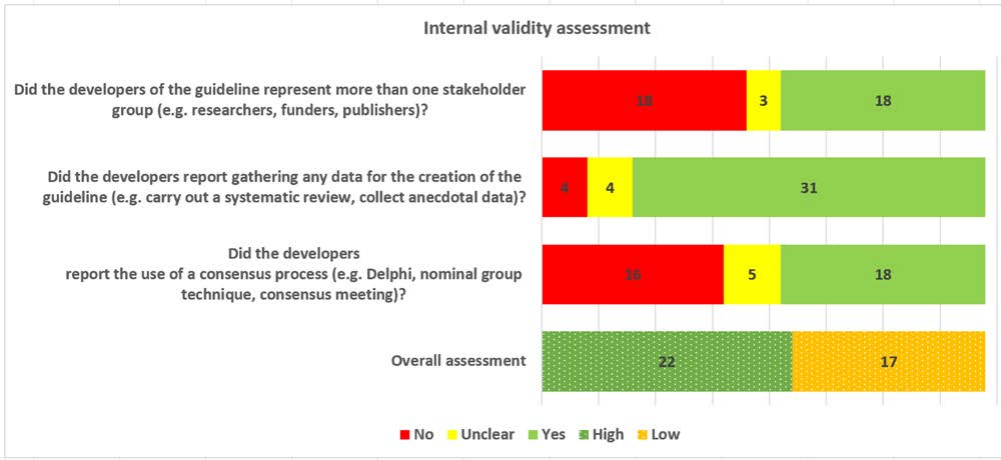
Summary of internal validity assessment.

A summary of our internal validity assessment, based on our 3-item tool, is presented in Figure 2. Development was evidence-based (high internal validity) in 56.4% (22/39) of the guidelines, and only in those developed since 2004 (Fig. 2, Table 2, File S8, Supplemental Digital Content, http://links.lww.com/MD/L372). The inclusion of more than one stakeholder group was the least fulfilled of the 3 validity criteria (46.2%, 18/39).

### 3.4. Identification and classification of potentially relevant items

In total, 750 items were extracted from the 39 guidelines and related documents with a mean number of 19.2 items per guideline (File S4B, Supplemental Digital Content, http://links.lww.com/MD/L367 for the full list of items). The numbers and means of items were 232 (13.6) for the guidelines for designing scaling interventions and 518 (23.5) for the guidelines for reporting implementation interventions. The distribution of the items per section of reporting guidelines is shown in Table 4. Sections that had the most items were “methods” (n = 519, 69%), “results” (n = 142, 19%) and “discussion” (n = 97, 13%). Examples of items identified for the methods section that have potential for inclusion in a scaling reporting guideline were:

**Table 4:** Distribution of items by section of a reporting guideline.

Section	Designing scaling interventions N (%) [232 items]	Reporting implementation interventions N (%) [518 items]	Total N (%) [750 items]
Title	0 (0)	20 (4)	20 (3)
Abstract	16 (7)	51 (10)	67 (9)
Introduction	34 (15)	58 (11)	92 (12)
Methods	207 (89)	312 (60)	519 (69)
Results	33 (14)	109 (21)	142 (19)
Discussion	15 (6)	82 (16)	97 (13)
Other information	0 (0)	9 (2)	9 (1)

- Engage in a participatory process involving key stakeholders^[78]^;
- Adopt an approach to scaling up: specify the chosen delivery strategy (e.g., vertical, horizontal, cascade or phased approaches)^[6,8,41]^;
- Consider optimal scale (balances the magnitude, variety, sustainability, and equity of impacts in ways stakeholders endorse)^[9]^;
- Consider how much it will cost to scale up (e.g., estimating start-up costs, long-term running costs, economies of scale, the cost of alternatives).^[79]^

## 4. Discussion

An initial step in the development of a reporting guideline is the systematic review of the literature to identify potential items to include. To achieve this, we identified 39 guidelines: 22 for reporting implementation interventions and 17 for designing scaling interventions. We found no guidelines for reporting scaling studies. All but one guideline developer were located in high-income countries. No patient was listed as author, and of guideline developers who used a consensus process, only one included a patient as panelist. The 39 guidelines and their associated documents yielded 750 items distributed among 7 main sections of a reporting guideline.

We identified 2 types of guidelines. The first consisted of standards for reporting implementation interventions. Examples among the most recently published included Workgroup for Intervention Development and Evaluation Research, a Template for Intervention Description and Replication, Standards for Reporting Implementation Studies and Programme Reporting Standards,^[45,51,71,72]^ with the expectation that they would improve aspects such as quality intervention reporting and the reporting of implementation strategies (scaling requires implementation strategies but not all implementation is scaling) and of any evidence-based interventions of interest.^[80,81]^ However, they did not address some elements specific to scaling.^[5]^ These elements were provided by the second type of guidelines, i.e. those that offered recommendations or steps for designing scaling interventions. Most of these guidelines were developed by nonacademic organizations that work mainly in low- and middle-income countries where most scaling programs occur.^[1,10]^ Our assessment of evidence-based development of guidelines for designing scaling interventions would help actors in the field select the most appropriate ones while developing their scaling programs.

We found that none of the included guidelines involved patients in the development process as authors and only one included a patient as a panelist in the consensus process, despite the growing literature on the importance of patient engagement in health research. Interestingly, patients themselves expressed their willingness to be involved^[82,83]^ and most journal editors support the nomination of patient partners as authors or coauthors on published biomedical research articles.^[84]^ While recent years have seen an increase in the involvement of patients and members of the public in health research, they are rarely involved in methodological projects including in the development of reporting guidelines and in the science of implementation and scaling.^[5,21]^ As such, the development of the SUCCEED project is innovative as it includes 2 patient partners on the Executive Committee. This corresponds to the highest level of patient engagement according to the continuum of engagement.^[85]^ In addition, patients and members of the public will be recruited for the consensus process. One output from the involvement of patient partners in this research project was the development of a training presentation entitled “*Initiation aux étapes d’une revue systématique: Implication des patientes et patients partenaires*” [Introduction to the steps of a systematic review: Involvement of patient partners].

Items from 3 guidelines that did not meet the eligibility criteria for this systematic review will nevertheless be included in the consensus process because of their relevance to the development of the SUCCEED guideline. These 3 guidelines are Guidance for Reporting Involvement of Patients and the Public tools to improve reporting of patient and public involvement in research^[86]^; Sex and Gender Equity in Research guidelines ^[87]^; and The CONSORT extension for the reporting of stepped wedge cluster randomized trials.^[88]^ Items from the first two will be added because they address equity concerns, and the third because the stepped wedge design is considered one of the most appropriate for scaling studies.^[22]^ Given the growth of open science in recent years and in accordance with recommended practices,^[89,90]^ we will also formulate and add some items related to preregistration and data sharing practices. Indeed, an increasing number of funding agencies are requiring data management plans including data sharing statements^[90,91]^ and are consequently relevant for reporting guidelines. Some existing reporting guidelines (e.g., PRISMA) were updated or are being updated (e.g., CONSORT) accordingly.^[15]^

There are a few limitations to note. Scaling is a growing field, including the concept of “the science of scaling”^[5]^ and there is no consensus on terminology and frameworks. As such, we may not have identified all relevant guidelines on scaling. As our search for this study was completed in 2019 we may have missed recent scaling guidelines or work that is still in progress or not yet published. However, our identified guidelines included the framework (WHO/ExpandNet framework) that as of 2022 is still the one most frequently used by actors developing and reporting scaling programs.^[10]^ We will also enrich the list of items by reviewing studies included in our umbrella review on evidence on scaling in health and social care^[10]^ and with the input of experts in the field during our forthcoming international consensus study. Finally, we will conduct living reviews^[4]^ to incorporate new items as the science evolves.

## 5. Conclusion

This review identified relevant guidelines for the development of the SUCCEED reporting guideline. We assessed the evidence-based development of included guidelines, allowing the actors in the science of scaling to choose the best guidelines to inform their program design. The generated list of items will be enriched through a consensus process. Findings from this study and those of subsequent steps in the development of the SUCCEED guideline will help increase awareness of the importance of quality reporting and contribute to a better understanding of the science and practice of scaling health innovations.

## Acknowledgments

We thank Becky Skidmore, MLS for peer review of the MEDLINE search strategy. We thank Jasmine Sawadogo (JS) and Odilon Assan (OA) for their contributions in the screening of records or data extraction. We also thank Louisa Blair for editing this manuscript.

## Author contributions

**Conceptualization:** Amédé Gogovor, Hervé Tchala Vignon Zomahoun, Ali Ben Charif, David Moher, Robert K. D. McLean, Andrew Milat, Luke Wolfenden, Karina Prévost, Emmanuelle Aubin, Paula Rochon, France Légaré.

**Data curation:** Giraud Ekanmian, Nathalie Rheault.

**Formal analysis:** Giraud Ekanmian.

**Funding acquisition:** France Légaré.

**Investigation:** Amédé Gogovor, Hervé Tchala Vignon Zomahoun, Ali Ben Charif, David Moher, Robert K. D. McLean, Andrew Milat, Luke Wolfenden, Karina Prévost, Emmanuelle Aubin, Paula Rochon, Nathalie Rheault, France Légaré.

**Methodology:** Amédé Gogovor, Hervé Tchala Vignon Zomahoun, Ali Ben Charif, David Moher, Robert K. D. McLean, Andrew Milat, Luke Wolfenden, Paula Rochon, France Légaré.

**Supervision:** Amédé Gogovor, Hervé Tchala Vignon Zomahoun, David Moher, France Légaré.

**Writing – original draft:** Amédé Gogovor.

**Writing – review & editing:** Amédé Gogovor, Hervé Tchala Vignon Zomahoun, Ali Ben Charif, Giraud Ekanmian, David Moher, Robert K. D. McLean, Andrew Milat, Luke Wolfenden, Karina Prévost, Emmanuelle Aubin, Paula Rochon, Nathalie Rheault, France Légaré.

## Supplementary Material


















