# Supplementary material for: Informing the development of the SUCCEED reporting guideline for studies on the scaling of health interventions: A systematic review
Source: Medicine (Baltimore). 2024 Feb 16;103(7):e37079. doi: 10.1097/MD.0000000000037079 (PMC10869056; doi:10.1097/MD.0000000000037079)
Supplement: Supplementary file 1 [file medi-103-e37079-s001.docx]

### Medline Ovid (2019-05-09)

| **Concepts** | **Search strategy keywords** | **Search** | **# Results** |
| --- | --- | --- | --- |
| Reporting standard (Controlled vocabulary) | exp Writing/st [Standards] | #1 | 2 797 |
| Reporting standard (Free text) | (((((good or best) adj2 practi#e*) or guide or guides or guideline* or checklist* or "check list*" or framework* or standard* or recommend* or guidance* or requirement* or instruct* or consensus or "aide memoir*" or quality or "worked example*" or criteri* or critique* or design or designs or clarity or statement* or parameter* or advice* or policy or policies or accura* or appropriate* or "minimum information" or tool* or complete or protocol* or advice* or uniform* or better or strengthen* or transparen*) adj2 (report* or writ*)) or "research standard*" or "publishing standard*" ).ti.  or (((((good or best) adj2 practi#e*) or guide or guides or guideline* or checklist* or "check list*" or framework* or standard* or recommend* or guidance* or requirement* or instruct* or consensus or "aide memoir*" or quality or "worked example*" or criteri* or critique* or design or designs or clarity or statement* or parameter* or advice* or policy or policies or accura* or appropriate* or "minimum information" or tool* or complete or protocol* or advice* or uniform* or better or strengthen* or transparen*) adj2 (report* or writ*)) or "research standard*" or "publishing standard*").ab.  or ( ((((good or best) adj2 practi#e*) or guide or guides or guideline* or checklist* or "check list*" or framework* or standard* or recommend* or guidance* or requirement* or instruct* or consensus or "aide memoir*" or quality or "worked example*" or criteri* or critique* or design or designs or clarity or statement* or parameter* or advice* or policy or policies or accura* or appropriate* or "minimum information" or tool* or complete or protocol* or advice* or uniform* or better or strengthen* or transparen*) adj2 (report* or writ*)) or "research standard*" or "publishing standard*").kf. | #2 | 67 164 |
| Reporting standard | 1 or 2 | #3 | 69 466 |
| Scaling (Controlled vocabulary) | Not available | NA | - |
| Scaling (Free text) | (("scaling" or widespread or spread$ or spreading or "rolling out" or "roll out" or "rolls out" or "rolled out" or "scale$ up" or "scale$ out" or upscaling or scalability or scalable) adj5 (innovation$ or intervention$ or technolog* or practice* or care or initiative* or program* or product* or therap* or service*)).ti.  or (("scaling" or widespread or spread$ or spreading or "rolling out" or "roll out" or "rolls out" or "rolled out" or "scale$ up" or "scale$ out" or upscaling or scalability or scalable) adj5 (innovation$ or intervention$ or technolog* or practice* or care or initiative* or program* or product* or therap* or service*)).ab.  or (("scaling" or widespread or spread$ or spreading or "rolling out" or "roll out" or "rolls out" or "rolled out" or "scale$ up" or "scale$ out" or upscaling or scalability or scalable) adj5 (innovation$ or intervention$ or technolog* or practice* or care or initiative* or program* or product* or therap* or service*)).kf | #4 | 22 017 |
|  | ((bring* or brought or taking or take* or increas* or going or implement* or econom*) adj5 scal* adj5 (innovation$ or intervention$ or technolog* or practice* or care or initiative* or program* or product* or therap* or service*)).ti.  or ((bring* or brought or taking or take* or increas* or going or implement* or econom*) adj5 scal* adj5 (innovation$ or intervention$ or technolog* or practice* or care or initiative* or program* or product* or therap* or service*)).ab.  or ((bring* or brought or taking or take* or increas* or going or implement* or econom*) adj5 scal* adj5 (innovation$ or intervention$ or technolog* or practice* or care or initiative* or program* or product* or therap* or service*)).kf. | #5 | 2 123 |
|  | ("reverse innovation*" or "trickle-up innovation*").ti. or ("reverse innovation*" or "trickle-up innovation*").ab. or ( "reverse innovation*" or "trickle-up innovation*" ).kf. | #6 | 40 |
|  | (transfer* adj5 (innovation$ or intervention$ or technolog* or initiative*)).ti. or (transfer* adj5 (innovation$ or intervention$ or technolog* or initiative*)).ab. or (transfer* adj5 (innovation$ or intervention$ or technolog* or initiative*)).kf. | #7 | 4 787 |
|  | 4 or 5 or 6 or 7 | #8 | 28 469 |
| Implementation (Controlled vocabulary) | Translational medical research/ or Information Dissemination/ or exp "diffusion of innovation"/ or exp Knowledge Management/ | #9 | 42 399 |
| Implementation  (Free text) | (("knowledge to action" or "continuing education" or "know-do" or implement* or dissemin* or diffus* or adopt* or adapt*) adj5 (research or science or bioscience or biomedic* or innovation* or intervention* or technolog* or practice* or care or initiative* or program* or product* or therap* or service*)).ti.  or (("knowledge to action" or "continuing education" or "know-do" or implement* or dissemin* or diffus* or adopt* or adapt*) adj5 (research or science or bioscience or biomedic* or innovation* or intervention* or technolog* or practice* or care or initiative* or program* or product* or therap* or service*)).ab.  or (("knowledge to action" or "continuing education" or "know-do" or implement* or dissemin* or diffus* or adopt* or adapt*) adj5 (research or science or bioscience or biomedic* or innovation* or intervention* or technolog* or practice* or care or initiative* or program* or product* or therap* or service*)).kf. | #10 | 176 598 |
|  | ((applied or mediation* or translat*) adj5 (research or science or bioscience or biomedic*)).ti.  or ((applied or mediation* or translat*) adj5 (research or science or bioscience or biomedic*)).ab.  or ((applied or mediation* or translat*) adj5 (research or science or bioscience or biomedic*) ).kf | #11 | 30 458 |
|  | ((mediation* or translat* or populari#ation or populari#e or populari#ed or populari#ing) adj5 (innovation* or intervention* or technolog* or practice* or care or initiative* or program* or product* or therap* or service*)).ti.  or ((mediation* or translat* or populari#ation or populari#e or populari#ed or populari#ing) adj5 (innovation* or intervention* or technolog* or practice* or care or initiative* or program* or product* or therap* or service*)).ab.  or ((mediation* or translat* or populari#ation or populari#e or populari#ed or populari#ing) adj5 (innovation* or intervention* or technolog* or practice* or care or initiative* or program* or product* or therap* or service*)).kf. | #12 | 26 806 |
|  | (knowledge) adj3 (transfer* or translat* or broker* or mobil* or uptake or "up take" or adapt* or dispers* or exchange* or application or utili#ation or utili#e or utili#ed or utili#ing or communicat* or cycle? or transform* or action? or manage*)) adj5 (innovation* or intervention* or technolog* or practice* or care or initiative* or program* or product* or therap* or service*)).ti.  or (knowledge adj3 (transfer* or translat* or broker* or mobile* or uptake or "up take" or adapt* or dispers* or exchange* or application or utili#ation or communicat* or cycle? or transform* or action? or manage*) adj5 (innovation* or intervention* or technolog* or practice* or care or initiative* or program* or product* or therap* or service*)).ab.  or (knowledge adj3 (transfer* or translat* or broker* or mobile* or uptake or "up take" or adapt* or dispers* or exchange* or application or utili#ation or communicat* or cycle? or transform* or action? or manage*) adj5 (innovation* or intervention* or technolog* or practice* or care or initiative* or program* or product* or therap* or service*)).kf. | #13 | 4 265 |
| Implementation (Free text) | 10 or 11 or 12 or 13 | #14 | 228 168 |
| Implementation | 9 or 14 | #15 | 260 622 |
| Implementation or scaling up | 8 or 15 | #16 | 283 800 |
| Total result | 3 and 16 | #17 | 3 410 |
| Filter for human | exp Animals/ NOT exp Humans/ | #18 | 4 576 104 |
| With filter for human | 17 NOT 18 | #19 | 3 370 |

### Embase Elsevier (2019-05-09)

| **Concepts** | **Search strategy keywords** | **Search** | **# Results** |
| --- | --- | --- | --- |
| Reporting standard (Controlled vocabulary) | 'reporting'/exp | #1 | 13 |
| Reporting standard (Free text) | (( "good practise*" or "good practice*" or "best practise*" or "best practice*" ) NEAR/2 (report* or writ*)):ti,ab,kw | #2 | 215 |
|  | ((guide or guides or guideline* or checklist* or "check list*" or framework* or standard* or recommend* or guidance* or requirement* or instruct* or consensus or "aide memoir*" or quality or "worked example*" or criteri* or critique* or design or designs or clarity or statement* or parameter* or advice* or policy or policies or accura* or appropriate* or "minimum information" or tool* or complete or protocol* or advice* or uniform* or better or strengthen* or transparen*) NEAR/2 (report* or writ*)):ti,ab,kw | #3 | 92 154 |
|  | ("research standard*" or "publishing standard*"):ti,ab,kw | #4 | 533 |
|  | #2 or #3 or #4 | #5 | 92 809 |
| Reporting standard | #1 or #5 | #6 | 92 819 |
| Scaling (Controlled vocabulary) | 'scale up'/exp or 'scaling'/exp or 'scalability'/exp | #7 | 9 675 |
| Scaling (Free text) | (("scaling" or widespread or spread or "rolling out" or "roll out" or "scale up" or "scale out" or "scaled up" or "scaled out" or upscaling or scalability or scalable) NEAR/5 (innovation* or intervention* or technolog* or practice* or care or initiative* or program* or product* or therap* or service*)):ti,ab,kw | #8 | 26 026 |
|  | ((scal* NEAR/5 (bring* or brought or taking or take* or increas* or going or implement* or econom*)) and (innovation* or intervention* or technolog* or practice* or care or initiative* or program* or product* or therap* or service*)):ti,ab,kw | #9 | 12 301 |
|  | ("reverse innovation*" or "trickle-up innovation*"):ti,ab,kw | #10 | 42 |
|  | ((transfer*) NEAR/5 (innovation* or intervention* or technolog* or initiative*)):ti,ab,kw | #11 | 6 879 |
|  | #8 or #9 or #10 or #11 | #12 | 44 306 |
|  | #7 or #12 | #13 | 51 486 |
| Implementation (Controlled vocabulary) | 'translational research'/exp or 'dissemination'/exp or 'continuing education'/exp or 'implementation science'/exp or 'implementation'/exp or 'implementation scientist'/exp or 'applied research'/exp or 'knowledge management'/exp | #14 | 49 345 |
| Implementation  (Free text) | (("knowledge to action" or "continuing education" or "know-do" or implement* or dissemin* or diffus* or adopt* or adapt*) NEAR/5 (research or science or bioscience or biomedic* or innovation* or intervention* or technolog* or practice* or care or initiative* or program* or product* or therap* or service*)):ti,ab,kw | #15 | 241 959 |
|  | ((applied or mediation* or translat*) NEAR/5 (research or science or bioscience or biomedic*)):ti,ab,kw | #16 | 40 218 |
|  | ((mediation* or translat* or popularisation or popularization or popularise or popularize or popularised or popularized or popularising or popularizing) NEAR/5 (innovation* or intervention* or technolog* or practice* or care or initiative* or program* or product* or therap* or service*)):ti,ab,kw | #17 | 34 866 |
|  | (((knowledge) NEAR/3 (transfer* or translat* or broker* or mobil* or uptake or "up take" or adapt* or dispers* or exchange* or application or utilisation or utilization or utilise or utilize or utilised or utilized or utilising or utilizing or communicat* or cycle or cycles or transform* or action or actions or manage*)) AND (innovation* or intervention* or technolog* or practice* or care or initiative* or program* or product* or therap* or service*)):ti,ab,kw | #18 | 22 342 |
| Implementation (Free text) | #15 or #16 or #17 or #18 | #19 | 317 892 |
| Implementation | #14 or #19 | #20 | 354 068 |
| Implementation or scaling up | #13 or #20 | #21 | 397 864 |
| Total result | #6 and #21 | #22 | 4 622 |
| Filter for human | 'animal'/exp not 'human'/exp | #23 | 5 240 100 |
| With filter for human | #22 NOT #23 | #24 | 4 671 |

### PsycInfo Ovid (2019-05-09)

| **Concepts** | **Search strategy keywords** | **Search** | **# Results** |
| --- | --- | --- | --- |
| Reporting standard (Controlled vocabulary) | Not available | NA | - |
| Reporting standard (Free text) | (((((good or best) adj2 practi#e*) or guide or guides or guideline* or checklist* or "check list*" or framework* or standard* or recommend* or guidance* or requirement* or instruct* or consensus or "aide memoir*" or quality or "worked example*" or criteri* or critique* or design or designs or clarity or statement* or parameter* or advice* or policy or policies or accura* or appropriate* or "minimum information" or tool* or complete or protocol* or advice* or uniform* or better or strengthen* or transparen*) adj2 (report* or writ*)) or "research standard*" or "publishing standard*").ti.  or (((((good or best) adj2 practi#e*) or guide or guides or guideline* or checklist* or "check list*" or framework* or standard* or recommend* or guidance* or requirement* or instruct* or consensus or "aide memoir*" or quality or "worked example*" or criteri* or critique* or design or designs or clarity or statement* or parameter* or advice* or policy or policies or accura* or appropriate* or "minimum information" or tool* or complete or protocol* or advice* or uniform* or better or strengthen* or transparen*) adj2 (report* or writ*)) or "research standard*" or "publishing standard*").ab.  or (((((good or best) adj2 practi#e*) or guide or guides or guideline* or checklist* or "check list*" or framework* or standard* or recommend* or guidance* or requirement* or instruct* or consensus or "aide memoir*" or quality or "worked example*" or criteri* or critique* or design or designs or clarity or statement* or parameter* or advice* or policy or policies or accura* or appropriate* or "minimum information" or tool* or complete or protocol* or advice* or uniform* or better or strengthen* or transparen*) adj2 (report* or writ*)) or "research standard*" or "publishing standard*").hw. | #1 | 21 614 |
| Scaling (Controlled vocabulary) | exp technology transfer/ | #2 | 193 |
| Scaling (Free text) | (("scaling" or widespread or spread$ or spreading or "rolling out" or "roll out" or "rolls out" or "rolled out" or "scale$ up" or "scale$ out" or upscaling or scalability or scalable) adj5 (innovation$ or intervention$ or technolog* or practice* or care or initiative* or program* or product* or therap* or service*)).ti.  or (("scaling" or widespread or spread$ or spreading or "rolling out" or "roll out" or "rolls out" or "rolled out" or "scale$ up" or "scale$ out" or upscaling or scalability or scalable) adj5 (innovation$ or intervention$ or technolog* or practice* or care or initiative* or program* or product* or therap* or service*)).ab.  or (("scaling" or widespread or spread$ or spreading or "rolling out" or "roll out" or "rolls out" or "rolled out" or "scale$ up" or "scale$ out" or upscaling or scalability or scalable) adj5 (innovation$ or intervention$ or technolog* or practice* or care or initiative* or program* or product* or therap* or service*)).hw. | #3 | 4 988 |
|  | ((bring* or brought or taking or take* or increas* or going or implement* or econom*) adj5 scal* adj5 (innovation$ or intervention$ or technolog* or practice* or care or initiative* or program* or product* or therap* or service*)).ti.  or ((bring* or brought or taking or take* or increas* or going or implement* or econom*) adj5 scal* adj5 (innovation$ or intervention$ or technolog* or practice* or care or initiative* or program* or product* or therap* or service*)).ab.  or ((bring* or brought or taking or take* or increas* or going or implement* or econom*) adj5 scal* adj5 (innovation$ or intervention$ or technolog* or practice* or care or initiative* or program* or product* or therap* or service*)).hw. | #4 | 790 |
|  | ("reverse innovation*" or "trickle-up innovation*").ti. or ("reverse innovation*" or "trickle-up innovation*").ab. or ("reverse innovation*" or "trickle-up innovation*").hw. | #5 | 6 |
|  | (transfer* adj5 (innovation$ or intervention$ or technolog* or initiative*)).ti. or (transfer* adj5 (innovation$ or intervention$ or technolog* or initiative*)).ab. or (transfer* adj5 (innovation$ or intervention$ or technolog* or initiative*)).hw. | #6 | 1 451 |
|  | 3 or 4 or 5 or 6 | #7 | 7 103 |
| Scaling | 2 or 7 | #8 | 7 103 |
| Implementation (Controlled vocabulary) | exp continuing education/ | #9 | 4 665 |
| Implementation  (Free text) | (("knowledge to action" or "continuing education" or "know-do" or implement* or dissemin* or diffus* or adopt* or adapt*) adj5 (research or science or bioscience or biomedic* or innovation* or intervention* or technolog* or practice* or care or initiative* or program* or product* or therap* or service*)).ti.  or (("knowledge to action" or "continuing education" or "know-do" or implement* or dissemin* or diffus* or adopt* or adapt*) adj5 (research or science or bioscience or biomedic* or innovation* or intervention* or technolog* or practice* or care or initiative* or program* or product* or therap* or service*)).ab.  or (("knowledge to action" or "continuing education" or "know-do" or implement* or dissemin* or diffus* or adopt* or adapt*) adj5 (research or science or bioscience or biomedic* or innovation* or intervention* or technolog* or practice* or care or initiative* or program* or product* or therap* or service*)).kf. | #10 | 91 976 |
|  | ((applied or mediation* or translat*) adj5 (research or science or bioscience or biomedic*)).ti.  or ((applied or mediation* or translat*) adj5 (research or science or bioscience or biomedic*)).ab.  or ((applied or mediation* or translat*) adj5 (research or science or bioscience or biomedic*)).kf | #11 | 15 637 |
|  | ((mediation* or translat* or populari#ation or populari#e or populari#ed or populari#ing) adj5 (innovation* or intervention* or technolog* or practice* or care or initiative* or program* or product* or therap* or service*)).ti.  or ((mediation* or translat* or populari#ation or populari#e or populari#ed or populari#ing) adj5 (innovation* or intervention* or technolog* or practice* or care or initiative* or program* or product* or therap* or service*)).ab.  or ((mediation* or translat* or populari#ation or populari#e or populari#ed or populari#ing) adj5 (innovation* or intervention* or technolog* or practice* or care or initiative* or program* or product* or therap* or service*)).kf. | #12 | 8 219 |
|  | (knowledge adj3 (transfer* or translat* or broker* or mobil* or uptake or "up take" or adapt* or dispers* or exchange* or application or utili#ation or utili#e or utili#ed or utili#ing or communicat* or cycle? or transform* or action? or manage*) adj5 (innovation* or intervention* or technolog* or practice* or care or initiative* or program* or product* or therap* or service*)).ti.  or (knowledge adj3 (transfer* or translat* or broker* or mobil* or uptake or "up take" or adapt* or dispers* or exchange* or application or utili#ation or utili#e or utili#ed or utili#ing or communicat* or cycle? or transform* or action? or manage*) adj5 (innovation* or intervention* or technolog* or practice* or care or initiative* or program* or product* or therap* or service*)).ab.  or (knowledge adj3 (transfer* or translat* or broker* or mobil* or uptake or "up take" or adapt* or dispers* or exchange* or application or utili#ation or utili#e or utili#ed or utili#ing or communicat* or cycle? or transform* or action? or manage*) adj5 (innovation* or intervention* or technolog* or practice* or care or initiative* or program* or product* or therap* or service*)).hw. | #13 | 3 808 |
| Implementation (Free text) | 10 or 11 or 12 or 13 | #14 | 114 727 |
| Implementation | 9 or 14 | #15 | 118 744 |
| Implementation or scaling up | 8 or 15 | #16 | 123 823 |
| Total result | 1 and 16 | #17 | 1 175 |

### Cochrane Library (2019-05-09)

| **Concepts** | **Search strategy keywords** | **Search** | **# Results** |
| --- | --- | --- | --- |
| Reporting standard (Controlled vocabulary) | Not available | NA | - |
| Reporting standard (Free text) | (((((good or best) NEAR/1 (practice* or practise*)) or guide or guides or guideline* or checklist* or "check list*" or framework* or standard* or recommend* or guidance* or requirement* or instruct* or consensus or "aide memoir*" or quality or "worked example*" or criteri* or critique* or design or designs or clarity or statement* or parameter* or advice* or policy or policies or accura* or appropriate* or "minimum information" or tool* or complete or protocol* or advice* or uniform* or better or strengthen* or transparen*) NEAR/1 (report* or writ*)) or "research standard*" or "publishing standard*"):ti,ab,kw | #1 | 4 870 |
| Scaling (Controlled vocabulary) | Not available | NA | - |
| Scaling (Free text) | (("scaling" or widespread or spread or spreads or spreading or "rolling out" or "roll out" or "rolls out" or "rolled out" or "scale up" or "scaled up" or "scale out" or "scaled out" or upscaling or scalability or scalable) NEAR/4 (innovation or innovations or intervention or interventions or technolog* or practice* or care or initiative* or program* or product* or therap* or service*)):ti,ab,kw | #2 | 2 002 |
|  | ((bring* or brought or taking or take* or increas* or going or implement* or econom*) NEAR/4 (scal*) NEAR/4 (innovation or innovations or intervention or interventions or technolog* or practice* or care or initiative* or program* or product* or therap* or service*)):ti,ab,kw | #3 | 236 |
|  | ("reverse innovation*" or "trickle-up innovation*"):ti,ab,kw | #4 | 2 |
|  | ((transfer*) NEAR/4 (innovation or innovations or intervention or interventions or technolog* or initiative*)):ti,ab,kw | #5 | 478 |
|  | #2 or #3 or #4 or #5 | #6 | 2 673 |
| Implementation (Controlled vocabulary) | MeSH descriptor: [Translational Medical Research] explode all trees | #7 | 115 |
|  | MeSH descriptor: [Information Dissemination] explode all trees | #8 | 216 |
|  | MeSH descriptor: [Diffusion of Innovation] explode all trees | #9 | 144 |
|  | MeSH descriptor: [Knowledge Management] explode all trees | #10 | 3 |
|  | MeSH descriptor: [Education, Continuing] explode all trees | #11 | 1 119 |
|  | MeSH descriptor: [Health Plan Implementation] explode all trees | #12 | 147 |
|  | #7 or #8 or #9 or #10 or #11 or #12 | #13 | 1 690 |
| Implementation  (Free text) | (("knowledge to action" or "continuing education" or "know-do" or implement* or dissemin* or diffus* or adopt* or adapt*) NEAR/4 (research or science or bioscience or biomedic* or innovation* or intervention* or technolog* or practice* or care or initiative* or program* or product* or therap* or service*)):ti,ab,kw | #14 | 19 124 |
|  | ((applied or mediation* or translat*) NEAR/4 (research or science or bioscience or biomedic*)):ti,ab,kw | #15 | 1 851 |
|  | ((mediation* or translat* or popularisation or popularization or popularise or popularize or popularised or popularized or popularising or popularizing) NEAR/4 (innovation* or intervention* or technolog* or practice* or care or initiative* or program* or product* or therap* or service*)):ti,ab,kw | #16 | 1 708 |
|  | (((knowledge) NEAR/2 (transfer* or translat* or broker* or mobil* or uptake or "up take" or adapt* or dispers* or exchange* or application or utilisation or utilization or utilise or utilize or utilised or utilized or utilising or utilizing or communicat* or cycle or cycles or transform* or action or actions or manage*)) NEAR/4 (innovation* or intervention* or technolog* or practice* or care or initiative* or program* or product* or therap* or service*)):ti,ab,kw | #17 | 285 |
| Implementation (Free text) | #14 or #15 or #16 or #17 | #18 | 22 025 |
| Implementation | #13 or #18 | #19 | 23 184 |
| Implementation or scaling up | #6 or #19 | #20 | 25 217 |
| Total result | #1 and #20 | #21 | 270 |

### Cinahl EBSCOhost (2019-05-09)

| **Concepts** | **Search strategy keywords** | **Search** | **# Results** |
| --- | --- | --- | --- |
| Reporting standard (Controlled vocabulary) | (MH "Writing+/ST") OR (MH "Report Writing/ST") or (MH "Writing for Publication/ST") | #1 | 921 |
| Reporting standard (Free text) | TI ( ((((good or best) N1 (practi?e*)) or guide or guides or guideline* or checklist* or "check list*" or framework* or standard* or recommend* or guidance* or requirement* or instruct* or consensus or "aide memoir*" or quality or "worked example*" or criteri* or critique* or design or designs or clarity or statement* or parameter* or advice* or policy or policies or accura* or appropriate* or "minimum information" or tool* or complete or protocol* or advice* or uniform* or better or strengthen* or transparen*) N1 (report* or writ*)) or "research standard*" or "publishing standard*" )  OR AB ( ((((good or best) N1 (practi?e*)) or guide or guides or guideline* or checklist* or "check list*" or framework* or standard* or recommend* or guidance* or requirement* or instruct* or consensus or "aide memoir*" or quality or "worked example*" or criteri* or critique* or design or designs or clarity or statement* or parameter* or advice* or policy or policies or accura* or appropriate* or "minimum information" or tool* or complete or protocol* or advice* or uniform* or better or strengthen* or transparen*) N1 (report* or writ*)) or "research standard*" or "publishing standard*" )  OR SU ( ((((good or best) N1 (practi?e*)) or guide or guides or guideline* or checklist* or "check list*" or framework* or standard* or recommend* or guidance* or requirement* or instruct* or consensus or "aide memoir*" or quality or "worked example*" or criteri* or critique* or design or designs or clarity or statement* or parameter* or advice* or policy or policies or accura* or appropriate* or "minimum information" or tool* or complete or protocol* or advice* or uniform* or better or strengthen* or transparen*) N1 (report* or writ*)) or "research standard*" or "publishing standard*" ) | #2 | 26 778 |
| Reporting standard | S1 or S2 | #3 | 27 187 |
| Scaling (Controlled vocabulary) | (MH "Transferability") | #4 | 454 |
| Scaling (Free text) | TI ( (("scaling" or widespread or spread# or spreading or "rolling out" or "roll out" or "rolls out" or "rolled out" or "scale# up" or "scale# out" or upscaling or scalability or scalable) N4 (innovation# or intervention# or technolog* or practice* or care or initiative* or program* or product* or therap* or service*)) )  OR AB ( (("scaling" or widespread or spread# or spreading or "rolling out" or "roll out" or "rolls out" or "rolled out" or "scale# up" or "scale# out" or upscaling or scalability or scalable) N4 (innovation# or intervention# or technolog* or practice* or care or initiative* or program* or product* or therap* or service*)) )  OR SU ( (("scaling" or widespread or spread# or spreading or "rolling out" or "roll out" or "rolls out" or "rolled out" or "scale# up" or "scale# out" or upscaling or scalability or scalable) N4 (innovation# or intervention# or technolog* or practice* or care or initiative* or program* or product* or therap* or service*)) ) | #5 | 5 760 |
|  | TI ( ((bring* or brought or taking or take* or increas* or going or implement* or econom*) N4 (scal*)) N4 (innovation# or intervention# or technolog* or practice* or care or initiative* or program* or product* or therap* or service*) )  OR AB ( ((bring* or brought or taking or take* or increas* or going or implement* or econom*) N4 (scal*)) N4 (innovation# or intervention# or technolog* or practice* or care or initiative* or program* or product* or therap* or service*) )  OR SU ( ((bring* or brought or taking or take* or increas* or going or implement* or econom*) N4 (scal*)) N4 (innovation# or intervention# or technolog* or practice* or care or initiative* or program* or product* or therap* or service*) ) | #6 | 768 |
|  | TI ( "reverse innovation*" or "trickle-up innovation*" )  OR AB ( "reverse innovation*" or "trickle-up innovation*" )  OR SU ( "reverse innovation*" or "trickle-up innovation*" ) | #7 | 20 |
|  | TI ((transfer*) N4 (innovation# or intervention# or technolog* or initiative*) ) OR AB ( (transfer*) N4 (innovation# or intervention# or technolog* or initiative*) )  OR SU ( (transfer*) N4 (innovation# or intervention# or technolog* or initiative*) ) | #8 | 1 103 |
|  | S5 or S6 or S7 or S8 | #9 | 7 466 |
| Scaling | S4 or S9 | #10 | 7 913 |
| Implementation (Controlled vocabulary) | (MH "Diffusion of Innovation+") or (MH "Selective Dissemination of Information") or (MH "Knowledge Management") or (MH "Education, Continuing+") or (MH "Program Implementation") or (MH "Applied Research") or (MH "Health Care Information Exchange (Iowa NIC)") | #11 | 65 676 |
| Implementation  (Free text) | TI ( ("knowledge to action" or "continuing education" or "know-do" or implement* or dissemin* or diffus* or adopt* or adapt*) N4 (research or science or bioscience or biomedic* or innovation* or intervention* or technolog* or practice* or care or initiative* or program* or product* or therap* or service*) )  OR AB ( ("knowledge to action" or "continuing education" or "know-do" or implement* or dissemin* or diffus* or adopt* or adapt*) N4 (research or science or bioscience or biomedic* or innovation* or intervention* or technolog* or practice* or care or initiative* or program* or product* or therap* or service*) )  OR SU ( ("knowledge to action" or "continuing education" or "know-do" or implement* or dissemin* or diffus* or adopt* or adapt*) N4 (research or science or bioscience or biomedic* or innovation* or intervention* or technolog* or practice* or care or initiative* or program* or product* or therap* or service*) ) | #12 | 108 660 |
|  | TI ( (applied or mediation* or translat*) N4 (research or science or bioscience or biomedic*) )  OR AB ( (applied or mediation* or translat*) N4 (research or science or bioscience or biomedic*) )  OR SU ( (applied or mediation* or translat*) N4 (research or science or bioscience or biomedic*) ) | #13 | 9 349 |
|  | TI ( (mediation* or translat* or populari#ation or populari#e or populari#ed or populari#ing) N4 (innovation* or intervention* or technolog* or practice* or care or initiative* or program* or product* or therap* or service*) )  OR AB ( (mediation* or translat* or populari#ation or populari#e or populari#ed or populari#ing) N4 (innovation* or intervention* or technolog* or practice* or care or initiative* or program* or product* or therap* or service*) )  OR SU ( (mediation* or translat* or populari#ation or populari#e or populari#ed or populari#ing) N4 (innovation* or intervention* or technolog* or practice* or care or initiative* or program* or product* or therap* or service*) ) | #14 | 7 845 |
|  | TI ( ((knowledge) N2 (transfer* or translat* or broker* or mobil* or uptake or "up take" or adapt* or dispers* or exchange* or application or utili#ation or utili#e or utili#ed or utili#ing or communicat* or cycle# or transform* or action# or manage*)) N4 (innovation* or intervention* or technolog* or practice* or care or initiative* or program* or product* or therap* or service*) )  OR AB ( ((knowledge) N2 (transfer* or translat* or broker* or mobil* or uptake or "up take" or adapt* or dispers* or exchange* or application or utili#ation or utili#e or utili#ed or utili#ing or communicat* or cycle# or transform* or action# or manage*)) N4 (innovation* or intervention* or technolog* or practice* or care or initiative* or program* or product* or therap* or service*) )  OR SU ( ((knowledge) N2 (transfer* or translat* or broker* or mobil* or uptake or "up take" or adapt* or dispers* or exchange* or application or utili#ation or utili#e or utili#ed or utili#ing or communicat* or cycle# or transform* or action# or manage*)) N4 (innovation* or intervention* or technolog* or practice* or care or initiative* or program* or product* or therap* or service*) ) | #15 | 2 660 |
| Implementation (Free text) | S12 or S13 or S14 or S15 | #16 | 123 016 |
| Implementation | S11 or S16 | #17 | 152 865 |
| Implementation or scaling up | S10 or S17 | #18 | 158 614 |
| Total result | S3 and S18 | #19 | 1 875 |

### Web of Science (2019-05-09)

| **Concepts** | **Search strategy keywords** | **Search** | **# Results** |
| --- | --- | --- | --- |
| Reporting standard (Free text) | TS=(((((good or best) NEAR/1 (practi?e*)) or guide or guides or guideline* or checklist* or "check list*" or framework* or standard* or recommend* or guidance* or requirement* or instruct* or consensus or "aide memoir*" or quality or "worked example*" or criteri* or critique* or design or designs or clarity or statement* or parameter* or advice* or policy or policies or accura* or appropriate* or "minimum information" or tool* or complete or protocol* or advice* or uniform* or better or strengthen* or transparen*) NEAR/1 (report* or writ*)) or "research standard*" or "publishing standard*") | #1 | 114 316 |
| Scaling (Free text) | TS=(("scaling" or widespread or spread$ or spreading or "rolling out" or "roll out" or "rolls out" or "rolled out" or "scale$ up" or "scale$ out" or upscaling or scalability or scalable) NEAR/4 (innovation$ or intervention$ or technolog* or practice* or care or initiative* or program* or product* or therap* or service*)) | #2 | 47 316 |
|  | TS=(((bring* or brought or taking or take* or increas* or going or implement* or econom*) NEAR/4 (scal*)) NEAR/4 (innovation$ or intervention$ or technolog* or practice* or care or initiative* or program* or product* or therap* or service*)) | #3 | 7 080 |
|  | TS=("reverse innovation*" or "trickle-up innovation*") | #4 | 130 |
|  | TS=((transfer*) NEAR/4 (innovation$ or intervention$ or technolog* or initiative*)) | #5 | 23 249 |
|  | #2 or #3 or #4 or #5 | #6 | 76 186 |
| Implementation  (Free text) | TS=(("knowledge to action" or "continuing education" or "know-do" or implement* or dissemin* or diffus* or adopt* or adapt*) NEAR/4 (research or science or bioscience or biomedic* or innovation* or intervention* or technolog* or practice* or care or initiative* or program* or product* or therap* or service*)) | #7 | 402 904 |
|  | TS=((applied or mediation* or translat*) NEAR/4 (research or science or bioscience or biomedic*)) | #8 | 77 956 |
|  | TS=((mediation* or translat* or populari?ation or populari?e or populari?ed or populari?ing) NEAR/4 (innovation* or intervention* or technolog* or practice* or care or initiative* or program* or product* or therap* or service*)) | #9 | 37 131 |
|  | TS=(((knowledge) NEAR/2 (transfer* or translat* or broker* or mobil* or uptake or "up take" or adapt* or dispers* or exchange* or application or utili?ation or utili?e or utili?ed or utili?ing or communicat* or cycle? or transform* or action? or manage*)) NEAR/4 (innovation* or intervention* or technolog* or practice* or care or initiative* or program* or product* or therap* or service*)) | #10 | 14 056 |
| Implementation (Free text) | #7 or #8 or #9 or #10 | #11 | 516 194 |
| Implementation or scaling up | #6 or #11 | #12 | 580 373 |
| Total result | #1 and #12 | #13 | 4 346 |
| Health (Free text) | TS=(Health* or medicin* or medica* or clinic* or therap* or disease* or illness or diagnos* or treatment* or prevention* or drug* or remed* or pharmaceutic* or pill* or pharma* or healing* or surger* or infection* or patholog* or wound* or injur*) | #14 | 16 118 238 |
| Total result | #13 AND #14 | #15 | 2 906 |
