# Supplementary material for: Informing the development of the SUCCEED reporting guideline for studies on the scaling of health interventions: A systematic review
Source: Medicine (Baltimore). 2024 Feb 16;103(7):e37079. doi: 10.1097/MD.0000000000037079 (PMC10869056; doi:10.1097/MD.0000000000037079)
Supplement: Supplementary file 2 [file medi-103-e37079-s002.docx]

### Grey literature

| **Name of Organization** | **Url** | **Date** |
| --- | --- | --- |
| EQUATOR Network | <http://www.equator-network.org> | October 3, 2019 |
| WHO/ExpandNet | <http://expandnet.net/biblio/> | October 4, 2019 |
|  | <https://www.globalreporting.org/Pages/resource-library.aspx> |  |
| International Development Research Centre | <https://idl-bnc-idrc.dspacedirect.org/> | October 9, 2019 |
| NSW Government | <https://www.health.nsw.gov.au/Pages/default.aspx> | October 10, 2019 |
| Canadian Foundation for Healthcare Improvement (CFHI) | <https://www.cfhi-fcass.ca/> | October 11, 2019 |
| Global Reporting Initiative | <https://www.globalreporting.org/Pages/resource-library.aspx> | October 11, 2019 |
| AcademyHealth | <https://www.academyhealth.org/> | October 23, 2019 |
| The Evidence Project | <http://evidenceproject.popcouncil.org/resources/> | October 23, 2019 |
| The Health Foundation | <https://www.health.org.uk/search> | October 23, 2019 |
| European Implementation Collaborative | <https://implementation.eu/> | October 24, 2019 |
| Dissemination and Implementation Models | <http://dissemination-implementation.org/index.aspx> | October 24, 2019 |
| Comet register | <http://www.comet-initiative.org/studies/search> | October 25, 2019 |
| The World Bank | <https://bit.ly/3fxwoLe> | October 25, 2019 |
| The Wallace Foundation | <https://www.wallacefoundation.org/pages/default.aspx> | October 25, 2019 |
| Bill & Melinda Gates Foundation | [https://www.gatesfoundation.org/search#](https://www.gatesfoundation.org/search) | October 29, 2019 |
| Scaling Pathways | <http://scalingpathways.globalinnovationexchange.org/resources> | October 29, 2019 |
| UNICEF | <https://www.unicef.org/> | October 29, 2019 |
| What Works Clearinghouse | <https://ies.ed.gov/pubsearch/> | October 30, 2019 |
| Canadian agency for Drugs and Technologies in Health | <https://bit.ly/3U4FEFw> | October 30, 2019 |
| National Institute for Health and Care Excellence | <https://www.nice.org.uk/search?q=> | October 30, 2019 |
| Google Search* | [https://www.google.com/search?q=%s&pws=0](https://www.google.com/search?q=%25s&pws=0) | October 31, 2019 |
| Grand Challenges Canada | <https://bit.ly/3fy3smq> | December 6, 2019 |

### Searched terms included: scaling up, scaling out, spread, scale up, scale out, upscaling, scalability, dissemination, diffusion, implementation

***** We first anonymized the Google search using the following URL: [https://www.google.com/search?q=%s&pws=0](https://www.google.com/search?q=%25s&pws=0) and then applied the PDF filter; we retained the first 200 hits.
