# Supplementary material for: Informing the development of the SUCCEED reporting guideline for studies on the scaling of health interventions: A systematic review
Source: Medicine (Baltimore). 2024 Feb 16;103(7):e37079. doi: 10.1097/MD.0000000000037079 (PMC10869056; doi:10.1097/MD.0000000000037079)
Supplement: Supplementary file 3 [file medi-103-e37079-s003.docx]

**Informing the development of the SUCCEED reporting guideline for on the scaling of studies of health interventions: a systematic review**

Gogovor et al.

| **Authors** | **Published Year** | **Reason for exclusion** | **Full reference** |
| --- | --- | --- | --- |
| Chan et al. | 2006 | not a reporting guideline | Chan, Charles C.; Chan, Kevin.Programs Effectiveness, Process Outcomes, and Sustainability of Health Promotion Interventions in Hong Kong: Applying the RE-AIM Framework.Journal of Psychology in Chinese Societies.7:1.5-27.2006. |
| Virani et al. | 1994 | not a reporting guideline | Virani, T.; Shamian, J..Development and monitoring of nursing standards.Nover.7:2.5-9.1994. |
| Schwartz et al. | 2009 | not a reporting guideline | Schwartz, J..AAC-RERC spread the word. Announcing: the free AAC-RERC Writers Brigade Guide.Augmentative Communication News.21:1.4-4.2009. |
| Staniszewska et al. | 2017 | not a guideline for reporting implementation or scaling | Staniszewska, S.; Brett, J.; Simera, I.; Seers, K.; Mockford, C.; Goodlad, S.; Altman, D. G.; Moher, D.; Barber, R.; Denegri, S.; Entwistle, A.; Littlejohns, P.; Morris, C.; Suleman, R.; Thomas, V.; Tysall, C..GRIPP2 reporting checklists: tools to improve reporting of patient and public involvement in research.BMJ.358:.j3453.2017.10.1136/bmj.j3453 |
| Pinnock et al. | 2015 | duplicate or secondary reporting of the guideline | Pinnock, H.; Epiphaniou, E.; Sheikh, A.; Griffiths, C.; Eldridge, S.; Craig, P.; Taylor, S. J..Developing standards for reporting implementation studies of complex interventions (StaRI): a systematic review and e-Delphi.Implementation Science.10:.42.2015.10.1186/s13012-015-0235-z |
| Pinnock et al. | 2017 | duplicate or secondary reporting of the guideline | Pinnock, H.; Barwick, M.; Carpenter, C. R.; Eldridge, S.; Grandes, G.; Griffiths, C. J.; Rycroft-Malone, J.; Meissner, P.; Murray, E.; Patel, A.; Sheikh, A.; Taylor, S. J.; Sta, R. I. Group.Standards for Reporting Implementation Studies (StaRI): explanation and elaboration document.BMJ Open.7:4.e013318.2017.10.1136/bmjopen-2016-013318 |
| Jeswani et al. | 2015 | not a reporting guideline | Jeswani, N.; Gaur, P.; Ganesan, N.; Mitchell, K..A conceptual framework for translating patient-reported outcomes for implementation in clinical practice and quality improvement efforts.Value in Health.18:3.A111-A112.2015. |
| Hooker et al. | 2017 | not a guideline for reporting implementation or scaling | Hooker, G. W.; Babu, D.; Myers, M. F.; Zierhut, H.; McAllister, M..Standards for the Reporting of Genetic Counseling Interventions in Research and Other Studies (GCIRS): an NSGC Task Force Report.Journal of Genetic Counseling.26:3.355-360.2017.10.1007/s10897-017-0076-9 |
| Butcher et al. | 2019 | not a guideline for reporting implementation or scaling | Butcher, N. J.; Monsour, A.; Mew, E. J.; Szatmari, P.; Pierro, A.; Kelly, L. E.; Farid-Kapadia, M.; Chee, A. Tow A.; Saeed, L.; Monga, S.; Ungar, W.; Terwee, C. B.; Vohra, S.; Fergusson, D.; Askie, L. M.; Williamson, P. R.; Chan, A. W.; Moher, D.; Offringa, M..Improving outcome reporting in clinical trial reports and protocols: study protocol for the Instrument for reporting Planned Endpoints in Clinical Trials (InsPECT).Trials [Electronic Resource].20:1.161.2019.10.1186/s13063-019-3248-0 |
| Bragge et al. | 2017 | not a reporting guideline | Bragge, P.; Grimshaw, J. M.; Lokker, C.; Colquhoun, H.; Aimd Writing/Working Group.AIMD - a validated, simplified framework of interventions to promote and integrate evidence into health practices, systems, and policies.BMC Medical Research Methodology.17:1.38.2017.10.1186/s12874-017-0314-8 |
| AmatoNeto et al. | 2002 | not a reporting guideline | Amato Neto, V..[Dissemination of research results and knowledge through scientific journals].Revista Da Sociedade Brasileira de Medicina Tropical.35:2.197-8.2002. |
| Abraham et al. | 2014 | not a guideline for reporting implementation or scaling | Abraham, C.; Johnson, B. T.; de Bruin, M.; Luszczynska, A..Enhancing reporting of behavior change intervention evaluations.Journal of Acquired Immune Deficiency Syndromes: JAIDS.66 Suppl 3:Suppl 3.S293-9.2014.10.1097/QAI.0000000000000231 |
| Tournoux et al. | 2009 | duplicate or secondary reporting of the guideline | Tournoux, C.; Brindel, P.; Jais, J. P.; Landais, P..[Understanding publication standards for evidence-based medicine].Presse Medicale.38:4.591-6.2009.10.1016/j.lpm.2008.05.021 |
| Onwuegbuzie et al. | 2014 | not a guideline for reporting implementation or scaling | Onwuegbuzie, Anthony J.; Corrigan, Julie A..Improving the Quality of Mixed Research Reports in the Field of Human Resource Development and Beyond: A Call for Rigor as an Ethical Practice.Human Resource Development Quarterly.25:3.273-299.2014.10.1002/hrdq.21197 |
| Leeman et al. | 2017 | not a reporting guideline | Leeman, J.; Birken, S. A.; Powell, B. J.; Rohweder, C.; Shea, C. M..Beyond "implementation strategies": classifying the full range of strategies used in implementation science and practice.Implementation Science.12:1.125.2017.10.1186/s13012-017-0657-x |
| Kumpfer et al. | 2018 | not a guideline for reporting implementation or scaling | Kumpfer, K. L.; Scheier, L. M.; Brown, J..Strategies to Avoid Replication Failure With Evidence-Based Prevention Interventions: Case Examples From the Strengthening Families Program.Evaluation & the Health Professions.:.163278718772886.2018.10.1177/0163278718772886 |
| Grant et al. | 2013 | not a guideline for reporting implementation or scaling | Grant, S. P.; Mayo-Wilson, E.; Melendez-Torres, G. J.; Montgomery, P..Reporting quality of social and psychological intervention trials: a systematic review of reporting guidelines and trial publications.PLoS ONE [Electronic Resource].8:5.e65442.2013.10.1371/journal.pone.0065442 |
| Grandes et al. | 2018 | duplicate or secondary reporting of the guideline | Grandes, G.; Pinnock, H.; Bazemore, A.; Meissner, P.; Sta, R. I. Group.Improving the Quality of Primary Care by Optimizing Implementation Research Reporting.Journal of the American Board of Family Medicine: JABFM.31:3.484-487.2018.10.3122/jabfm.2018.03.170195 |
| Gagnier et al. | 2013 | not a guideline for reporting implementation or scaling | Gagnier, J. J.; Riley, D.; Altman, D. G.; Moher, D.; Sox, H.; Kienle, G.; Care Group.The CARE guidelines: consensus-based clinical case reporting guideline development..110:37.603-8.2013.10.3238/arztebl.2013.0603 |
| Gagnier et al. | 2013 | not a guideline for reporting implementation or scaling | Gagnier, J. J.; Kienle, G.; Altman, D. G.; Moher, D.; Sox, H.; Riley, D.; Care Group.The CARE guidelines: consensus-based clinical case report guideline development.Journal of Dietary Supplements.10:4.381-90.2013.10.3109/19390211.2013.830679 |
| Gagnier et al. | 2014 | not a guideline for reporting implementation or scaling | Gagnier, J. J.; Kienle, G.; Altman, D. G.; Moher, D.; Sox, H.; Riley, D.; Care Group.The CARE guidelines: consensus-based clinical case report guideline development.Journal of Clinical Epidemiology.67:1.46-51.2014.10.1016/j.jclinepi.2013.08.003 |
| Gagnier et al. | 2013 | not a guideline for reporting implementation or scaling | Gagnier, J. J.; Kienle, G.; Altman, D. G.; Moher, D.; Sox, H.; Riley, D.; Care Group*.The CARE Guidelines: Consensus-based Clinical Case Reporting Guideline Development..2:5.38-43.2013.10.7453/gahmj.2013.008 |
| Fain et al. | 2016 | duplicate or secondary reporting of the guideline | Fain, J. A..TDE Adopts the Standards for Quality Improvement Reporting Excellence (SQUIRE) Guidelines.Diabetes Educator.42:3.269-70.2016.10.1177/0145721716646297 |
| Estabrooks et al. | 2018 | not a reporting guideline | Estabrooks, P. A.; Brownson, R. C.; Pronk, N. P..Dissemination and Implementation Science for Public Health Professionals: An Overview and Call to Action.Preventing Chronic Disease.15:.E162.2018.10.5888/pcd15.180525 |
| Ellis et al. | 2005 | not a reporting guideline | Ellis, I.; Howard, P.; Larson, A.; Robertson, J..From workshop to work practice: An exploration of context and facilitation in the development of evidence-based practice.Worldviews on Evidence-Based Nursing.2:2.84-93.2005.10.1111/j.1741-6787.2005.04088.x |
| Doshi et al. | 2012 | not a reporting guideline | Doshi, P.; Jefferson, T.; Del Mar, C..The imperative to share clinical study reports: recommendations from the Tamiflu experience.PLoS Medicine / Public Library of Science.9:4.e1001201.2012.10.1371/journal.pmed.1001201 |
| DeGeest et al. | 2018 | not a reporting guideline | De Geest, S.; Zullig, L. L.; Dunbar-Jacob, J.; Helmy, R.; Hughes, D. A.; Wilson, I. B.; Vrijens, B..ESPACOMP Medication Adherence Reporting Guideline (EMERGE).Annals of Internal Medicine.169:1.30-35.2018.10.7326/M18-0543 |
| deClercq et al. | 2004 | not a reporting guideline | de Clercq, P. A.; Blom, J. A.; Korsten, H. H.; Hasman, A..Approaches for creating computer-interpretable guidelines that facilitate decision support.Artificial Intelligence in Medicine.31:1.1-27.2004.10.1016/j.artmed.2004.02.003 |
| Cotterill et al. | 2018 | duplicate or secondary reporting of the guideline | Cotterill, S.; Knowles, S.; Martindale, A. M.; Elvey, R.; Howard, S.; Coupe, N.; Wilson, P.; Spence, M..Getting messier with TIDieR: embracing context and complexity in intervention reporting.BMC Medical Research Methodology.18:1.12.2018.10.1186/s12874-017-0461-y |
| Bigby et al. | 2010 | not a reporting guideline | Bigby, Christine.Rankings, Ratings, and Reviews.Australian Social Work.63:4.371-374.2010.10.1080/0312407x.2010.524142 |
| Berry et al. | 2018 | not a reporting guideline | Berry, W. R.; Edmondson, L.; Gibbons, L. R.; Childers, A. K.; Haynes, A. B.; Foster, R.; Singer, S. J.; Gawande, A. A..Scaling Safety: The South Carolina Surgical Safety Checklist Experience.Health Affairs.37:11.1779-1786.2018.10.1377/hlthaff.2018.0717 |
| Becker et al. | 2005 | not a reporting guideline | Becker, G. J..How HHS plans to move medical innovations forward: summary of task force report and recommendations..2:6.468-70.2005.10.1016/j.jacr.2005.02.017 |
| Barber et al. | 2012 | not a reporting guideline | Barber, A..Computers for physicians: never do harm.Care Management Journals.13:4.194-9.2012. |
| Babu et al. | 2013 | not a reporting guideline | Babu, A..Emergent areas to visualize by the journal strategy holders.Journal of Contemporary Dental Practice [Electronic Resource].14:6.i-ii.2013. |
| Adjemian et al. | 2017 | not a reporting guideline | Adjemian, Raffi; Moradi Zirkohi, Atbin; Coombs, Robin; Mickan, Sharon; Vaillancourt, Christian.Are emergency department clinical pathway interventions adequately described, and are they delivered as intended? A systematic review.International Journal of Care Coordination.20:4.148-161.2017.10.1177/2053434517732507 |
| Aboud et al. | 2018 | not a reporting guideline | Aboud, F. E.; Yousafzai, A. K.; Nores, M..State of the science on implementation research in early child development and future directions.Annals of the New York Academy of Sciences.1419:1.264-271.2018.10.1111/nyas.13722 |
| Solomon et al. | 2009 | not a guideline for reporting implementation or scaling | Solomon, Phyllis; Cavanaugh, Mary M.; Draine, Jeffrey.Randomized Controlled Trials..:..2009.10.1093/acprof:oso/9780195333190.001.0001 |
| Shahian et al. | 2017 | not a reporting guideline | Shahian, D. M.; McEachern, K.; Rossi, L.; Chisari, R. G.; Mort, E..Large-scale implementation of the I-PASS handover system at an academic medical centre.BMJ Quality & Safety.26:9.760-770.2017.10.1136/bmjqs-2016-006195 |
| Sandrawati et al. | 2014 | not a reporting guideline | Sandrawati, J.; Supriyanto, S.; Nurul, R. T..Recommendations to Improve the Implementation Compliance of Surgical Safety Checklist in Surgery Rooms.Buletin Penelitian Sistem Kesehatan.17:1.71-79.2014. |
| Sandelowski et al. | 2010 | not a reporting guideline | Sandelowski, M. J..Getting it right.Research in Nursing & Health.33:1.1-3.2010.10.1002/nur.20365 |
| Rosenfield et al. | 2005 | not a reporting guideline | Rosenfield, K.; Babb, J. D.; Cates, C. U.; Cowley, M. J.; Feldman, T.; Gallagher, A.; Gray, W.; Green, R.; Jaff, M. R.; Kent, K. C.; Ouriel, K.; Roubin, G. S.; Weiner, B. H.; White, C. J..Clinical competence statement on carotid stenting: training and credentialing for carotid stenting--multispecialty consensus recommendations: a report of the SCAI/SVMB/SVS Writing Committee to develop a clinical competence statement on carotid interventions.Journal of the American College of Cardiology.45:1.165-74.2005.10.1016/j.jacc.2004.11.016 |
| Roland et al. | 2010 | not a reporting guideline | Roland, S. I..The Physician Quality Reporting Initiative: secrets to success.Family Practice Management.17:1.24-6.2010. |
| Rantz et al. | 2003 | not a reporting guideline | Rantz, M. J.; Vogelsmeier, A.; Manion, P.; Minner, D.; Markway, B.; Conn, V.; Aud, M. A.; Mehr, D. R..Statewide strategy to improve quality of care in nursing facilities.Gerontologist.43:2.248-58.2003.10.1093/geront/43.2.248 |
| Quessy et al. | 2010 | not a reporting guideline | Quessy, S. N..The challenges of translational research for analgesics: the state of knowledge needs upgrading and some uncomfortable deficiencies remain to be urgently addressed.Journal of Pain.11:7.698-700.2010.10.1016/j.jpain.2010.05.001 |
| Purser et al. | 2010 | not a reporting guideline | Purser, L. A.; Barlow-Stewart, K..Translating evidence based research into a tool for consumers.Twin Research and Human Genetics.13:6.658.2010. |
| Proehl et al. | 2017 | not a reporting guideline | Proehl, J. A.; Alexander, S.; Manton, A. P..Integrity and Transparency in Reporting Clinical Trials.CIN: Computers, Informatics, Nursing.35:1.1-2.2017.10.1097/CIN.0000000000000327 |
| Pinnock et al. | 2014 | not a reporting guideline | Pinnock, H.; Epiphaniou, E.; Taylor, S. J..Phase IV implementation studies. The forgotten finale to the complex intervention methodology framework.Annals of the American Thoracic Society.11 Suppl 2:.S118-22.2014.10.1513/AnnalsATS.201308-259RM |
| Pimenta et al. | 2000 | not a reporting guideline | Pimenta, J.; Catchpole, M.; Gray, M.; Hopwood, J.; Randall, S..Evidence based health policy report. Screening for genital chlamydial infection.BMJ.321:7261.629-31.2000.10.1136/bmj.321.7261.629 |
| O'Neil et al. | 2017 | not a reporting guideline | O'Neil, A.; Cocker, F.; Rarau, P.; Baptista, S.; Cassimatis, M.; Barr Taylor, C.; Lau, A. Y. S.; Kanuri, N.; Oldenburg, B..Using digital interventions to improve the cardiometabolic health of populations: a meta-review of reporting quality.Journal of the American Medical Informatics Association.24:4.867-879.2017.10.1093/jamia/ocw166 |
| Moltaji et al. | 2018 | not a reporting guideline | Moltaji, S.; Alkhatib, A. H.; Liu, H.; Murphy, J.; Gallo, L.; Karpinski, M.; Mowakket, S.; Thoma, A..Introducing Knowledge Translation to Plastic Surgery: Turning Evidence into Practice.Plast Reconstr Surg.142:5.760e-769e.2018.10.1097/PRS.0000000000004891 |
| Melby et al. | 2006 | not a reporting guideline | Melby, C. S..Disseminating research internationally.Nursing & Health Sciences.8:1.1.2006.10.1111/j.1442-2018.2006.00268.x |
| McLaren et al. | 2012 | not a reporting guideline | McLaren, P. J..Not window dressing, but key to making medical research matter.Medical Journal of Australia.197:6.328.2012. |
| McDevitt et al. | 2018 | not a reporting guideline | McDevitt, M.; Goodman, D. M.; Balistreri, W. F..The Journal's updated policy on reporting guidelines and data sharing statements.Journal of Pediatrics.199:.1-2.2018.10.1016/j.jpeds.2018.06.023 |
| Mayo-Wilson et al. | 2007 | not a reporting guideline | Mayo-Wilson, E..Reporting implementation in randomized trials: proposed additions to the consolidated standards of reporting trials statement.American Journal of Public Health.97:4.630-3.2007.10.2105/AJPH.2006.094169 |
| Lin et al. | 2011 | not a reporting guideline | Lin, M. C.; Hughes, B. L.; Katica, M. K.; Dining-Zuber, C.; Plsek, P. E..Service Design and Change of Systems: Human-Centered Approaches to Implementing and Spreading Service Design.International Journal of Design.5:2.73-86.2011. |
| Gagnier et al. | 2013 | not a guideline for reporting implementation or scaling | Gagnier, J. J.; Kienle, G.; Altman, D. G.; Moher, D.; Sox, H.; Riley, D.; Care Group.The CARE guidelines: consensus-based clinical case reporting guideline development.BMJ Case Reports.2013:.23.2013.10.1136/bcr-2013-201554 |
| Foster et al. | 2009 | not a guideline for reporting implementation or scaling | Foster, N. E.; Dziedzic, K. S.; van der Windt, D. A.; Fritz, J. M.; Hay, E. M..Research priorities for non-pharmacological therapies for common musculoskeletal problems: nationally and internationally agreed recommendations..10:.3.2009.10.1186/1471-2474-10-3 |
| FerrantediRuffano et al. | 2017 | not a guideline for reporting implementation or scaling | Ferrante di Ruffano, L.; Dinnes, J.; Taylor-Phillips, S.; Davenport, C.; Hyde, C.; Deeks, J. J..Research waste in diagnostic trials: a methods review evaluating the reporting of test-treatment interventions.BMC Medical Research Methodology.17:1.32.2017.10.1186/s12874-016-0286-0 |
| Fernald et al. | 2012 | not a guideline for reporting implementation or scaling | Fernald, D.; Harris, A.; Deaton, E. A.; Weister, V.; Pray, S.; Baumann, C.; Levinson, A..A standardized reporting system for assessment of diverse public health programs.Preventing Chronic Disease.9:.E147.2012.10.5888/pcd9.120004 |
| Dawkins et al. | 2018 | not a reporting guideline | Dawkins, M.; Evans, C. J.; Murtagh, F. E. M..10th World Research Congress of the European Association for Palliative Care (EAPC).Palliative Medicine.32:1_suppl.3-330.2018.10.1177/0269216318769196 |
| Dartnell et al. | 2008 | not a guideline for reporting implementation or scaling | Dartnell, J.; Hemming, M.; Collier, J.; Ollenschlaeger, G..Putting evidence into context: some advice for guideline writers.Evidence Based Nursing.11:1.6-8.2008. |
| Dancey et al. | 2010 | not a reporting guideline | Dancey, J. E..From quality of publication to quality of care: translating trials to practice.Journal of the National Cancer Institute.102:10.670-1.2010.10.1093/jnci/djq142 |
| Crawford et al. | 2018 | not a reporting guideline | Crawford, T. J.; Bay, J.; Brown, J. A..A Critical Appraisal of Maternal and Perinatal Clinical Practice Guidelines.Journal of Paediatrics and Child Health.54:.14-14.2018.10.1111/jpc.13882_30 |
| Cook et al. | 2014 | not a reporting guideline | Cook, C.; Jull, G.; Moore, A..Manual Therapy adopts mandatory reporting guidelines for publishing..19:5.365-6.2014.10.1016/j.math.2014.07.011 |
| Anonymous | 2010 | not a reporting guideline | .Letter from the Editor.Eastern Mediterranean Health Journal.16:12.1203-1203.2010. |
| Zoog et al. | 2011 | not a reporting guideline | Zoog, Holly Brenza; Chang, Ting.Interpretation and Implementation of Good Publication Practice.Drug Information Journal.45:2.137-144.2011.10.1177/009286151104500207 |
| Zhang et al. | 2016 | not a reporting guideline | Zhang, H.; Han, J.; Zhu, Y. B.; Lau, W. Y.; Schwartz, M. E.; Xie, G. Q.; Dai, S. Y.; Shen, Y. N.; Wu, M. C.; Shen, F.; Yang, T..Reporting and methodological qualities of published surgical meta-analyses.Journal of Clinical Epidemiology.70:.4-16.2016.10.1016/j.jclinepi.2015.06.009 |
| Yu et al. | 2018 | not a reporting guideline | Yu, D. D.; Xie, Y. M.; Liao, X.; Zhi, Y. J.; Jiang, J. J.; Chen, W..[Methodological quality and reporting quality evaluation of randomized controlled trials published in China Journal of Chinese Materia Medica].Zhongguo Zhong Yao Za Zhi/Zhongguo Zhongyao Zazhi/China Journal of Chinese Materia Medica.43:4.833-839.2018.10.19540/j.cnki.cjcmm.20171107.003 |
| Yousafzai et al. | 2014 | not a reporting guideline | Yousafzai, A. K.; Aboud, F.; Black, M. M.; Dewey, K. G..Review of implementation processes for integrated nutrition and psychosocial stimulation interventions..1308:.33-45.2014.10.1111/nyas.12313 |
| White et al. | 2019 | not a reporting guideline | White, M. C.; Randall, K.; Capo-Chichi, N. F. E.; Sodogas, F.; Quenum, S.; Wright, K.; Close, K. L.; Russ, S.; Sevdalis, N.; Leather, A. J. M..Implementation and evaluation of nationwide scale-up of the Surgical Safety Checklist.British Journal of Surgery.106:2.e91-e102.2019.10.1002/bjs.11034 |
| Ward et al. | 2013 | not a guideline for reporting implementation or scaling | Ward, L.; Stebbings, S.; Sherman, K.; Cherkin, D.; Baxter, G. D..Standardization of design and reporting of yoga interventions for musculoskeletal conditions: A Delphi survey approach.International Journal of Rheumatic Diseases.16:.114.2013. |
| Veniegas et al. | 2009 | not a reporting guideline | Veniegas, R. C.; Kao, U. H.; Rosales, R..Adapting HIV prevention evidence-based interventions in practice settings: an interview study.Implement Sci.4:.76.2009.10.1186/1748-5908-4-76 |
| vanderWorp et al. | 2012 | not a reporting guideline | van der Worp, H. Bart; Sandercock, Peter A. G..Improving the process of translational research: The application of reporting standards may lead to more useful animal studies.BMJ: British Medical Journal.345:7886.9-9.2012. |
| Ugalde et al. | 2018 | not a reporting guideline | Ugalde, A.; Kiss, N.; Livingston, P.; Aranda, S..Towards a framework for reporting self-guided interventions for people with cancer..12:3.293-298.2018.10.1097/SPC.0000000000000353 |
| Troia et al. | 2013 | not a reporting guideline | Troia, Gary A.; Olinghouse, Natalie G..The and Common Core State Standards and evidence-based educational practices: The case of writing.School Psychology Review.42:3.343-357.2013. |
| Tricco et al. | 2011 | not a reporting guideline | Tricco, A. C.; Tetzlaff, J.; Moher, D..The art and science of knowledge synthesis.Journal of Clinical Epidemiology.64:1.11-20.2011.10.1016/j.jclinepi.2009.11.007 |
| Taylor et al. | 2008 | not a guideline for reporting implementation or scaling | Taylor, C. F.; Field, D.; Sansone, S. A.; Aerts, J.; Apweiler, R.; Ashburner, M.; Ball, C. A.; Binz, P. A.; Bogue, M.; Booth, T.; Brazma, A.; Brinkman, R. R.; Michael Clark, A.; Deutsch, E. W.; Fiehn, O.; Fostel, J.; Ghazal, P.; Gibson, F.; Gray, T.; Grimes, G.; Hancock, J. M.; Hardy, N. W.; Hermjakob, H.; Julian, R. K., Jr.; Kane, M.; Kettner, C.; Kinsinger, C.; Kolker, E.; Kuiper, M.; Le Novere, N.; Leebens-Mack, J.; Lewis, S. E.; Lord, P.; Mallon, A. M.; Marthandan, N.; Masuya, H.; McNally, R.; Mehrle, A.; Morrison, N.; Orchard, S.; Quackenbush, J.; Reecy, J. M.; Robertson, D. G.; Rocca-Serra, P.; Rodriguez, H.; Rosenfelder, H.; Santoyo-Lopez, J.; Scheuermann, R. H.; Schober, D.; Smith, B.; Snape, J.; Stoeckert, C. J., Jr.; Tipton, K.; Sterk, P.; Untergasser, A.; Vandesompele, J.; Wiemann, S..Promoting coherent minimum reporting guidelines for biological and biomedical investigations: the MIBBI project.Nature Biotechnology.26:8.889-96.2008.10.1038/nbt.1411 |
| Sundari et al. | 2007 | not a reporting guideline | Sundari, S..TRANSFERRING RESEARCH INTO POLICY AND PRACTISE.Buletin Penelitian Kesehatan.35:4.148-155.2007. |
| Stroup et al. | 2017 | not a guideline for reporting implementation or scaling | Stroup, D. F.; Smith, C. K.; Truman, B. I..Reporting the methods used in public health research and practice..1:..2017.10.21037/jphe.2017.12.01 |
| Lara et al. | 2011 | not a reporting guideline | Lara, M.; Bryant-Stephens, T.; Damitz, M.; Findley, S.; Gavillan, J. G.; Mitchell, H.; Ohadike, Y. U.; Persky, V. W.; Valencia, G. R.; Smith, L. R.; Rosenthal, M.; Thyne, S.; Uyeda, K.; Viswanathan, M.; Woodell, C..Balancing "fidelity" and community context in the adaptation of asthma evidence-based interventions in the "real world".Health Promotion Practice.12:6 Suppl 1.63S-72S.2011.10.1177/1524839911414888 |
| Lane et al. | 2012 | not a reporting guideline | Lane, R. I.; Berkowitz, J. M.; Sullivan, S. T.; Rose, J.; Bernichon, T.; Favoretto, A.; Shifflett, P.; Miles, E.; Jones, M..Applying the interactive systems framework to the dissemination and adoption of national and state recommendations for hypertension.American Journal of Community Psychology.50:3-4.541-52.2012.10.1007/s10464-012-9511-0 |
| Kondo et al. | 2015 | not a guideline for reporting implementation or scaling | Kondo, K.; Damberg, C.; Mendelson, A.; Motu'apuaka, M.; Freeman, M.; O'Neil, M.; Relevo, R.; Kansagara, D..Understanding the Intervention and Implementation Factors Associated with Benefits and Harms of Pay for Performance Programs in Healthcare.Department of Veterans Affairs.05:.05.2015. |
| Kihembo et al. | 2018 | not a guideline for reporting implementation or scaling | Kihembo, C.; Masiira, B.; Nakiire, L.; Katushabe, E.; Natseri, N.; Nabukenya, I.; Komakech, I.; Okot, C. L.; Adatu, F.; Makumbi, I.; Nanyunja, M.; Woldetsadik, S. F.; Tusiime, P.; Nsubuga, P.; Fall, I. S.; Wondimagegnehu, A..The design and implementation of the re-vitalised integrated disease surveillance and response (IDSR) in Uganda, 2013-2016.BMC Public Health.18:1.879.2018.10.1186/s12889-018-5755-4 |
| Kennedy et al. | 2014 | not a reporting guideline | Kennedy, M. S..Getting writing right..114:3.7.2014.10.1097/01.NAJ.0000444469.71069.e1 |
| Kearney et al. | 2017 | not a reporting guideline | Kearney, M. H..The Discussion Section Tells Us Where We Are.Research in Nursing & Health.40:4.289-291.2017.10.1002/nur.21803 |
| Kanji et al. | 2015 | not a guideline for reporting implementation or scaling | Kanji, S.; Hayes, M.; Ling, A.; Shamseer, L.; Chant, C.; Edwards, D. J.; Edwards, S.; Ensom, M. H.; Foster, D. R.; Hardy, B.; Kiser, T. H.; la Porte, C.; Roberts, J. A.; Shulman, R.; Walker, S.; Zelenitsky, S.; Moher, D..Reporting Guidelines for Clinical Pharmacokinetic Studies: The ClinPK Statement.Clinical Pharmacokinetics.54:7.783-95.2015.10.1007/s40262-015-0236-8 |
| Johnson et al. | 2008 | not a reporting guideline | Johnson, T. M..Tips on how to write a paper.Journal of the American Academy of Dermatology.59:6.1064-9.2008.10.1016/j.jaad.2008.07.007 |
| Jack et al. | 2018 | not a guideline for reporting implementation or scaling | Jack, L., Jr..Promoting the Science and Practice of Implementation Evaluation in Public Health.Preventing Chronic Disease.15:.E163.2018.10.5888/pcd15.180645 |
| Harvey et al. | 2011 | not a reporting guideline | Harvey, L. A.....But is the outcome meaningful? JNPT's recommendations for reporting results of controlled trials.Journal of Neurologic Physical Therapy.35:3.103-4.2011.10.1097/NPT.0b013e31822a2dde |
| Hartley et al. | 2004 | not a reporting guideline | Hartley, J..Down with "op. cit.".Journal of the Medical Library Association.92:4.393; author reply 393.2004. |
| Harris et al. | 2000 | not a reporting guideline | Harris, J. S.; Glass, L. S.; Ossler, C.; Low, P..Evidence-based design: the ACOEM Practice Guidelines Dissemination Project.Journal of Occupational & Environmental Medicine.42:4.352-61.2000. |
| Guise et al. | 2014 | not a guideline for reporting implementation or scaling | Guise, J. M.; Chang, C.; Viswanathan, M.; Glick, S.; Treadwell, J.; Umscheid, C. A.; Whitlock, E.; Fu, R.; Berliner, E.; Paynter, R.; Anderson, J.; Motu'apuaka, P.; Trikalinos, T..Agency for Healthcare Research and Quality Evidence-based Practice Center methods for systematically reviewing complex multicomponent health care interventions.Journal of Clinical Epidemiology.67:11.1181-91.2014.10.1016/j.jclinepi.2014.06.010 |
| Gsporer et al. | 2018 | not a reporting guideline | Gsporer, I.; Schrems, B. M..[Transparency and replicability of nursing intervention studies in long-term care: A selective literature review]..133:.1-8.2018.10.1016/j.zefq.2017.11.006 |
| Gross et al. | 2001 | not a reporting guideline | Gross, P. A.; Greenfield, S.; Cretin, S.; Ferguson, J.; Grimshaw, J.; Grol, R.; Klazinga, N.; Lorenz, W.; Meyer, G. S.; Riccobono, C.; Schoenbaum, S. C.; Schyve, P.; Shaw, C..Optimal methods for guideline implementation: conclusions from Leeds Castle meeting.Medical Care.39:8 Suppl 2.II85-92.2001. |
| Graham et al. | 2008 | not a reporting guideline | Graham, J. D..Is reporting of quality scores worth refining?.Annals of Internal Medicine.148:11.883.2008. |
| Glasgow et al. | 2014 | not a guideline for reporting implementation or scaling | Glasgow, R. E.; Phillips, S. M.; Sanchez, M. A..Implementation science approaches for integrating eHealth research into practice and policy..83:7.e1-11.2014.10.1016/j.ijmedinf.2013.07.002 |
| Glaser et al. | 1973 | not a reporting guideline | Glaser, Edward M.; Taylor, Samuel H..Factors influencing the success of applied research.American Psychologist.28:2.140-146.1973.10.1037/h0034203 |
| Gedda et al. | 2015 | not a guideline for reporting implementation or scaling | Gedda, Michel; Riche, Benjamin.Traduction française des lignes directrices SAMPL pour l’écriture et la lecture des méthodes et analyses statistiques..15:157.69-74.2015.10.1016/j.kine.2014.11.010 |
| Gedda et al. | 2015 | not a guideline for reporting implementation or scaling | Gedda, Michel.Traduction française des lignes directrices CARE pour l’écriture et la lecture des études de cas..15:157.64-68.2015.10.1016/j.kine.2014.11.009 |
| Connor-Smith et al. | 2003 | not a guideline for reporting implementation or scaling | Connor-Smith, Jennifer K.; Weisz, John R..Applying Treatment Outcome Research in Clinical Practice: Techniques for Adapting Interventions to the Real World.Child and Adolescent Mental Health.8:1.3-10.2003.10.1111/1475-3588.00038 |
| Coburn-Miller et al. | 2015 | not a reporting guideline | Coburn-Miller, C.; Casey, S.; Luong, Q.; Cameron, N.; Hocevar-Trnka, J.; Leung, D. H.; Gelfond, D.; Heubi, J. E.; Ramsey, B.; Borowitz, D..Standardization of Research-Quality Anthropometric Measurement of Infants and Implementation in a Multicenter Study.Clinical and translational science.8:4.330-3.2015.10.1111/cts.12283 |
| Citrome et al. | 2014 | not a reporting guideline | Citrome, L..Are you a Sunshine Superman? The US Sunshine Act and reporting requirements.International Journal of Clinical Practice.68:10.1175-6.2014.10.1111/ijcp.12539 |
| Choi et al. | 2016 | not a guideline for reporting implementation or scaling | Choi, Jiae; Choi, Tae-Young; Jun, Ji Hee; Lee, Ju Ah; Lee, Myeong Soo.Preferred Reporting Items for the Development of Evidence-based Clinical Practice Guidelines in Traditional Medicine (PRIDE-CPG-TM): Explanation and elaboration.European Journal of Integrative Medicine.8:6.905-915.2016.10.1016/j.eujim.2016.07.027 |
| Chen et al. | 2017 | not a guideline for reporting implementation or scaling | Chen, Y.; Yang, K.; Marusic, A.; Qaseem, A.; Meerpohl, J. J.; Flottorp, S.; Akl, E. A.; Schunemann, H. J.; Chan, E. S.; Falck-Ytter, Y.; Ahmed, F.; Barber, S.; Chen, C.; Zhang, M.; Xu, B.; Tian, J.; Song, F.; Shang, H.; Tang, K.; Wang, Q.; Norris, S. L.; Right Working Group.A Reporting Tool for Practice Guidelines in Health Care: The RIGHT Statement.Annals of Internal Medicine.166:2.128-132.2017.10.7326/M16-1565 |
| Chen et al. | 2013 | not a guideline for reporting implementation or scaling | Chen, E. K.; Reid, M. C.; Parker, S. J.; Pillemer, K..Tailoring evidence-based interventions for new populations: a method for program adaptation through community engagement.Evaluation & the Health Professions.36:1.73-92.2013.10.1177/0163278712442536 |
| Chase et al. | 2009 | not a guideline for reporting implementation or scaling | Chase, D.; Rosten, C.; Turner, S.; Hicks, N.; Milne, R..Development of a toolkit and glossary to aid in the adaptation of health technology assessment (HTA) reports for use in different contexts.Health Technology Assessment (Winchester, England).13:59.1-142, iii.2009.10.3310/hta13590 |
| Chantebel et al. | 2019 | not a guideline for reporting implementation or scaling | Chantebel, R.; Chesneau, A.; Tavernier, E.; El-Hage, W.; Caille, A..Completeness of Descriptions of Repetitive Transcranial Magnetic Stimulation Intervention: A Systematic Review of Randomized Controlled Trials of rTMS in Depression.Journal of ECT.35:1.7-13.2019.10.1097/YCT.0000000000000546 |
| Chan et al. | 2013 | not a reporting guideline | Chan, W.; Pearson, T..Guideline implementability appraisal (GLIA) in us national guidelines.BMJ Quality and Safety.22:.A31-A32.2013.10:1136/bmjqs-2013-002293.92 |
| Chambers et al. | 2017 | not a reporting guideline | Chambers, D. A..Dissemination and Implementation Research: From a Reporting Framework to Precision Medicine.American Journal of Public Health.107:6.839-840.2017.10.2105/AJPH.2017.303788 |
| CentersofDisease et al. | 2001 | not a guideline for reporting implementation or scaling | Centers of Disease, Control; Prevention,.Strategies for reducing morbidity and mortality from diabetes through health-care system interventions and diabetes self-management education in community settings. A report on recommendations of the Task Force on Community Preventive Services.MMWR Recomm Rep.50:RR-16.1-15.2001. |
| Carson et al. | 2003 | not a reporting guideline | Carson, Paul L.; Giger, Maryellen.Biomedical imaging research opportunities workshop.Academic Radiology.10:8.882-886.2003.10.1016/s1076-6332(03)00059-x |
| Carpenter et al. | 2017 | not a guideline for reporting implementation or scaling | Carpenter, C. R.; Pinnock, H..Starry Aims to Overcome Knowledge Translation Inertia: The Standards for Reporting Implementation Studies (StaRI) Guidelines.Academic Emergency Medicine.24:8.1027-1029.2017.10.1111/acem.13235 |
| Campbell et al. | 2003 | not a guideline for reporting implementation or scaling | Campbell, B. B.; Reerink, I. H.; Jenniskens, F.; Pathak, L. R..A framework for developing reproductive health policies and programmes in Nepal.Reproductive Health Matters.11:21.171-82.2003. |
| Brundage et al. | 2012 | not a guideline for reporting implementation or scaling | Brundage, Michael D.; Snyder, Claire F..Patient-reported outcomes in clinical practice: using standards to break down barriers.Clinical Investigation.2:4.343-346.2012.10.4155/cli.12.23 |
| Britto et al. | 2018 | not a guideline for reporting implementation or scaling | Britto, P. R.; Singh, M.; Dua, T.; Kaur, R.; Yousafzai, A. K..What implementation evidence matters: scaling-up nurturing interventions that promote early childhood development.Annals of the New York Academy of Sciences.1419:1.5-16.2018.10.1111/nyas.13720 |
| Boonstra et al. | 2014 | not a guideline for reporting implementation or scaling | Boonstra, A.; Versluis, A.; Vos, J. F..Implementing electronic health records in hospitals: a systematic literature review.BMC Health Services Research.14:.370.2014.10.1186/1472-6963-14-370 |
| Bomzon et al. | 2017 | not a guideline for reporting implementation or scaling | Bomzon, A..You and your research report: implementing the ARRIVE reporting guideline.Laboratory Animals.51:2.121-123.2017.10.1177/0023677216679442 |
| Biggs et al. | 2019 | not a guideline for reporting implementation or scaling | Biggs, E. E.; Carter, E. W.; Gilson, C. B..A Scoping Review of the Involvement of Children's Communication Partners in Aided Augmentative and Alternative Communication Modeling Interventions.American Journal of Speech-Language Pathology.28:2.743-758.2019.10.1044/2018_AJSLP-18-0024 |
| Balls et al. | 1995 | not a reporting guideline | Balls, M.; Goldberg, A. M.; Fentem, J. H.; Broadhead, C. L.; Burch, R. L.; Festing, M. F.; Frazier, J. M.; Hendriksen, C. F.; Jennings, M.; van der Kamp, M. D.; Morton, D. B.; Rowan, A. N.; Russell, C.; Russell, W. M.; Spielmann, H.; Stephens, M. L.; Stokes, W. S.; Straughan, D. W.; Yager, J. D.; Zurlo, J.; van Zutphen, B. F..The three Rs: the way forward: the report and recommendations of ECVAM Workshop 11.ATLA-Alternatives to Laboratory Animals.23:6.838-66.1995. |
| Anonymous | 2006 | not a reporting guideline | Anonymous,.CIPIH report: main recommendations.Bulletin of the World Health Organization.84:5.351.2006./S0042-96862006000500011 |
| Anonymous | 2002 | not a reporting guideline | Anonymous,.Federation, AHA, AAMC unveil landmark quality reporting initiative.Hospital Outlook.5:6.1, 4.2002. |
| Anonymous | 1998 | not a reporting guideline | Anonymous,.Identifiers are coming, but names or numbers?.AIDS Alert.13:8.93-4.1998. |
| Anonymous | 2006 | not a reporting guideline | Anonymous,.Make the most of publicly reported quality data.Hospital Peer Review.31:9.121-2.2006. |
| Anonymous | 2003 | not a reporting guideline | Anonymous,.NC hospital issues quality report cards.Hospital Peer Review.28:6.77-8, 83.2003. |
| Anonymous | 2003 | not a guideline for reporting implementation or scaling | Anonymous,.New JCAHO Quality Report provides meaningful, relevant information.Joint Commission Perspectives.23:7.1, 3-5.2003. |
| Anonymous | 2017 | not a reporting guideline | Anonymous,.On data availability, reproducibility and reuse.Nature Cell Biology.19:4.259.2017.10.1038/ncb3506 |
| Anonymous | 2004 | not a guideline for reporting implementation or scaling | Anonymous,.Public information policy revised to delete reference to standards areas in Quality Report.Joint Commission Perspectives.24:8.14.2004. |
| Anonymous | 2004 | not a guideline for reporting implementation or scaling | Anonymous,.Quality report receives additional improvements prior to launch.Joint Commission Perspectives.24:1.22, 25.2004. |
| Anonymous | 2003 | not a guideline for reporting implementation or scaling | Anonymous,.Quality reports. CMS and JCAHO strive to help consumers make better healthcare decisions.Health Care Food & Nutrition Focus.20:8.9.2003. |
| AmericanSocietyofClinical et al. | 2013 | not a guideline for reporting implementation or scaling | American Society of Clinical, Oncology.American Society of Clinical Oncology: Policy for relationships with companies.Journal of Clinical Oncology.31:16.2043-6.2013.10.1200/JCO.2013.49.5002 |
| Alonge et al. | 2019 | not a guideline for reporting implementation or scaling | Alonge, O.; Rodriguez, D. C.; Brandes, N.; Geng, E.; Reveiz, L.; Peters, D. H..How is implementation research applied to advance health in low-income and middle-income countries?..4:2.e001257.2019.10.1136/bmjgh-2018-001257 |
| Alder et al. | 1986 | not a reporting guideline | Alder, H. C..Guideline report. Implementing laser technology in the community hospital.Hospital Technology Series.5:9.1-25.1986. |
| Adelaja et al. | 2014 | not a reporting guideline | Adelaja, Abidemi; Wunder, Kristin; Gates, George; Lee, Adora Iris; Rogers, Susan; Bill, Colin.Capacity building assistance: The 360degree approach and lessons learned.Public health yearbook 2013.:.51-62.2014. |
| Adams et al. | 2018 | not a guideline for reporting implementation or scaling | Adams, M. J.; Harris, R. N.; Grant, E. H. C.; Gray, M. J.; Camille Hopkins, M.; Iverson, S. A.; Likens, R.; Mandica, M.; Olson, D. H.; Shepack, A.; Waddle, H..Prepublication Communication of Research Results.Ecohealth.15:3.478-481.2018.10.1007/s10393-018-1352-3 |
| Abernethy et al. | 2017 | not a guideline for reporting implementation or scaling | Abernethy, A. P.; Gippetti, J.; Parulkar, R.; Revol, C..Use of Electronic Health Record Data for Quality Reporting.Journal of oncology practice/American Society of Clinical Oncology.13:8.530-534.2017.10.1200/JOP.2017.024224 |
| Zaveri et al. | 2010 | not a reporting guideline | Zaveri, A.; Cofiel, L.; Shah, J.; Pradhan, S.; Chan, E.; Dameron, O.; Pietrobon, R.; Ang, B. T..Achieving high research reporting quality through the use of computational ontologies.Neuroinformatics.8:4.261-71.2010.10.1007/s12021-010-9079-5 |
| Yamato et al. | 2016 | not a reporting guideline | Yamato, T.; Maher, C.; Saragiotto, B.; Moseley, A.; Hoffmann, T.; Elkins, M.; Camargo, P. R..The TIDieR checklist will benefit the physical therapy profession..20:3.191-3.2016.10.1590/bjpt-rbf.2014.0182 |
| Wilson et al. | 2017 | not a reporting guideline | Wilson, P. M.; Sales, A.; Wensing, M.; Aarons, G. A.; Flottorp, S.; Glidewell, L.; Hutchinson, A.; Presseau, J.; Rogers, A.; Sevdalis, N.; Squires, J.; Straus, S..Enhancing the reporting of implementation research.Implementation Science.12:1.13.2017.10.1186/s13012-017-0546-3 |
| Whitman et al. | 2005 | not a reporting guideline | Whitman, M.; Keener, J..Good publication practices.AMWA Journal: American Medical Writers Association Journal.20:4.159-160.2005. |
| Wharton et al. | 2015 | not a reporting guideline | Wharton, Tracy.Rigor, Transparency, and Reporting Social Science Research.Research on Social Work Practice.27:4.487-493.2015.10.1177/1049731515622264 |
| Wang et al. | 2010 | not a guideline for reporting implementation or scaling | Wang, L.; Li, Y.; Li, J.; Zhang, M.; Xu, L.; Yuan, W.; Wang, G.; Hopewell, S..Quality of reporting of trial abstracts needs to be improved: using the CONSORT for abstracts to assess the four leading Chinese medical journals of traditional Chinese medicine.Trials [Electronic Resource].11:.75.2010.10.1186/1745-6215-11-75 |
| vonGroote et al. | 2014 | not a guideline for reporting implementation or scaling | von Groote, P. M.; Giustini, A.; Bickenbach, J. E..Analysis and implementation of a World Health Organization health report: methodological concepts and strategies.Am J Phys Med Rehabil.93:1 Suppl 1.S12-26.2014.10.1097/PHM.0000000000000017 |
| vonElm et al. | 2008 | not a guideline for reporting implementation or scaling | von Elm, E.; Altman, D. G.; Egger, M.; Pocock, S. J.; Gotzsche, P. C.; Vandenbroucke, J. P.; Strobe Initiative.The Strengthening the Reporting of Observational Studies in Epidemiology (STROBE) statement: guidelines for reporting observational studies.Journal of Clinical Epidemiology.61:4.344-9.2008.10.1016/j.jclinepi.2007.11.008 |
| Volk et al. | 2018 | not a reporting guideline | Volk, R. J.; Coulter, A..Advancing the science of patient decision aids through reporting guidelines.BMJ Qual Saf.27:5.337-339.2018.10.1136/bmjqs-2017-007657 |
| Vercellesi et al. | 2010 | not a guideline for reporting implementation or scaling | Vercellesi, Luisa; Minghetti, Paola; Di Croce, Marianna; Bazzi, Adriana; Pieroni, Bruno; Centemeri, Carlo; Bruno, Flavia.Recommendations for health reporting: Proposal of a working paper.Health Education Journal.69:1.48-62.2010.10.1177/0017896909349300 |
| Toews et al. | 2017 | not a reporting guideline | Toews, I.; Binder, N.; Wolff, R. F.; Toprak, G.; von Elm, E.; Meerpohl, J. J..Guidance in author instructions of hematology and oncology journals: A cross sectional and longitudinal study.PLoS ONE [Electronic Resource].12:4.e0176489.2017.10.1371/journal.pone.0176489 |
| Geiger et al. | 2018 | not a reporting guideline | Geiger, Tagungsleitung Prof Dr H.; Main, Frankfurt am; Weil, Prof Dr J.; Lübeck,.42. Wissenschaftlicher Kongress Deutsche Hochdruckliga e.V. DHL® \| Deutsche Gesellschaft für Hypertonie und Prävention; 22. – 24. November 2018, Berlin.Nieren- und Hochdruckkrankheiten.47:11.541-579.2018.10.5414/nhx02005 |
| Pronk et al. | 1997 | not a guideline for reporting implementation or scaling | Pronk, M. H.; Brorens, M. J. A.; Eschauzier, A. M.; Hardens, M.; Hekster, Y. A.; Van Der Kuy, A.; Lockefeer, J. H. M.; De Smet, P. A. G. M..Proposal for a standard report format for economic evaluations.Pharmaceutisch Weekblad.132:9.268-271.1997. |
| Raiola et al. | 2007 | not a reporting guideline | Raiola, G.; De Sanctis, V.; Bertelloni, S.; Chiavetta, S.; Ranieri, L.; De Simone, M.; Garofalo, P.; Govoni, M. R..Adolescent health care in Italy: Strategies for intervention statement from the Italian Society for Adolescent Medicine.Rivista Italiana di Medicina dell'Adolescenza.5:3.5-7.2007. |
| Tang et al. | 2018 | not a reporting guideline | Tang, Kit Yi.Self-regulated learning and writing Instruction: Programme development and evaluation.Dissertation Abstracts International Section A: Humanities and Social Sciences.79:8-A(E).No Pagination Specified.2018. |
| Das et al. | 2012 | not a reporting guideline | Das, J. P.; Draman, M. S.; Cormican, L.; O'Neill, J..Morning report: a recommendation to improve medical handover..105:2.59.2012. |
| Leech et al. | 2010 | not a guideline for reporting implementation or scaling | Leech, Nancy L.; Onwuegbuzie, Anthony J..Guidelines for conducting and reporting mixed research in the field of stress and coping and beyond..:.77-104.2010. |
| Pontious et al. | 2005 | not a reporting guideline | Pontious, J. M..Another subtle change.J Okla State Med Assoc.98:2.44.2005. |
| Prewitt et al. | 2003 | not a reporting guideline | Prewitt, E..Report: uniformed services.AMT Events.20:3.104-104.2003. |
| Sadiq et al. | 2017 | not a reporting guideline | Sadiq, S.; Asim, H. M.; Khan, I..Sub: Mandatory practice of reporting guidelines in rehabilitation research-ensuring quality work.Pakistan Journal of Medical and Health Sciences.11:2.809.2017. |
| Cleary et al. | 2018 | not a guideline for reporting implementation or scaling | Cleary, James F.; Osman, Hibah; Gafer, Nahla; Shrestha, Sudip; Ali, Zipporah; Temin, Sarah B..ASCO Guideline report: Palliative Care in the Global Setting—ASCO Resource-Stratified Practice Guideline.Journal of Clinical Oncology.36:34_suppl.85-85.2018.10.1200/JCO.2018.36.34_suppl.85 |
| To et al. | 2013 | not a reporting guideline | To, M. J.; Jones, J.; Emara, M.; Jadad, A. R..Are reports of randomized controlled trials improving over time? A systematic review of 284 articles published in high-impact general and specialized medical journals.PLoS ONE [Electronic Resource].8:12.e84779.2013.10.1371/journal.pone.0084779 |
| Thyer et al. | 2014 | not a reporting guideline | Thyer, Bruce A..Improving publication standards for psychotherapy outcome studies.Journal of Evidence-Based Psychotherapies.14:2.125-128.2014. |
| Thomas et al. | 2015 | not a reporting guideline | Thomas, K.; Farrell, M. B..How to write a protocol: part 1..43:1.1-7.2015.10.2967/jnmt.114.147793 |
| Tagney et al. | 2013 | not a reporting guideline | Tagney, J.; Palmer, S.; Morris, M.; Albarran, J. W.; Lockyer, L.; Burchardt, C.; Hall, G.; Parslow, J.; Ernst, S.; Osman, J.; Kavanagh, H.; Dayer, M. J.; Quinton, E.; Clift, P.; Hudsmith, L.; Thorne, S.; de Bono, J.; Pounds, G.; Mumford, S. L.; Jarman, J.; Brough, C. E. P.; McGee, C.; Rao, A.; Wright, D. J.; Brough, C. E. P.; McGee, C.; Rao, A.; Wright, D. J.; Ahmed, F. Z.; Allen, S.; Mamas, M.; Zaidi, A. M.; Cantor, E. J.; Carroz, P.; Schilling, R. J.; Barker, D.; Cullen, D.; Hall, R.; Ng Kam Chuen, M. J.; Hughes, S.; Sharpe, A.; Wright, D. J.; Rao, A.; Ng Kam Chuen, M. J.; Wright, D. J.; Hughes, S.; Belchambers, S.; Sendegaya, M.; Rao, A..Abstracts for Oral Presentation, Session 1, Hrc 2013.Europace.15:suppl 4.iv4-iv8.2013.10.1093/europace/eut314 |
| Tabuse et al. | 2012 | not a reporting guideline | Tabuse, H.; Yasui, H.; Funakoshi, T.; Hamauchi, S.; Tsushima, T.; Taniguchi, H.; Todaka, A.; Yokota, T.; Machida, N.; Yamasaki, K.; Fukutomi, A.; Onozawa, Y.; Boku, N.; Nakamura, M.; Shitara, K.; Yuki, S.; Takahari, D.; Kondo, C.; Tsuda, T.; Kii, T.; Tsuji, Y.; Ichikawa, D.; Utsunomiya, S.; Hosokawa, A.; Ishiguro, A.; Oze, I.; Muro, K.; Inoue, D.; Chin, K.; Ohara, K.; Takagi, K.; Ozaka, M.; Ogura, M.; Suenaga, M.; Shinozaki, E.; Matsusaka, S.; Mizunuma, N.; Hatake, K.; Takeda, H.; Tsumura, T.; Sekikawa, A.; Iguchi, E.; Kanesaka, T.; Saito, S.; Nasu, A.; Nishikawa, H.; Kita, R.; Maruo, T.; Okabe, Y.; Kimura, T.; Osaki, Y.; Wakasa, T.; Kitao, A.; Matsumoto, K.; Onoe, T.; Tanioka, M.; Negoro, S.; Suyama, H.; Makima, M.; Sumikawa, T.; Matsumura, A.; Hino, N.; Yamaguchi, T.; Iwase, S.; Kuroda, Y.; Yamamoto, D.; Tsubota, Y.; Goto, Y.; Sakata, N.; Mitchell, S.; Basch, E.; Morishita, T.; Sahashi, S.; Miyao, K.; Saito, T.; Sakemura, R.; Watanabe, K.; Niimi, K.; Ono, Y.; Sawa, M.; Nomura, M.; Kamata, M.; Kojima, H.; Hayashi, K.; Sawada, S.; Kawaguchi, K.; Ishiguro, H.; Morita, S.; Nakamura, S.; Ohno, S.; Masuda, N.; Iwata, H.; Aogi, K.; Kuroi, K.; Toi, M.; Tamiya, M.; Hirashima, T.; Kobayashi, M.; Morita, S.; Morimura, O.; Shiroyama, T.; Okafuji, K.; Matsuura, Y.; Morishita, N.; Suzuki, H.; Iwata, K.; Ryota, N.; Hino, H.; Okamoto, N.; Kawase, I.; Kenmotsu, H.; Hosokawa, M.; Koh, Y.; Yoshino, T.; Yoshikawa, T.; Naito, T.; Takahashi, T.; Murakami, H.; Watanabe, R.; Ono, S.; Kikuhara, Y.; Kanbara, H.; Matsunaga, T.; Yamamoto, N.; Takano, A.; Nakamura, Y.; Daigo, Y.; Kubo, A.; Kanaji, N.; Bandoh, S.; Ishii, T.; Fujita, J.; Matsunaga, T.; Yamaguchi, E.; Toyokawa, G.; Hamamoto, R.; Sugio, K.; Ichinose, Y.; Nakamura, Y.; Konagai, S.; Mori, M.; Shimada, I.; Kondoh, Y.; Shindou, N.; Soga, T.; Furutani, T.; Sakagami, H.; Ueno, Y.; Kaneko, N.; Tanaka, R.; Fushiki, H.; Saito, R.; Kuromitsu, S.; Sakai, K.; Okamoto, I.; Takezawa, K.; Hirashima, T.; Kaneda, H.; Takeda, M.; Matsumoto, K.; Kimura, H.; Fujita, Y.; Nakagawa, K.; Arao, T.; Nishio, K.; Horinouchi, H.; Kubota, K.; Itani, H.; Wakui, H.; Nakamichi, S.; Kanda, S.; Nokihara, H.; Yamamoto, N.; Sekine, I.; Tamura, T.; Yoshida, T.; Niho, S.; Toda, M.; Umemura, S.; Yoh, K.; Goto, K.; Ohmatsu, H.; Izumi, K.; Ohe, Y.; Kato, T.; Yamaguchi, O.; Yoshida, M.; Fukushima, H.; Nakazawa, A.; Oda, T.; Takasa, A.; Enomoto, T.; Baba, T.; Kitamura, H.; Shinohara, T.; Nishihira, R.; Komatsu, S.; Hagiwara, E.; Ogura, T.; Nishigaki, Y.; Asahina, H.; Oizumi, S.; Fujita, Y.; Takamura, K.; Yamamoto, M.; Fuke, S.; Kojima, T.; Harada, T.; Kawai, Y.; Sasaki, T.; Konno, S.; Kinoshita, I.; Akita, H.; Nishimura, M.; Ko, R.; Sasaki, S.; Namba, Y.; Ishimori, A.; Yoshioka, M.; Yoshioka, Y.; Tominaga, S.; Takahashi, K.; Kirita, K.; Goto, K.; Umemura, S.; Yoh, K.; Niho, S.; Ohmatsu, H.; Nagai, K.; Ohe, Y.; Yomota, M.; Sekihara, K.; Nakahara, Y.; Ookuma, Y.; Takagi, Y.; Hosomi, Y.; Iguchi, M.; Okamura, T.; Shibuya, M.; Oizumi, S.; Inoue, A.; Sakakibara, T.; Asahina, H.; Sugawara, S.; Maemondo, M.; Okudera, K.; Suzuki, T.; Usui, K.; Harada, M.; Morikawa, N.; Hasegawa, Y.; Ishimoto, O.; Nukiwa, T.; Zhang, Y.; Hong, X. n; Chen, L. T.; Kuo, S. H.; Yeh, K. H.; Wu, M. S.; Lin, C. W.; Hsu, P. N.; Wang, H. P.; Cheng, A. L.; Lee, H. W.; Choi, J. H.; Ahn, M. S.; Kang, S. Y.; Jeong, S. H.; Park, J. S.; Han, J. H.; Kim, J. H.; Oda, H.; Ikeda, M.; Choi, I.; Suehiro, Y.; Abe, Y.; Uike, N.; Hyun, S. Y.; Jang, J.; Kim, Y. D.; Whang, D. Y.; Kim, S. J.; Kim, J. S.; Cheong, J. W.; Min, Y. H.; Tatsumi, Y.; Rai, S.; Kanai, Y.; Hirase, C.; Yamaguchi, T.; Morita, Y.; Tanaka, H.; Simada, T.; Kawanishi, K.; Miyaktake, J.; Ashida, T.; Matsumura, I.; Yamanaka, T.; Katsuya, H.; Ishitsuka, K.; Utsunomiya, A.; Sasaki, H.; Hanada, S.; Eto, T.; Moriuchi, Y.; Saburi, Y.; Miyahara, M.; Sueoka, E.; Uike, N.; Yoshida, S.; Suzumiya, J.; Tamura, K.; Ito, K.; Okamoto, M.; Kakumae, Y.; Ando, Y.; Ando, M.; Kumazawa, S.; Okamoto, A.; Inaguma, Y.; Ohta, H.; Yamada, S.; Emi, N.; Suzuki, T.; Harada, Y.; Matsubara, E.; Aoki, T.; Oyama, T.; Kasai, M.; Uchida, T.; Ogura, M.; Nakamura, N.; Hara, T.; Mabuchi, R.; Nagano, J.; Ogawa, K.; Kitagawa, J.; Kanemura, N.; Goto, N.; Tsurumi, H.; Moriwaki, H.; Nishiwaki, S.; Miyamura, K.; Ohashi, K.; Kurokawa, M.; Taniguchi, S.; Fukuda, T.; Ikegame, K.; Sakamaki, H.; Morishima, Y.; Kato, K.; Suzuki, R.; Tanaka, J.; Ichikawa, M.; Kobayashi, T.; Nannya, Y.; Kurokawa, M.; Kojima, Y.; Hagiwara, S.; Yamamoto, H.; Uehira, T.; Ajisawa, A.; Kitanaka, A.; Tanuma, J.; Okada, S.; Nagai, H.; Miyao, K.; Sahashi, S.; Sakemura, R.; Morishita, T.; Saito, T.; Watanabe, K.; Niimi, K.; Ono, Y.; Sawa, M.; Ikari, Y.; Nakashima, Y.; Sato, E.; Katsuya, H.; Goto, T.; Ishizu, M.; Tanaka, T.; Ogata, K.; Isutuka, K.; Takamatu, Y.; Tamura, K.; Sato, Y.; Tatsuta, R.; Nakahara, R.; Otsu, S.; Itoh, H.; Morinaga, R.; Shirao, K.; Narimatsu, H.; Sugawara, Y.; Fukao, A.; Chou, Y. C.; Chen, T. L.; Liao, C. C.; Fujisaka, Y.; Yamamoto, N.; Hirashima, T.; Takeda, K.; Sugio, K.; Satouchi, M.; Nakagawa, K.; Takahashi, S.; Ura, T.; Nakano, K.; Chin, K.; Yokoyama, M.; Hatake, K.; Yokota, T.; Shitara, K.; Muro, K.; Aoyama, T.; Kurata, T.; Murakami, H.; Fujisaka, Y.; Kiyota, H.; Hayashi, H.; Tanaka, K.; Nakagawa, K.; Onozawa, Y.; Watanabe, J.; Yamamoto, N.; Aoyama, T.; Honda, K.; Yamamoto, N.; Nokihara, H.; Yamada, Y.; Tamura, Y.; Asahina, H.; Suzuki, S.; Yamazaki, N.; Ogita, Y.; Hasegawa, W.; Matsuoka, M.; Tamura, T.; Onozawa, Y.; Murakami, H.; Ono, A.; Tushima, T.; Takahashi, T.; Yamazaki, K.; Nakamura, Y.; Jiko, T.; Boku, N.; Yamamoto, N.; Terashima, M.; Nakagawa, K.; Okabe, T.; Kaneda, H.; Yamamoto, N.; Nokihara, H.; Horinouchi, H.; Horai, T.; Nishio, M.; Ohyanagi, F.; Horiike, A.; McKee, M.; Carlson, D.; Xiong, H.; Tamura, T.; Nakagawa, T.; Takeuchi, S.; Yamada, T.; Yano, S.; Hirose, T.; Noda, H.; Okuda, K.; Abe, S.; Oto, Y.; Kusumoto, S.; Sugiyama, T.; Ishida, H.; Shirai, T.; Nakashima, M.; Yamaoka, T.; Ohmori, T.; Yoshida, K.; Nakamura, Y.; Adachi, M.; Tamura, Y.; Nokihara, H.; Yamamoto, N.; Wakui, H.; Honda, K.; Asahina, H.; Yamada, Y.; Komaba, T.; Tamura, T.; Wakui, H.; Yamamoto, N.; Nakamichi, S.; Tamura, Y.; Nokihara, H.; Yamada, Y.; Tamura, T.; Hayashi, H.; Arao, T.; Matsumoto, K.; Nagai, T.; Kimura, H.; De Velasco, M. A.; Fujita, Y.; Yamada, Y.; Nakagawa, K.; Nishio, K.; Kaneda, H.; Arao, T.; Tanaka, K.; Matsumoto, K.; Kimura, H.; Nagai, T.; Sakai, K.; Fujita, Y.; De Velasco, M. A.; Yamada, Y.; Tsurutani, J.; Okamoto, I.; Nakagawa, K.; Nishio, K.; Hashimoto, J.; Watanabe, M.; Uehara, Y.; Yamashita, N.; Fujimura, Y.; Nishio, K.; Nakamichi, M.; Fujiwara, Y.; Kanda, S.; Horinouchi, H.; Nokihara, H.; Yamamoto, N.; Koh, Y.; Tamura, T.; Koizumi, F.; Kaira, K.; Sunose, Y.; Arakawa, K.; Sunaga, N.; Iwasaki, Y.; Mori, M.; Oyama, T.; Takeyoshi, I.; Itoh, K.; Harada, T.; Takamura, K.; Kikuchi, E.; Ohizumi, S.; Sugawara, S.; Maemondo, M.; Fujita, Y.; Kinoshita, I.; Inoue, A.; Hommura, F.; Katsuura, Y.; Dosaka-Akita, H.; Isobe, H.; Nishimura, M.; Hirashima, T.; Okamoto, N.; Suzuki, H.; Morishita, N.; Tamiya, M.; Shiroyama, T.; Kondoh, Y.; Ryota, N.; Kawase, I.; Kanazawa, K.; Ishida, T.; Fujita, Y.; Fujiuchi, S.; Harada, T.; Harada, M.; Takamura, K.; Kinoshita, I.; Katsuura, Y.; Honjo, O.; Kojima, T.; Oizumi, S.; Isobe, H.; Munakata, M.; Nishimura, M.; Sakai, H.; Suzuki, K.; Niho, S.; Ikeda, N.; Kaburagi, T.; Koike, T.; Minato, K.; Kato, T.; Okamoto, H.; Seto, T.; Hosomi, Y.; Tsuboi, M.; Watanabe, K.; Takeuchi, M.; Ebi, N.; Maruyama, R.; Kishimoto, J.; Tsuruno, K.; Kato, M.; Yano, T.; Nagamatsu, Y.; Tsukamoto, S.; Akamine, S.; Saeki, S.; Ichinose, Y.; Niho, S.; Nokihara, H.; Nihei, K.; Akimoto, T.; Sumi, M.; Ito, Y.; Yoh, K.; Goto, K.; Ohmatsu, H.; Horinouchi, H.; Yamamoto, N.; Sekine, I.; Kubota, K.; Ohe, Y.; Tamura, T.; Nogami, N.; Kozuki, T.; Segawa, Y.; Shinkai, T.; Maeda, T.; Ueoka, H.; Harita, S.; Kuyama, S.; Hosokawa, S.; Gemba, K.; Takemoto, M.; Takigawa, N.; Tabata, M.; Tanimoto, M.; Kiura, K.; Kijima, T.; Shiroyama, T.; Komuta, K.; Yamamoto, S.; Minami, S.; Ogata, Y.; Okafuji, K.; Ihara, S.; Imamura, F.; Nishino, K.; Hirashima, T.; Tachibana, I.; Kawase, I.; Kumanogoh, A.; Tamiya, A.; Tamiya, M.; Kandu, M.; Asami, K.; Okishio, K.; Kawaguchi, T.; Atagi, S.; Suzuki, H.; Okamoto, N.; Kobayashi, S.; Shiroyama, N.; Morishita, N.; Tsuji, T.; Hirooka, A.; Hirashima, T.; Demura, Y.; Maemondo, M.; Ishii, Y.; Okudera, K.; Takamura, K.; Kobayashi, K.; Morikawa, N.; Gemma, A.; Ishimoto, O.; Harada, M.; Miura, S.; Fujita, Y.; Usui, K.; Saijo, Y.; Minegishi, Y.; Kobayashi, K.; Maemondo, M.; Inoue, A.; Sugawara, S.; Ohizumi, S.; Isobe, H.; Hagiwara, K.; Morita, S.; Nukiwa, T.; Gemma, A.; Yoh, K.; Goto, K.; Ohmatsu, H.; Niho, S.; Umemura, S.; Ohe, Y.; Ohmori, T.; Yamaoka, T.; Ichihashi, Y.; Hirose, T.; Saijo, N.; Azuma, K.; Okamoto, I.; Takeda, M.; Kuwano, M.; Ono, M.; Nakagawa, K.; Kato, Y.; Ichihara, E.; Hotta, K.; Hisamoto, A.; Takigawa, N.; Nogami, N.; Kozuki, T.; Kudo, K.; Tabata, M.; Shinkai, T.; Tanimoto, M.; Kiura, K.; Hata, A.; Katakami, N.; Yoshioka, H.; Tanaka, K.; Nishiyama, A.; Nanjo, S.; Kaji, R.; Fujita, S.; Monden, K.; Matsumoto, T.; Nagata, K.; Otsuka, K.; Tachikawa, R.; Tomii, K.; Iwasaku, M.; Omachi, N.; Kawaguchi, T.; Tamiya, A.; Mimori, T.; Takeuchi, N.; Matsuda, Y.; Asami, K.; Okishio, K.; Atagi, S.; Okuma, T.; Kubo, A.; Maruyama, Y.; Kudoh, S.; Takada, M.; Nishie, K.; Kuramochi, H.; Kanno, H.; Uchiyama, T.; Nakajima, G.; Saito, K.; Hayashi, K.; Nakadate, Y.; Kitamura, Y.; Tamura, T.; Koizumi, F.; Matsusaka, S.; Chin, K.; Ogur, M.; Shinozaki, E.; Suenaga, M.; Mizunuma, N.; Sano, T.; Yamaguchi, T.; Hatake, K.; Nomura, M.; Shitara, K.; Kodaira, T.; Kondoh, C.; Takahari, D.; Ura, T.; Kojima, H.; Kamata, M.; Muro, K.; Sawada, S.; Suzuki, A.; Xiao, L.; Taketa, T.; Blum, M.; Welsh, J.; Lin, S.; Bhutani, M.; Lee, J.; Rice, D.; Maru, D.; Erasmus, J.; Hofstetter, W.; Stephen, S.; Onodera, H.; Ajani, J.; Takahashi, N.; Yamada, Y.; Taniguchi, H.; Akiyoshi, K.; Honma, Y.; Iwasa, S.; Kato, K.; Hamaguchi, T.; Shimada, Y.; Shimodaira, H.; Soeda, H.; Gamoh, M.; Andoh, H.; Yamaguchi, T.; Watanabe, M.; Ishobe, H.; Sudo, T.; Kato, S.; Ishioka, C.; Kano, Y.; Tsuchiya, K.; Watanabe, M.; Yamazaki, K.; Bando, H.; Shinozaki, E.; Nishina, T.; Kadowaki, S.; Yuki, S.; Kajiura, S.; Tsuchihara, K.; Fujii, S.; Yamanaka, T.; Yoshino, T.; Nakamura, M.; Onikubo, T.; Nakamura, K.; Oda, K.; Nishida, Y.; Tauchi, K.; Kondoh, Y.; Hirashima, T.; Okamoto, N.; Ryouta, N.; Shimura, K.; Nakamura, Y.; Sandou, M.; Suzuki, H.; Tamiya, M.; Morishita, N.; Shiroyama, T.; Saijo, M.; Oomori, Y.; Iwata, K.; Kawase, I.; Nagashima, F.; Kitamura, H.; Miyajima, K.; Takasu, A.; Kasuga, A.; Furuse, J.; Onaya, H.; Ogawa, A.; Mori, T.; Ishioka, C.; Terazawa, T.; Kondo, S.; Morizane, C.; Yamaguchi, T.; Shimizu, S.; Shuichi, M.; Ikeda, M.; Ueno, H.; Okusaka, T.; Nakachi, K.; Okusaka, T.; Iguchi, H.; Shimamura, T.; Ioka, T.; Hosokawa, A.; Ikeda, M.; Morizane, C.; Asagi, A.; Junji, F.; Hirano, G.; Takii, Y.; Kamihira, T.; Fujisawa, K.; Ichiki, Y.; Suzuki, T.; Matsuo, T.; Uraoka, S.; Aoki, K. i; Sakai, K. i; Ogawa, R.; Higuchi, M.; Kobayashi, S.; Ueno, M.; Ohkawa, S.; Andou, T.; Kameda, R.; Kitazono, M.; Kobayashi, K.; Emi, Y.; Kakechi, Y.; Takahashi, T.; Akagi, Y.; Tuji, A.; Yoshida, K.; Baba, H.; Ogata, H.; Shimokawa, M.; Natsugoe, S.; Maehara, Y.; Shimasaki, T.; Kitano, A.; Sato, H.; Minamoto, T.; Motoo, Y.; Fujiwara, Y.; Chayahara, N.; Kiyota, N.; Nakano, K.; Mukohara, T.; Funakoshi, Y.; Imamura, Y.; Toyoda, M.; Shimada, T.; Tomioka, H.; Yakushijin, K.; Okamura, A.; Matsuoka, H.; Minami, H.; Miura, T.; Mitsunaga, S.; Matsumoto, N.; Nakazato, M.; Ohno, I.; Shimizu, S.; Takahashi, H.; Okuyama, H.; Kuwahara, A.; Ikeda, M.; Fukahori, M.; Kondo, S.; Ueno, H.; Shimizu, S.; Mitsunaga, S.; Ikeda, M.; Yamaguchi, T.; Sakamoto, Y.; Morizane, C.; Okusaka, T.; Nakai, Y.; Isayama, H.; Ijichi, H.; Sasaki, T.; Ito, Y.; Matsubara, S.; Yagioka, H.; Nagano, R.; Kawakubo, K.; Kogure, H.; Yamamoto, N.; Sasahira, N.; Hirano, K.; Tada, M.; Koike, K.; Morizane, C.; Ueno, H.; Kondo, S.; Mitsunaga, S.; Shimizu, S.; Ohno, I.; Takahashi, H.; Yamaguchi, T.; Sakamoto, Y.; Okusaka, T.; Kasuga, A.; Yamaguchi, T.; Machida, N.; Takahashi, H.; Sudo, K.; Nishina, T.; Nishisaki, H.; Ishido, K.; Okuno, T.; Moriwaki, T.; Kawai, H.; Kobayashi, S.; Hosokawa, A.; Furuse, J.; Boku, N.; Yokota, T.; Onitsuka, T.; Kusafuka, K.; Ogawa, H.; Onozawa, Y.; Nakagawa, M.; Iida, Y.; Kamijo, T.; Hirasawa, N.; Nishimura, T.; Nakajima, T.; Boku, N.; Yasui, H.; Yoshida, M.; Suzuki, S.; Kobayashi, S.; Yajima, Y.; Kobayashi, T.; Ishiki, H.; Tahara, M.; Izumi, K.; Yajima, Y.; Ishiki, H.; Kobayashi, T.; Tahara, M.; Suzuki, S.; Kobayashi, S.; Akimoto, T.; Zenda, S.; Nishimura, N.; Yokoyama, M.; Ohhara, Y.; Nara, E.; Nakano, K.; Ueda, K.; Mishima, Y.; Sakajiri, S.; Terui, Y.; Takahashi, S.; Hatake, K.; Uomori, T.; Yokoyama, M.; Nara, E.; Nakano, K.; Ueda, K.; Nishimura, N.; Sakajiri, S.; Mishima, Y.; Terui, Y.; Takahashi, S.; Ohhara, Y.; Nishimura, N.; Nara, E.; Nakano, K.; Ueda, K.; Sakajiri, S.; Mishima, Y.; Yokoyama, M.; Terui, Y.; Takahashi, S.; Hatake, K.; Yokota, T.; Onozawa, Y.; Boku, N.; Hamauchi, S.; Tsushima, T.; Taniguchi, H.; Todaka, A.; Machida, N.; Yamazaki, K.; Fukutomi, A.; Yasui, H.; Matsumoto, K.; Onoe, T.; Kitao, A.; Tanioka, M.; Negoro, S.; Okazawa, M.; Mabuchi, S.; Daimon, E.; Iwamiya, T.; Miyatake, T.; Ohta, Y.; Yoshino, K.; Fujita, M.; Enomoto, T.; Kamiura, S.; Kimura, T.; Kitagawa, R.; Katsumata, N.; Shibata, T.; Nakanishi, T.; Nishimura, S.; Nishio, S.; Takano, M.; Satoh, T.; Yokota, H.; Ochiai, K.; Kigawa, J.; Kobayashi, H.; Kanato, K.; Yoshikawa, H.; Kamura, T.; Kawaguchi, R.; Furukawa, N.; Kimura, M.; Kanno, M.; Fukada, I.; Ito, Y.; Takahashi, S.; Tsutsumi, C.; Kobayashi, T.; Kobayashi, K.; Nakayama, Y.; Kawai, Y.; Hatake, K.; Ohtani, S.; Kochi, M.; Abe, K.; Sakata, Y.; Hiraki, K.; Fujiwara, Y.; Iwamoto, Y.; Kang, S. Y.; Choi, J. H.; Lee, H. W.; Ahn, M.; Kim, K. S.; Nagura, K.; Koyama, T.; Gomi, D.; Mikawa, T.; Matsuda, M.; Oyama, Y.; Fukuma, E.; Shoji, K.; Niikura, N.; Masuda, S.; Terada, M.; Terao, M.; Kumaki, N.; Oshitanai, R.; Morioka, T.; Tsuda, B.; Okamura, T.; Saito, Y.; Suzuki, Y.; Tokuda, Y.; Park, Y. H.; Ahn, H. K.; Park, S.; Maeng, C. H.; Lee, S. J.; Ahn, J. S.; Im, Y. H.; Gonda, K.; Shibata, M.; Ohtake, T.; Shimura, T.; Sakurai, K.; Machida, T.; Outo, H.; Takenoshita, S.; Nakayama, Y.; Ito, Y.; Fukata, I.; Kobayashi, T.; Tsutsumi, C.; Takahashi, S.; Hatake, K.; Nagatsuma, A.; Shimizu, C.; Tsuda, H.; Saji, S.; Hojo, T.; Sugano, K.; Fujiwara, Y.; Tanizaki, J.; Tsurutani, J.; Matsuoka, H.; Kiyota, H.; Okamoto, K.; Kurata, T.; Nakagawa, K.; Baba, H.; Li, J.; Xu, R.; Xu, J.; Denda, T.; Ikejiri, K.; Shen, L.; Toh, Y.; Shimada, K.; Kato, T.; Sakai, K.; Matsuyama, A.; Mishima, H.; Wang, J.; Kawakami, K.; Nakamoto, E.; Yokokawa, T.; Mae, Y.; Sugita, K.; Suenaga, M.; Mizunuma, N.; Yamaguchi, T.; Hama, T.; Nakano, K.; Takahashi, S.; Nishimura, N.; Mishima, Y.; Sakajiri, S.; Yokoyama, M.; Terui, Y.; Motoi, N.; Hatake, K.; Takada, K.; Takimoto, R.; Yoshinaga, T.; Iyama, S.; Ono, K.; Murase, K.; Sato, T.; Miyanishi, K.; Sato, Y.; Kobune, M.; Soma, T.; Nagoya, S.; Yamashita, T.; Watanabe, A.; Kato, J.; Kato, S.; Ishida, T.; Ito, S.; Gamoh, M.; Saijo, Y.; Sato, J.; Shibata, H.; Yoshioka, T.; Ishioka, C.; Emi, Y.; Ogata, Y.; Akagi, Y.; Kakeji, Y.; Oki, E.; Saeki, H.; Shimokawa, M.; Touyama, T.; Samura, H.; Baba, H.; Natsugoe, S.; Shirouzu, K.; Tokunaga, S.; Maehara, Y.; Suenaga, M.; Matsusaka, S.; Shinozaki, E.; Ozaka, M.; Ogura, M.; Chin, K.; Hatake, K.; Mizunuma, N.; Yamaguchi, T.; Miyata, Y.; Nishina, T.; Kato, T.; Yamazaki, K.; Yoshino, T.; Esaki, T.; Moriwaki, T.; Hyodo, I.; Morita, S.; Inada, M.; Shibata, T.; Oguri, T.; Shimokata, T.; Sugishita, M.; Mitsuma, A.; Uehara, K.; Nakayama, G.; Ando, Y.; Nagase, M.; Yanai, K.; Ishida, Y.; Kumano, H.; Koinuma, K.; Miyakura, Y.; Horie, H.; Yasuda, Y.; Fujii, H..OS1. gastric cancer.Annals of Oncology.23:suppl 11.xi99-xi131.2012.10.1093/annonc/mds572 |
| Skolarus et al. | 2017 | not a reporting guideline | Skolarus, T. A.; Lehmann, T.; Tabak, R. G.; Harris, J.; Lecy, J.; Sales, A. E..Assessing citation networks for dissemination and implementation research frameworks.Implementation Science.12:1.97.2017.10.1186/s13012-017-0628-2 |
| Service et al. | 2008 | not a reporting guideline | Service, R. F..Science policy. Report faults U.S. strategy for nanotoxicology research.Science.322:5909.1779.2008.10.1126/science.322.5909.1779a |
| Schmidt et al. | 1996 | not a guideline for reporting implementation or scaling | Schmidt, K. L.; Alpen, M. A.; Rakel, B. A..Implementation of the Agency for Health Care Policy and Research Pain Guidelines.AACN Clinical Issues.7:3.425-35.1996. |
| Sare et al. | 2018 | not a guideline for reporting implementation or scaling | Sare, D.; Perez, D.; Some, P. A.; Kafando, Y.; Barro, A.; Ridde, V..Community-based dengue control intervention in Ouagadougou: intervention theory and implementation fidelity.Global Health Research and Policy.3:.21.2018.10.1186/s41256-018-0078-7 |
| Ried et al. | 2005 | not a reporting guideline | Ried, K.; Fuller, J..Building a culture of research dissemination in primary health care: the South Australian experience of supporting the novice researcher [corrected] [published erratum appears in AUST HEALTH REV 2005 May;29(2):252].Australian Health Review.29:1.6-11.2005. |
| Raths et al. | 2008 | not a reporting guideline | Raths, D..In it to win it. To boost the role of EHRs in quality reporting, CMS is seeking to ease PQRI participation.Healthcare Informatics.25:8.36-7.2008. |
| Pisetsky et al. | 2013 | not a guideline for reporting implementation or scaling | Pisetsky, D. S..Translation, treatises, and tweets.Arthritis care & research.65:6.839-42.2013.10.1002/acr.21974 |
| Phillips et al. | 2015 | not a guideline for reporting implementation or scaling | Phillips, W. D.; Christadoss, P.; Losen, M.; Punga, A. R.; Shigemoto, K.; Verschuuren, J.; Vincent, A..Guidelines for pre-clinical animal and cellular models of MuSK-myasthenia gravis.Experimental Neurology.270:.29-40.2015.10.1016/j.expneurol.2014.12.013 |
| Oliver et al. | 2011 | not a reporting guideline | Oliver, Debra Parker.Editorial.Research on Aging.33:5.499-500.2011.10.1177/0164027511415353 |
| Nielsen et al. | 2018 | not a guideline for reporting implementation or scaling | Nielsen, B.; Slinning, K.; Weie Oddli, H.; Drozd, F..Identification of Implementation Strategies Used for the Circle of Security-Virginia Family Model Intervention: Concept Mapping Study.JMIR Research Protocols.7:6.e10312.2018.10.2196/10312 |
| Nie et al. | 2018 | not a guideline for reporting implementation or scaling | Nie, Xiaolu; Guang, Pengya; Peng, Xiaoxia.Critical components for designing and implementing randomized controlled trials.Pediatric Investigation.2:2.124-130.2018.10.1002/ped4.12042 |
| Newhouse et al. | 2013 | not a guideline for reporting implementation or scaling | Newhouse, R.; Bobay, K.; Dykes, P. C.; Stevens, K. R.; Titler, M..Methodology issues in implementation science.Medical Care.51:4 Suppl 2.S32-40.2013.10.1097/MLR.0b013e31827feeca |
| Moss et al. | 1999 | not a guideline for reporting implementation or scaling | Moss, F.; Thompson, R..A new structure for quality improvement reports.Quality in Health Care.8:2.76.1999.10.1136/qshc.8.2.76 |
| Montgomery et al. | 2013 | not a guideline for reporting implementation or scaling | Montgomery, P.; Mayo-Wilson, E.; Hopewell, S.; Macdonald, G.; Moher, D.; Grant, S..Developing a reporting guideline for social and psychological intervention trials.American Journal of Public Health.103:10.1741-6.2013.10.2105/AJPH.2013.301447 |
| Maher et al. | 2016 | not a reporting guideline | Maher, D..Reporting guidelines for implementation and operational research.Public Health in Action.6:1.1.2016.10.5588/pha.16.0018 |
| Kalibala et al. | 2016 | not a guideline for reporting implementation or scaling | Kalibala, S.; Woelk, G. B.; Gloyd, S.; Jani, N.; Kay, L.; Sarna, A.; Okal, J.; Ndwiga, C.; Haberland, N.; Sinai, I..Experiences in implementation and publication of operations research interventions: gaps and a way forward.Journal of the International AIDS Society.19:5 Suppl 4.20842.2016.10.7448/IAS.19.5.20842 |
| Catala-Lopez et al. | 2014 | not a reporting guideline | Catala-Lopez, F.; Peiro, S.; Hutton, B.; Perez Andres, C.; Moher, D..[Declaration of transparency: promoting a more complete, honest and adequate publication of scientific articles].Revista Espanola de Salud Publica.88:2.181-6.2014.10.4321/S1135-57272014000200001 |
| Bolton et al. | 1996 | not a guideline for reporting implementation or scaling | Bolton, L. B.; Georges, C. A..National Black Nurses Association community collaboration model.J Natl Black Nurses Assoc.8:2.48-67.1996. |
| Gaglio et al. | 2014 | Duplicate or secondary reporting of the guideline | Gaglio, B; Phillips, Sm; Heurtin-Roberts, S; Sanchez, Ma; Glasgow, Re.How pragmatic is it? Lessons learned using PRECIS and RE-AIM for determining pragmatic characteristics of research.Implementation science.9:.96‐.2014.10.1186/s13012-014-0096-x |
| Britto et al. | 2018 | Not a reporting guideline | Britto, P. R.; Singh, M.; Dua, T.; Kaur, R.; Yousafzai, A. K..What implementation evidence matters: Scaling-up nurturing interventions that promote early childhood development..1419:(Britto P.R., pbritto@unicef.org) Early Childhood Development, UNICEF, New York, NY, United States.5-16.2018.10.1111/nyas.13720 |
| Aboud et al. | 2018 | Not a reporting guideline | Aboud, F. E.; Yousafzai, A. K.; Nores, M..State of the science on implementation research in early child development and future directions..1419:(Aboud F.E., frances.aboud@mcgill.ca) Department of Psychology, McGill University, Montreal, QC, Canada.264-271.2018.10.1111/nyas.13722 |
| CIPIH | 2006 | not a guideline for reporting implementation or scaling | <https://apps.who.int/iris/handle/10665/269646> |
| Schober et al | 2013 | not a guideline for reporting implementation or scaling | <https://pubmed-ncbi-nlm-nih-gov.acces.bibl.ulaval.ca/23881537/> |
| Montgomery et al | 2018 | not a guideline for reporting implementation or scaling | <https://pubmed-ncbi-nlm-nih-gov.acces.bibl.ulaval.ca/30060754/> |
| Gold et al. | 2016 | duplicate or secondary reporting of the guideline | https://pubmed-ncbi-nlm-nih-gov.acces.bibl.ulaval.ca/27113199/ |
| Browne et al. | 2014 | duplicate or secondary reporting of the guideline | https://www.wallacefoundation.org/knowledge-center/pages/scaling-up-staying-true.aspx |
| Bennett et al | 2017 | not a reporting guideline | Bennett, Sara, Shehrin Shaila Mahmood, Anbrasi Edward, Moses Tetui, and Elizabeth Ekirapa-Kiracho. 2017. 'Strengthening scaling up through learning from implementation: comparing experiences from Afghanistan, Bangladesh and Uganda', Health Research Policy and Systems, 15: 108. |
| Global, Futures Group |  | not a reporting guideline | Global, Futures Group. 'Approach for Addressing and Measuring Policy Development and Implementation in the Scale-Up of Family Planning and Maternal, Neonatal, and Child Health Programs'. |
| Greenhalgh et al | 2019 | not a reporting guideline | Greenhalgh, Trisha, and Chrysanthi Papoutsi. 2019. 'Spreading and scaling up innovation and improvement', BMJ, 365: l2068. |
| Greenhalgh et al | 2017 | not a reporting guideline | Greenhalgh, Trisha, Joseph Wherton, Chrysanthi Papoutsi, Jennifer Lynch, Gemma Hughes, Christine A'Court, Susan Hinder, Nick Fahy, Rob Procter, and Sara Shaw. 2017. 'Beyond Adoption: A New Framework for Theorizing and Evaluating Nonadoption, Abandonment, and Challenges to the Scale-Up, Spread, and Sustainability of Health and Care Technologies', Journal of Medical Internet Research, 19: e8775. |
| Hochgesang et al | 2017 | not a reporting guideline | Hochgesang, Mindy, Sophia Zamudio-Haas, Lissa Moran, Leopoldo Nhampossa, Laura Packel, Hannah Leslie, Janise Richards, and Starley B. Shade. 2017. 'Scaling-up health information systems to improve HIV treatment: An assessment of initial patient monitoring systems in Mozambique', International Journal of Medical Informatics, 97: 322-30. |
| Knippenberg et al | 2005 | not a reporting guideline | Knippenberg, Rudolf, Joy E. Lawn, Gary L. Darmstadt, Genevieve Begkoyian, Helga Fogstad, Netsanet Walelign, and Vinod K. Paul. 2005. 'Systematic scaling up of neonatal care in countries', The Lancet, 365: 1087-98. |
| Kumar et al | 2019 | not a reporting guideline | Kumar, Somesh, Priti Dave, Ashish Srivastava, Jelle Stekelenburg, Dinesh Baswal, Deepti Singh, Bulbul Sood, and Vikas Yadav. 2019. 'Harmonizing scientific rigor with political urgency: policy learnings for identifying accelerators for scale-up from the safe childbirth checklist programme in Rajasthan, India', BMC Health Services Research, 19. |
| Mansour et al | 2010 | not a reporting guideline | Mansour, Morsi, Joan Bragar Mansour, and Abdo Hasan El Swesy. 2010. 'Scaling up proven public health interventions through a locally owned and sustained leadership development programme in rural Upper Egypt', Human Resources for Health, 8: 1. |
| Pronovost et al | 2008 | not a reporting guideline | Pronovost, Peter J., Sean M. Berenholtz, and Dale M. Needham. 2008. 'Translating evidence into practice: a model for large scale knowledge translation', BMJ, 337: a1714. |
| Reis et al | 2016 | not a reporting guideline | Reis, Rodrigo S., Deborah Salvo, David Ogilvie, Estelle V. Lambert, Shifalika Goenka, Ross C. Brownson, and Committee Lancet Physical Activity Series 2 Executive. 2016. 'Scaling up physical activity interventions worldwide: stepping up to larger and smarter approaches to get people moving', Lancet (London, England), 388: 1337-48. |
| Walters et al | 2018 | not a reporting guideline | Walters, Laticha Elizabeth Marolana, Richard Ernest Scott, and Maurice Mars. 2018. 'Teledermatology scale-up frameworks: a structured review and critique', BMC Health Services Research, 18: 613. |
| Coffey et al | 2006 | not a guideline for reporting implementation or scaling | Coffey P, Riley R Reform of the International Institutions: Edward Elgar Publishing; 2006 2006 |
| Coles et al | 2020 | not a guideline for reporting implementation or scaling | Coles E, Anderson J, Maxwell M, Harris FM, Gray NM, Milner G, et al The influence of contextual factors on healthcare quality improvement initiatives: a realist review Syst Rev 2020;9(1):1-22 |
| Dickson et al | 2014 | not a guideline for reporting implementation or scaling | Dickson KE, Simen-Kapeu A, Kinney MV, Huicho L, Vesel L, Lackritz E, et al Every Newborn: health-systems bottlenecks and strategies to accelerate scale-up in countries Lancet 2014;384(9941):438-54 |
| Gaye et al | 2009 | not a guideline for reporting implementation or scaling | Gaye PA, Nelson D Effective scale-up: avoiding the same old traps Human Resources for Health 2009;7(1):2 |
| Gilson et al | 2010 | not a guideline for reporting implementation or scaling | Gilson L, Schneider H Commentary: Managing scaling up: what are the key issues? Health Policy and Planning 2010;25(2):97-8 |
| Huicho et al | 2005 | not a guideline for reporting implementation or scaling | Huicho L, Dávila M, Campos M, Drasbek C, Bryce J, Victora CG Scaling up Integrated Management of Childhood Illness to the national level: achievements and challenges in Peru Health Policy and Planning 2005;20(1):14-24 |
| Linn et al | 2008 | not a guideline for reporting implementation or scaling | Linn AH and JF. Scaling Up Through Aid: The Real Challenge [Internet]. Brookings. 1apr. J.-C. [cité 23 nov 2022]. Disponible sur: https://www.brookings.edu/research/scaling-up-through-aid-the-real-challenge/ |
| Spring | 2014 | not a guideline for reporting implementation or scaling | Spring Defining Scale-Up of Nutrition Projects SPRING2014 |
| Subramanian et al | 2011 | not a guideline for reporting implementation or scaling | Subramanian S, Naimoli J, Matsubayashi T, Peters DH Do we have the right models for scaling up health services to achieve the Millennium Development Goals? BMC Health Services Research 2011;11(1):336 |
| Ghiron et al | 2014 | duplicate or secondary reporting of the guideline | Ghiron L, Shillingi L, Kabiswa C, Ogonda G, Omimo A, Ntabona A, et al Beginning with sustainable scale up in mind: initial results from a population, health and environment project in East Africa Reproductive Health Matters 2014;22(43):84-92 |
| Bryce et al | 2011 | duplicate or secondary reporting of the guideline | Bryce J, Victora CG, Boerma T, Peters DH, Black RE Evaluating the scale-up for maternal and child survival: a common framework Int Health 2011;3(3):139-46 |
| Phillips et al | 2007 | duplicate or secondary reporting of the guideline | Phillips JF, Nyonator FK, Jones TC, Ravikumar S Evidence-based scaling up of health and family planning service innovations in Bangladesh and Ghana24 |
| Hartmann et al | 2008 | duplicate or secondary reporting of the guideline | Hartmann A, Linn JF A FRAMEWORK AND LESSONS FOR DEVELOPMENT EFFECTIVENESS FROM LITERATURE AND PRACTICE76 |
| World et al | 2013 | duplicate or secondary reporting of the guideline | World Health O, United States Agency for International D Guide to fostering change to scale up effective health services Geneva: World Health Organization; 2013 2013 50 p |
| MCHIP et al | 2005 | duplicate or secondary reporting of the guideline | Scale and Scaling Up: A CORE Group Background Paper on Scaling Up Maternal, Newborn and Child Health Services [Internet]. MCHIP. [cité 23 nov 2022]. Disponible sur: https://www.mchip.net/technical-resource/scale-and-scaling-up-a-core-group-background-paper-on-scaling-up-maternal-newborn-and-child-health-services/ |
| Hodgins et al | 2016 | duplicate or secondary reporting of the guideline | Hodgins S, Quissell K SCALE-UP AS IF IMPACT MATTERED71 |
| Díaz et al | 2006 | duplicate or secondary reporting of the guideline | Díaz J, Simmons R, Díaz M, Cabral F, Chinaglia M Scaling up family planning service innovations in Brazil: the influence of politics and decentralization22 |
| Hanson et al | 2010 | duplicate or secondary reporting of the guideline | Hanson K, Cleary S, Schneider H, Tantivess S, Gilson L Scaling up health policies and services in low- and middle-income settings BMC Health Services Research 2010;10(1):I1 |
| Simmons et al | 2007 | duplicate or secondary reporting of the guideline | Simmons R, Fajans P, Ghiron L Scaling up health service delivery: from pilot innovations to policies and programmes World Health Organization; 2007 2007 |
| Larson et al | 2015 | duplicate or secondary reporting of the guideline | Larson A, McPherson R, Posner J, LaFond A, Ricca J Scaling Up High-Impact Health Interventions in Complex Adaptive Systems: Lessons from MCHIP46 |
| Fajans et al | 2017 | duplicate or secondary reporting of the guideline | Fajans P, Thom NT, Whittaker M, Satia J, Mai TTP, Can TD, et al Strategic choices in scaling up: introducing injectable contraception and improving quality of care in Viet Nam Viet Nam21 |
| Igras et al | 2014 | duplicate or secondary reporting of the guideline | Igras S, Sinai I, Mukabatsinda M, Ngabo F, Jennings V, Lundgren R Systems approach to monitoring and evaluation guides scale up of the Standard Days Method of family planning in Rwanda Global Health: Science and Practice 2014;2(2):234-44 |
| Kaufman et al | 2005 | duplicate or secondary reporting of the guideline | Kaufman, J., Zhang, E., & Xie, Z. (2005). Quality of Care in China: From Pilot Project to National Program. |
