# Supplementary material for: Informing the development of the SUCCEED reporting guideline for studies on the scaling of health interventions: A systematic review
Source: Medicine (Baltimore). 2024 Feb 16;103(7):e37079. doi: 10.1097/MD.0000000000037079 (PMC10869056; doi:10.1097/MD.0000000000037079)
Supplement: Supplementary file 4 [file medi-103-e37079-s004.pdf]

Id	Title	Authors names	Number of male authors	Number of female authors	Number of authors with non identified sex	Corresponding author	Institution	Country	Year	language
S1	Development of a checklist to assess the quality o	Albrecht, Lauren ; Archibald, Mandy ;	0	4	0	Scott, Shannon D	University of Alberta	canada	2013	English
S2	Evaluating the public health impact of health prom	Glasgow, Russell; Vogt, Thomas; Boles, S	3	0	0	Glasgow, Russell E	AMC Cancer Researc	USA	1999	English
S3	A framework for scaling up health interventions: l	Barker, PM ;Reid, A ;Schall, MW ;	1	2	0	Barker, Pierre M	Institute for Healthca	USA	2016	english
S4	Protecting the power of interventions through pro	Vicki S, Conn ; Patricia S, Groves ;	0	2	0	Conn, Vicki S	University of Missou	USA	2011	English
S5	Improving the reporting quality of nonrandomized	Des Jarlais, Don C. ;Lyles, Cynthia ;Crepa	22	4	0	Des Jarlais, Don C	Beth Israel Medical (	USA	2004	English
S6	Standardization of quality initiative reporting	Duncan, James, R. ;Larson, David, B. ;Kru	3	0	0	Kruska, Jonathan B	Beth Israel Deacones	USA	2013	English
S7	Reviewing evidence on complex social interventio	Egan, M ; Bambra, C ; Petticrew, M ; Wh	2	2	0	Egan, M	University of Glasgo	UK	2008	English
S8	Reporting guidelines for implementation and oper	Hales, Simon ;Lesher-Trevino, Ana ;Ford	5	1	0	Hales, Simon	World Health Organ	Switzerland	2016	English
S9	Reporting standards for studies of tailored interve	Harrington, nancy Grant ;noar, Seth M	1	1	0	Harrington, nancy G	University of Kentuc	USA	2012	English
S10	Better reporting of interventions: template for int	Hoffmann, Tammy C ;Barbour, Virginia	8	8	0	Hoffmann, Tammy	Bond University	UK	2014	English
S11	Reporting on innovative public health intervention	Huston, Patricia	0	1	0	Huston, Patricia	Canadian Journal of	canada	2003	English
S12	Programme Reporting Standards (PRS) for improv	Kågesten, Anna E. ;Tunçalp, Özge ;Porte	3	3	0	Tunçalp, Özge	World Health Organi	Switzerland	2017	English
S13	Reporting of context and implementation in studie	Luoto Jill ;Shekelle Paul G ;Maglione Ma	1	4	0	Luoto, Jill	RAND Corporation	USA	2014	English
S14	The Oxford Implementation Index: a new tool for	Montgomery P ;Underhill K ;Gardner F ;	3	2	0	Montgomery, P	Centre for Evidence-	UK	2013	English
S15	A Framework for Enhancing the Value of Research	Neta Gila ;Glasgow Russell ;Carpenter Cl	4	3	0	Neta, Gila	National Cancer Insti	USA	2014	English
S16	Development of a framework for reporting health	O'Donnell Siobhan ;Li Linda C ;King Judy	0	7	0	Li, Linda C.	Arthritis Research Ce	canada	2010	English
S17	SQUIRE 2.0 (Standards for QUality Improvement R	Ogrinc G; Davies L; Goodman D; Batalde	4	2	0	Ogrinc, G	White River Junction	USA	2016	English
S18	Guidelines for reporting evaluations based on obs	Mariona, Portell ;Anguera, M. Teresa ;C	1	3	0	Portell, Mariona	Universitat Autònom	Spain	2015	English
S19	Implementation strategies: recommendations for	Proctor, Enola K ;Powell, Byron J ;McMil	2	1	0	Proctor, Enola K	Washington Univers	USA	2013	English
S20	Economic evaluations of public health implementa	Reeves, p. ;Edmunds, K. ;Searles, A. ;W	2	2	0	Reeves, P	University of Newcas	Australia	2019	English
S21	Is reporting on interventions a weak link in unders	Riley, Barbara L ;MacDonald, JoAnne ;M	0	7	0	Riley, Barbara L	University of Waterl	canada	2008	English
S22	Reporting guidelines for implementation research	Yousafzai, Aisha K ;Aboud, Frances E. ;n	0	4	0	Yousafzai, Aisha K.	Harvard University	USA	2018	English
S23	A reporting guide for implementation science artic	CCDR	na	na	na	na	Canada Communical	canada	2016	English
S24	A guide to scaling up population health interventio	Milat, Andrew J ; Newson, Robyn ; King,	5	3	0	Milat, Andrew J	NSW Ministry of Hea	Australia	2016	English
S25	Standards for Reporting Implementation Studies (	Pinnock, Hilary ; Barwick, Melanie ; Carp	5	7	0	Pinnock, H	University of Edinbu	UK	2017	English
S26	Guide for Monitoring Scale-up of Health Practices	Adamou, Bridgit ; Curran, Jen ; Wilson, L	0	8	0	na	MEASURE Evaluatior	USA	2014	English
S27	Evaluating the scale-up for maternal and child sur	Bryce, Jennifer ; Victora, Cesar G ; Boern	4	1	0	Bryce, Jennifer	Johns Hopkins Unive	USA	2011	English
S28	Every Newborn: health-systems bottlenecks and s	Dickson, Kim E ; Simen-Kapeu, Aline ; Kir	4	9	0	Dickson, Kim E	UNICEF	USA	2014	English
S29	Scaling Impact: Innovation for the Public Good	McLean, Robert ; Gargani, John	2	0	0	na	IDRC	canada	2019	English
S30	Pathways for scaling up public health intervention	Indig, Devon ; Lee, Karen ; Grunseit, Anr	2	3	0	Indig, Devon	University of Sydney	Australia	2017	English
S31	Beginning with the end in mind: planning pilot pro	WHO	na	na	na	na	WHO-ExpandNet	Switzerland	2011	English
S32	The MAPS toolkit: mHealth assessment and plann	WHO	na	na	na	na	World Health Organi	Switzerland	2015	English
S33	Ten dimensions of scaling up reproductive health	US Agency for International Developme	na	na	na	na	US Agency for Intern	USA	2002	English
S34	Scaling up health service delivery: from pilot innov	Simmons, Ruth; Fajans, Peter; Ghiron La	1	2	0	na	WHO	Switzerland	2007	English
S35	An approach to rapid scale up Using HIV/AIDS trea	WHO	na	na	na	na	WHO	Switzerland	2014	English
S36	Scaling Up Global Health Interventions: A Propose	Yamey, Gavin	1	0	0	Gavin Yamey	Evidence-to-Policy Ir	USA	2011	English
S37	Scaling Up—From Vision to Large-Scale Change A	Larry, Cooley; Richard, Kohl; Rajani, R. V	2	1	0	na	Management Systen	USA	2016	English
S38	A model for scale up of family health innovations i	Elizabeth H, Bradley; Leslie A, Curry; Lau	2	9	0	Elizabeth H, Bradley	Yale University	USA	2012	English
S39	Scale up of services for mental health in low-incon	Julian, Eaton; Layla, mcCay; Maya, Semr	5	4	0	Julian, Eaton	CBM International	Nigeria	2011	English

name of the Guideline	Acronym	Extension	Main Guideline	objectives	Aim of the guideline	Reporting guideline for implementation studies	Reporting guideline for scaling up studies	Guideline to design or conduct scaling up studies	Type of study other	Checklist	Comment checklist
Workgroup for Interventio	WIDER	no	na	This article describes one so	provide a framework to identify and provide	yes	no	no	na	yes	Table 1
reach, efficacy, adoption	RE-AIM	no	na	RE-AIM provides a framewc	conceptualize the public heal	no	no	yes	na	yes	http://www.re-
na	na	no	na	we describe a framework fc	a framework for taking healt	no	no	yes	na	no	na
na	na	no	na	This article provides guidanc	discusses intervention eleme	yes	no	no	na	yes	Table 1
Transparent Reporting o	TREND	no	na	present the initial versionof the	[illegible] standardize t	yes	no	no	na	yes	table 1
na	na	no	na	intends to standardize the v	facilitate the preparation anc	yes	no	no	na	yes	page 374
na	na	no	na	To appraise the quality of re	to appraise the reporting of i	yes	no	no	na	yes	table 2
na	na	no	na	describe the development c	Enhancing research quality a	yes	no	no	na	yes	Table 2
Reporting standards for s	na	no	na	The objective of this manus	recommendations for tailore	yes	no	no	na	yes	table 1
Template forintervention description and replication	TIDieR	yes	CONSORT SPIR	describes the methods usec	The overarching purpose of t	yes	no	no	na	yes	table 1
Reviewer Guidelines for I	na	no	na	The present article identifie	to offer a structured, yet stin	yes	no	no	na	yes	table 1
Programme Reporting St	PRS	no	na	present results from these	to provide guidance for com	yes	no	no	na	yes	Table 5
Global framework impler	na	no	na	to assess the current state c	to improve the design, cond	yes	no	no	na	yes	table 1
The Oxford Implementat	na	no	na	This article presents a new t	to identify aconceptual framework to assist systematic reviewers in the identification of data related to	yes	no	no	na	yes	table1
na	na	no	na	(1) to present and discuss implications of the	highlights the domains requi	yes	no	no	na	no	na
Framework for reporting	na	no	na	The purpose of this study w	to facilitate knowledge exch	yes	no	no	na	yes	Table 2
Standards for QUality Im	SQUIRE 2.0	no	na	This article describes the de	SQUIRE 2.0 is intended for re	yes	no	no	na	yes	Table 1
Guidelines for Reporting	GREOM	no	na	The aim of this study thus w	a set of guidelines we propos	yes	no	no	na	yes	Table 1
na	na	no	na	address the challenges of s	to improve the reporting of	yes	no	no	na	yes	Table 1
na	na	no	na	The purpose of this study w	conduct and reporting of ecc	yes	no	no	na	yes	Table 6
na	na	no	na	this article presents prelimi	we present a set of propositi	yes	no	no	na	yes	table 2 to 7
consolidated advice for r	C.A.R.E	no	na	develop and disseminate gl	to support a transparent anc	yes	no	no	na	yes	Table 1
na	na	no	na	Because there has been litt	This checklist identifies wha	yes	no	no	na	yes	table 1
Increasing the scale of pc	na	no	na	i) how decisions to scale up	describing a step-by-step prc	no	no	yes	na	yes	Table 1
Standards for Reporting I	StaRI	no	na	The Standards for Reporting	The StaRI Statesment and Ch	yes	no	no	na	yes	Table 1
Guide for Monitoring Sca	na	no	na	The main objective of this re	This guide is intended to prov	no	no	yes	na	no	na
na	na	no	na	The need for a common eva	presents such a frameworkin	no	no	yes	na	no	na
na	na	no	na	In this paper, the fourth of T	propose strategies to acceler	no	no	yes	na	yes	figure 2 page 4!
na	na	no	na	presents an evidence-based	It is intended to help you pro	no	no	yes	na	no	na
na	na	no	na	The aim of this paper is to i	to characterise the scaling u	no	no	yes	na	no	na
na	na	no	na	presenting recommendatio	This guide contains 12 recom	no	no	yes	na	yes	table page 12
mHealth Assessment anc	The MAPS Toc	no	na	to help project teams condu	to help project teams conduc	no	no	yes	na	yes	Figure 2
na	na	no	na	introduction to a series of iss	papers for FP/RH program managers that consider the following			yes	na	no	na
ExpandNet/WHO framev	na	no	na	drawing attention to insights from relevant literature found in a	thinking about the process of		no	yes	na	no	na
Multiplicative scale-up fr	na	no	na	provides general guidance f	steps are those recommende	no	no	yes	na	yes	box 6
framework for explaining	na	no	na	draw upon key themes in th	to use in thinking about strategies for implementing a new program, policy, or intervention to scale		no	yes	na	no	na
Scaling Up Management	SUM Framew	no	na	• To provide an easily unders	• To provide an easily understood and straightforward	no	no	yes	na	no	checklist scalab
AIDED model for scale u	na	na	na	To develop an integrated and p	To develop an integrated and practical	no	no	yes	na	yes	fig3
na	na	na	na	aimed to gather as comprel	how to take forwardthe proc	no	no	yes	na	no	na

Flowchart	Comment flowchart	Number of items	items id	Theoretical framework	name of the theory	Number of stakeholders involved in the project	Patient involved as stakeholder	Number of patient stakeholders	number of female patient stakeholder	number of male patient stakeholder	Clinician involved as stakeholders	Number of clinician stakeholders	number of female clinician stakeholders
no	na	20	1 - 20	no	na	32	no	na	na	na	no	na	na
no	na	31	21 - 51	no	na	11	no	na	na	na	no	na	na
yes	figure 3	4	52 - 55	no	na	3	no	na	na	na	no	na	na
no	na	5	56 - 60	no	na	2	no	na	na	na	no	na	na
no	na	58	61 - 118	no	na	26	no	na	na	na	no	na	na
no	na	21	119 - 139	no	na	3	no	na	na	na	yes	3	0
no	na	10	140 - 149	no	na	4	no	na	na	na	no	na	na
no	na	64	150 - 213	no	na	6	no	na	na	na	no	na	na
no	na	7	214 - 220	no	na	2	no	na	na	na	no	na	na
no	na	12	221 - 232	yes	framework for develc	16	no	na	na	na	no	na	na
no	na	19	233 - 251	no	na	1	no	na	na	na	no	na	na
no	na	47	252 - 298	no	na	6	no	na	na	na	no	na	na
no	na	17	299 - 315	no	na	5	no	na	na	na	no	na	na
no	na	32	316 - 347	no	na	5	no	na	na	na	no	na	na
yes	figure 1	30	348 - 377	no	na	23	no	na	na	na	yes	1	0
no	na	10	378 - 387	no	na	8	no	na	na	na	yes	2	2
no	na	40	388 - 427	no	na	39	no	na	na	na	yes	7	7
no	na	14	428 - 441	no	na	4	no	na	na	na	no	na	na
no	na	10	442 - 451	no	na	3	no	na	na	na	no	na	na
no	na	8	452 - 459	no	na	4	no	na	na	na	no	na	na
no	na	16	460 - 475	yes	Multiple Intervention:	7	no	na	na	na	no	na	na
no	na	21	476 - 496	no	na	4	no	na	na	na	no	na	na
no	na	20	497 - 516	no	na	na	na	na	na	na	na	na	na
no	na	20	517 - 536	no	na	8	no	na	na	na	no	na	na
no	na	37	537 - 573	no	na	14	no	na	na	na	unclear	na	na
no	na	10	574 - 583	no	na	8	no	na	na	na	no	na	na
yes	figure 2	7	584 - 590	no	na	5	no	na	na	na	no	na	na
no	na	4	591 - 594	no	na	17	no	na	na	na	yes	1	1
yes	figure 2	12	595 - 606	no	na	4	no	na	na	na	no	na	na
yes	fig,2	6	607 - 612	no	na	5	no	na	na	na	no	na	na
no	na	12	613 - 624	yes	ExpandNet/WHO fran	3	no	na	na	na	no	na	na
no	na	38	625 - 662	no	na	13	no	na	na	na	no	na	na
no	na	10	663 - 672	no	na	na	na	na	na	na	na	na	na
yes	fig1	27	673 - 699	no	na	3	no	na	na	na	no	na	na
no	na	17	700 - 716	yes	BTS collaborative lear	na	na	na	na	na	na	na	na
no	na	6	717 - 722	no	na	1	no	na	na	na	no	na	na
no	na	14	723 - 736	yes	strategic managemen	3	no	na	na	na	no	na	na
no	na	5	737-741	no	na	11	no	na	na	na	no	na	na
yes	figure 3	9	742-750	no	na	9	no	na	na	na	yes	1	0

number of male clinician stakeholders	Decision or policy maker involved as stakeholders	Number of decision or policy maker involved as stakeholders	number of female decision/policy maker stakeholders	number of male decision/policy maker stakeholders	Journal editor involved as stakeholder	Number of journal editor involved as stakeholder	number of female journal editor stakeholder	number of male Journal editor stakeholder	Funding organisation member as stakeholder	Number of funding organisation member stakeholder	Number of female stakeholder from funding organization	Number of male stakeholder from funding organization
na	no	na	na	na	yes	2	2	0	no	na	na	na
na	no	na	na	na	no	na	na	na	yes	1	1	0
na	no	na	na	na	no	na	na	na	no	na	na	na
na	no	na	na	na	no	na	na	na	no	na	na	na
na	yes	6	4	2	yes	17	0	17	no	na	na	na
3	no	na	na	na	no	na	na	na	no	na	na	na
na	no	na	na	na	no	na	na	na	no	na	na	na
na	yes	6	1	5	no	na	na	na	no	na	na	na
na	no	na	na	na	no	na	na	na	no	na	na	na
na	no	na	na	na	yes	2	2	0	no	na	na	na
na	no	na	na	na	yes	1	1	0	no	na	na	na
na	yes	5	2	3	no	na	na	na	no	na	na	na
na	no	na	na	na	no	na	na	na	no	na	na	na
na	no	na	na	na	no	na	na	na	no	na	na	na
1	yes	5	4	1	no	na	na	na	no	na	na	na
0	no	na	na	na	no	na	na	na	no	na	na	na
0	yes	4	2	2	yes	1	0	1	no	na	na	na
na	no	na	na	na	no	na	na	na	no	na	na	na
na	no	na	na	na	no	na	na	na	no	na	na	na
na	no	na	na	na	no	na	na	na	no	na	na	na
na	no	na	na	na	no	na	na	na	no	na	na	na
na	no	na	na	na	no	na	na	na	no	na	na	na
na	na	na	na	na	na	na	na	na	na	na	na	na
na	no	na	na	na	no	na	na	na	no	na	na	na
na	no	na	na	na	no	na	na	na	yes	2	2	0
na	yes	na	na	na	no	na	na	na	yes	na	na	na
na	yes	1	0	1	no	na	na	na	no	na	na	na
0	yes	11	8	3	no	na	na	na	yes	1	0	1
na	yes	1	0	1	no	na	na	na	yes	3	1	2
na	yes	1	0	1	no	na	na	na	no	na	na	na
na	yes	1	1	0	no	na	na	na	yes	1	0	1
na	yes	7	5	2	no	na	na	na	yes	2	1	1
na	na	na	na	na	na	na	na	na	na	na	na	na
na	yes	1	1	0	no	na	na	na	yes	1	0	1
na	na	na	na	na	na	na	na	na	na	na	na	na
na	no	na	na	na	no	na	na	na	no	na	na	na
na	yes	3	1	2	no	na	na	na	no	na	na	na
na	no	na	na	na	no	na	na	na	no	na	na	na
1	yes	3	2	1	no	na	na	na	no	na	na	na

researchers involved as stakeholders	Number of researcher involved in the project	number of female researcher stakeholder	number of male researcher stakeholder	consensus	Delphi	informal consensus method	nominal group technique	Rand UCLA appropriateness Method	consensus conference	type of consensus other	electronic features	face to face meeting	panelist	Number of panelists
yes	30	7	23	yes	no	no	no	no	yes	no	unclear	yes	unclear	na
yes	10	4	6	unclear	na	na	na	na	na	na	na	na	na	na
yes	3	2	1	no	na	na	na	na	na	na	na	na	na	na
yes	2	2	0	no	na	na	na	na	na	na	na	na	na	na
yes	3	0	3	yes	no	no	no	no	yes	no	no	yes	yes	26
no	na	na	na	no	na	na	na	na	na	na	na	na	na	na
yes	4	2	2	no	na	na	na	na	na	na	na	na	na	na
no	0	0	0	yes	yes	no	no	no	no	no	yes	yes	yes	44
yes	2	1	1	unclear	na	na	na	na	na	na	na	na	na	na
yes	14	8	6	yes	yes	no	no	no	no	no	yes	yes	yes	90
no	0	0	0	no	na	na	na	na	na	na	na	na	na	na
yes	1	1	0	yes	yes	no	no	no	no	no	yes	yes	yes	48
yes	5	4	1	yes	no	yes	no	no	no	na	no	yes	yes	17
yes	5	2	3	yes	no	yes	no	no	no	na	unclear	unclear	no	na
yes	17	7	10	yes	no	yes	no	no	no	no	yes	yes	yes	23
yes	6	6	0	yes	no	no	yes	no	yes	no	yes	yes	yes	na
yes	27	10	17	yes	no	no	no	no	yes	no	yes	yes	yes	na
yes	4	3	1	yes	no	yes	no	no	no	no	no	yes	yes	na
yes	3	1	2	no	na	na	na	na	na	na	na	na	na	na
yes	4	2	2	no	na	na	na	na	na	na	na	na	na	na
yes	7	7	0	no	na	na	na	na	na	na	na	na	na	na
yes	4	4	0	yes	yes	no	no	no	no	no	yes	yes	yes	14
na	na	na	na	na	na	na	na	na	na	na	na	na	na	na
yes	8	3	5	yes	yes	no	yes	no	no	no	no	yes	yes	na
yes	12	7	5	yes	yes	no	no	no	no	no	yes	yes	yes	15
na	na	na	na	no	na	na	na	na	na	na	na	na	na	na
yes	4	1	3	no	na	na	na	na	na	na	na	na	na	na
yes	4	2	2	yes	no	yes	no	no	no	na	no	yes	yes	na
no	0	0	0	yes	no	yes	no	no	no	no	yes	yes	yes	na
yes	4	3	1	no	na	na	na	na	na	na	na	na	na	na
yes	1	1	0	no	na	na	na	na	na	na	na	na	na	na
na	3	2	1	yes	no	yes	no	no	no	na	na	yes	yes	na
na	na	na	na	na	na	na	na	na	na	na	na	na	na	na
yes	1	1	0	yes	no	yes	no	no	no	na	na	yes	yes	na
na	na	na	na	na	na	na	na	na	na	na	na	na	na	na
yes	1	0	1	no	na	na	na	na	na	na	na	na	na	na
no	0	0	0	no	na	na	na	na	na	na	na	na	na	na
yes	11	9	2	no	na	na	na	na	na	na	na	na	na	na
yes	5	3	2	no	na	na	na	na	na	na	na	na	na	na

patient as panelist	number of patient as panelist	number of female patient as panelist	number of male patient as panelist	clinician as panelist	number of clinician as panelist	number of female clinicians as panelist	number of male clinicians as panelist	researcher as panelist	Number of researchers as panelist	Number of female researchers as panelist	Number of male researchers as panelist	funders as panelist	number of funders as panelist	number of female funders as panelist
na	na	na	na	na	na	na	na	na	na	na	na	na	na	na
na	na	na	na	na	na	na	na	na	na	na	na	na	na	na
na	na	na	na	na	na	na	na	na	na	na	na	na	na	na
na	na	na	na	na	na	na	na	na	na	na	na	na	na	na
na	na	na	na	no	na	na	na	yes	3	0	3	no	na	na
na	na	na	na	na	na	na	na	na	na	na	na	na	na	na
na	na	na	na	na	na	na	na	na	na	na	na	na	na	na
unclear	na	na	na	unclear	na	na	na	yes	na	na	na	yes	na	na
na	na	na	na	na	na	na	na	na	na	na	na	na	na	na
no	na	na	na	yes	28	na	na	yes	39	na	na	no	na	na
na	na	na	na	na	na	na	na	na	na	na	na	na	na	na
no	na	na	na	yes	na	na	na	yes	na	na	na	yes	na	na
no	na	na	na	yes	1	0	1	yes	5	1	4	yes	1	0
na	na	na	na	na	na	na	na	na	na	na	na	na	na	na
no	na	na	na	yes	1	0	1	yes	17	7	10	no	na	na
yes	na	na	na	yes	na	na	na	yes	na	na	na	na	na	na
no	na	na	na	no	na	na	na	yes	na	na	na	no	na	na
na	na	na	na	na	na	na	na	na	na	na	na	na	na	na
na	na	na	na	na	na	na	na	na	na	na	na	na	na	na
na	na	na	na	na	na	na	na	na	na	na	na	na	na	na
na	na	na	na	na	na	na	na	na	na	na	na	na	na	na
no	0	0	0	no	na	na	na	yes	13	10	3	no	na	na
na	na	na	na	na	na	na	na	na	na	na	na	na	na	na
no	na	na	na	yes	na	na	na	yes	na	na	na	no	na	na
unclear	na	na	na	unclear	na	na	na	yes	9	unclear	unclear	yes	2	na
na	na	na	na	na	na	na	na	na	na	na	na	na	na	na
na	na	na	na	na	na	na	na	na	na	na	na	na	na	na
no	na	na	na	yes	na	na	na	na	na	na	na	yes	na	na
unclear	na	na	na	unclear	na	na	na	yes	na	na	na	yes	na	na
na	na	na	na	na	na	na	na	na	na	na	na	na	na	na
na	na	na	na	na	na	na	na	na	na	na	na	na	na	na
na	na	na	na	na	na	na	na	yes	na	na	na	yes	na	na
na	na	na	na	na	na	na	na	na	na	na	na	na	na	na
no	na	na	na	unclear	na	na	na	yes	na	na	na	no	na	na
na	na	na	na	na	na	na	na	na	na	na	na	na	na	na
na	na	na	na	na	na	na	na	na	na	na	na	na	na	na
na	na	na	na	na	na	na	na	na	na	na	na	na	na	na
na	nana	na	na	na	na	na	na	na	na	na	na	na	na	na
na	na	na	na	na	na	na	na	na	na	na	na	na	na	na

number of male funders as panelist	Decision Maker as panelist	Number of DM as panelist	Number of female DM as panelist	Number of male DM as panelist	Journal editor as panelist	Number of Journal editor as panelist	Number of female journal editor as panelist	Number of male journal editor as panelist	systematic review	litterature review	observational study	qualitative research study	randomize d trial	Type of data source other	presence of sex related word
na	no	na	na	na	no	na	na	na	yes	no	no	no	no	na	no
na	na	na	na	na	na	na	na	na	no	unclear	no	no	no	na	no
na	na	na	na	na	na	na	na	na	no	yes	no	no	no	na	no
na	na	na	na	na	na	na	na	na	no	yes	no	no	no	na	no
na	yes	6	4	2	yes	17	0	17	no	yes	no	no	no	no	no
na	na	na	na	na	na	na	na	na	na	no	no	no	no	no	no
na	na	na	na	na	na	na	na	na	yes	no	no	no	no	no	no
na	unclear	na	na	na	yes	na	na	na	no	yes	no	no	no	no	no
na	na	na	na	na	na	na	na	na	no	yes	no	no	no	no	no
na	no	na	na	na	yes	12	na	na	no	yes	no	no	no	no	no
na	na	na	na	na	na	na	na	na	no	no	no	no	no	no	no
na	yes	na	na	na	unclear	na	na	na	yes	no	no	no	no	na	no
1	yes	11	2	9	no	na	na	na	no	no	no	no	no	Expert opinion	no
na	na	na	na	na	na	na	na	na	yes	no	no	no	no	no	no
na	yes	5	4	1	no	na	na	na	no	no	no	no	no	Expert opinion	no
na	no	na	na	na	no	na	na	na	yes	no	no	no	no	no	no
na	yes	na	na	na	yes	na	na	na	no	yes	no	no	no	na	no
na	na	na	na	na	na	na	na	na	no	yes	no	no	no	na	no
na	na	na	na	na	na	na	na	na	no	no	no	no	no	no	no
na	na	na	na	na	na	na	na	na	yes	no	no	no	no	na	yes
na	na	na	na	na	na	na	na	na	no	yes	no	no	no	no	no
na	yes	1	1	0	no	na	na	na	no	yes	no	no	no	no	no
na	na	na	na	na	na	na	na	na	no	yes	no	no	no	no	no
na	yes	na	na	na	no	na	na	na	yes	no	no	yes	no	na	no
na	no	na	na	na	yes	6	na	na	yes	no	no	no	no	no	no
na	na	na	na	na	na	na	na	na	na	na	na	na	na	na	no
na	na	na	na	na	na	na	na	na	no	yes	no	no	no	na	no
na	yes	na	na	na	na	na	na	na	yes	no	no	no	no	na	no
na	yes	na	na	na	unclear	na	na	na	no	yes	no	no	no	na	no
na	na	na	na	na	na	na	na	na	no	yes	no	no	no	na	no
na	na	na	na	na	na	na	na	na	na	yes	na	na	na	field experienc	no
na	yes	na	na	na	na	na	na	na	na	yes	no	yes	no	Expert opinion	no
na	na	na	na	na	na	na	na	na	na	na	na	na	na	na	no
na	yes	na	na	na	no	na	na	na	no	yes	no	no	no	interview	no
na	na	na	na	na	na	na	na	na	na	na	na	na	na	na	no
na	na	na	na	na	na	na	na	na	no	yes	no	no	no	interview	no
na	na	na	na	na	na	na	na	na	na	na	na	na	na	field work	no
na	na	na	na	na	na	na	na	na	yes	no	no	yes	no	no	no
na	na	na	na	na	na	na	na	na	yes	no	no	no	no	survey	no

sex info	correct sex	presence of gender related word	gender info	correct gender	Validation	type of validation	funding source	funding source detail	Funding source classification	conflict of interest	presence of conflict of interest
na	na	no	na	na	no	na	yes	[illegible] KRS 102071)	public funding	yes	no
na	na	no	na	na	no	na	yes	national Institutes of Health (RO1 DK5	public funding	no	na
na	na	no	na	na	yes	testing	yes	funding from Kaiser Permanente	private funding	yes	no
na	na	no	na	na	no	na	yes	national Institutes of Health (R01NR0	public funding	no	na
na	na	no	na	na	no	na	no	na	na	no	na
na	na	no	na	na	no	na	no	na	na	no	na
na	na	no	na	na	yes	pilot testing	yes	ESRC grant no. H141251011 (as part of the ESRC Centre for Evidence-	public funding	yes	no
na	na	no	na	na	yes	pilot testing	yes	Special Programme for Research and	public funding	yes	no
na	na	no	na	na	yes	expert feedback	no	na	na	yes	no
na	na	no	na	na	yes	pilot testing	yes	NIHR Senior Investigator Award; Aust	private & public funding	yes	no
na	na	no	na	na	no	na	no	na	na	no	na
na	na	yes	item 259, 26	unclear	yes	pilot testing	yes	the Alliance for Health Policy and Syst	public funding	yes	no
na	na	no	na	na	yes	pilot testing	yes	PGS. Administrative, technical or mat	public funding	yes	no
na	na	no	na	na	no	na	yes	the Brown University Alcohol Resear	public funding	yes	no
na	na	no	na	na	no	na	yes	This work was partially funded by the	public funding	no	na
na	na	no	na	na	no	na	yes	the Physiotherapy Foundation of Canada through the Alberta	private & public funding	yes	no
na	na	no	na	na	yes	pilot test & expert feedl	yes	Robert Wood Johnson Foundation (gr	private funding	yes	no
na	na	no	na	na	no	na	yes	Grant DEP2012- 32124]; 2) the Gener	public funding	no	na
na	na	no	na	na	no	na	yes	national Center for Research Resources through the Dissemination and Implementation Research Core of Washingtor	public funding	yes	no
item 454	unclear	no	na	na	no	na	yes	none	na	yes	no
na	na	no	na	na	no	na	yes	Contributions were supported by awa	private & public funding	yes	no
na	na	no	na	na	no	na	yes	New York Academy of Sciences UNI(	public funding	yes	no
na	na	no	na	na	no	na	no	na	na	no	na
na	na	no	na	na	no	na	yes	New South Wales Ministry of Health (	public funding	yes	no
na	na	no	na	na	no	na	yes	Asthma UK Centre for Applied Resear	public funding	yes	no
na	na	no	na	na	no	na	no	na	na	no	na
na	na	no	na	na	no	na	yes	Canadian CIDA and the Bill & Melinda	private & public funding	yes	no
na	na	no	na	na	yes	pilot testing	yes	grant from USAID to UNICEF, and fror	private & public funding	yes	no
na	na	no	na	na	no	na	yes	International Development R esearch	public funding	no	na
na	na	no	na	na	no	na	yes	the national Health and Medical Research Council of Australia (NHMRC) through its partnership centre grant scheme	public funding	yes	no
na	na	yes	item 616	yes	no	na	yes	Financial support from the David and	private & public funding	no	na
na	na	no	na	na	yes	pilot test, expert feedba	yes	WHO and UNF are grateful to Helga F	private & public funding	no	na
na	na	yes	item 666	unclear	no	na	no	na	na	no	na
na	na	no	na	na	no	na	yes	the financial support received from tl	private & public funding	no	na
na	na	no	na	na	no	na	no	na	na	no	na
na	na	no	na	na	no	na	yes	no funding was received for this work	na	yes	yes
na	na	no	na	na	no	na	yes	generous support from the John D an	private funding	no	na
na	na	no	na	na	no	na	yes	Bill and Melinda Gates Foundation, gr	private funding	yes	no
na	na	no	na	na	no	na	no	na	na	yes	no
