# Supplementary material for: Informing the development of the SUCCEED reporting guideline for studies on the scaling of health interventions: A systematic review
Source: Medicine (Baltimore). 2024 Feb 16;103(7):e37079. doi: 10.1097/MD.0000000000037079 (PMC10869056; doi:10.1097/MD.0000000000037079)
Supplement: Supplementary file 5 [file medi-103-e37079-s005.docx]

Supplementary file 5. Sex of the authors of the included guidelines

| Name of guidelines^1^  (Year) ^reference^ | Sex of first author^2^ |
| --- | --- |
| Reach, efficacy, adoption, implementation, and maintenance framework (1999) ^26^ | M |
| USAID and Management Sciences for Health (2002) ^28^ | NA |
| Reviewer Guidelines for Reports of Public Health Interventions (2003) ^29^ | F |
| Transparent Reporting of Evaluations with nonrandomized Designs (2004) ^30^ | M |
| ExpandNet/WHO framework for scaling up (2007) ^8^ | F |
| Riley et al. (2008) ^34^ | F |
| Egan et al. (2009) ^35^ | M |
| Framework for reporting health service delivery models for managing rheumatoid arthritis (2010) ^36^ | F |
| Bryce et al. (2011) ^38^ | F |
| Conn & Groves (2011) ^39^ | F |
| Eaton et al (2011) ^40^ | M |
| WHO/ExpandNet (2011) ^41^ | NA |
| Framework for explaining successful scale-up (2011) ^42^ | M |
| AIDED model for scale-up (2012) ^43^ | F |
| Reporting standards for studies of tailored interventions (2012) ^44^ | F |
| Guide for Monitoring Scale-up of Health Practices and Interventions (2013) ^45^ | F |
| Workgroup for Intervention Development and Evaluation Research  recommendations (2013) ^46^ | F |
| Duncan et al. (2013) ^48^ | M |
| The Oxford Implementation Index (2013) ^49^ | M |
| Proctor et al. (2013) ^50^ | F |
| Dickson et al (2014) ^51^ | F |
| Template for intervention description and replication (2014) ^52^ | F |
| Global framework implementation criteria for pilot test (2014) ^53^ | F |
| Multiplicative scale-up framework (2014) ^54^ | NA |
| mHealth Assessment and Planning for Scale (2015) ^55^ | NA |
| Neta et al. (2015) ^56^ | F |
| Guidelines for Reporting Evaluations based on Observational Methodology (2015) ^57^ | F |
| CCDR (2016) ^58^ | NA |
| Barker et al. (2016) ^59^ | M |
| Scaling Up Management Framework (2016) ^60^ | M |
| Hales et al. (2016) ^61^ | M |
| Milat et al. (2016) ^6^ | M |
| Standards for QUality Improvement Reporting Excellence (2016) ^66^ | M |
| Indig et al. (2017) ^71^ | F |
| Programme Reporting Standards (2017) ^72^ | F |
| Standards for Reporting Implementation Studies (2017) ^73^ | F |
| Consolidated advice for reporting ECD implementation research (2018) ^76^ | F |
| McLean & Gargani (2019) ^11^ | M |
| Reeves et al. (2019) ^78^ | F |

^1^Authors or organizations if no name was identified

^2^F: female, M: male, NA: no author identified or organization
