# Supplementary material for: Informing the development of the SUCCEED reporting guideline for studies on the scaling of health interventions: A systematic review
Source: Medicine (Baltimore). 2024 Feb 16;103(7):e37079. doi: 10.1097/MD.0000000000037079 (PMC10869056; doi:10.1097/MD.0000000000037079)
Supplement: Supplementary file 6 [file medi-103-e37079-s006.docx]

Supplementary file 6. Stakeholder groups (authors) involved in the development of the included guidelines

| Stakeholder group | Designing scaling interventions  N = 17 (%) | Reporting implementation interventions  N = 22 (%) | Total  N = 39 (%) |
| --- | --- | --- | --- |
| Clinician  *Yes*  *No*  *Unclear*  *NA* | 2 (12)  13 (77)  0 (0)  2 (12) | 4 (18)  16 (73)  1 (5)  1 (5) | 6 (15)  29 (74)  1 (3)  3 (8) |
| Decision maker  *Yes*  *No*  *Unclear*  *NA* | 10 (59)  5 (29)  0 (0)  2 (12) | 5 (23)  16 (73)  0 (0)  1 (5) | 15 (39)  21 (54)  0 (0)  3 (8) |
| Editor/publisher  *Yes*  *No*  *Unclear*  *NA* | 0 (0)  15 (88)  0 (0)  2 (12) | 5 (23)  16 (73)  0 (0)  1 (5) | 5 (13)  31 (80)  0 (0)  3 (8) |
| Funder  *Yes*  *No*  *Unclear*  *NA* | 7 (41)  8 (47)  0 (0)  2 (12) | 1 (5)  20 (91)  0 (0)  1 (5) | 8 (21)  28 (72)  0 (0)  3 (8) |
| Patient  *Yes*  *No*  *Unclear*  *NA* | 0 (0)  15 (88)  0 (0)  2 (12) | 0 (0)  21 (95)  0 (0)  1 (5) | 0 (0)  36 (92)  0 (0)  3 (8) |
| Researcher  *Yes*  *No*  *Unclear*  *NA* | 11 (65)  2 (12)  0 (0)  4 (24) | 18 (82)  3 (14)  0 (0)  1 (5) | 29 (74)  5 (13)  0 (0)  5 (13) |

NA : no author identified or organization
