# Supplementary material for: Informing the development of the SUCCEED reporting guideline for studies on the scaling of health interventions: A systematic review
Source: Medicine (Baltimore). 2024 Feb 16;103(7):e37079. doi: 10.1097/MD.0000000000037079 (PMC10869056; doi:10.1097/MD.0000000000037079)
Supplement: Supplementary file 7 [file medi-103-e37079-s007.docx]

Supplementary file 7. Panelist groups involved in the consensus process of the included guidelines

| Panelist group | Designing scaling interventions  N = 17 (%) | Reporting implementation interventions  N = 22 (%) | Total  N = 39 (%) |
| --- | --- | --- | --- |
| Clinician  *Yes*  *No*  *Unclear*  *NA* | 2 (13)  0 (0)  2 (13)  11 (73) | 5 (23)  3 (14)  2 (9)  12 (55) | 7 (19)  3 (8)  4 (11)  23 (62) |
| Decision maker  *Yes*  *No*  *Unclear*  *NA* | 5 (33)  0 (0)  0 (0)  10 (67) | 6 (27)  4 (18)  1 (5)  11 (50) | 11 (30)  4 (11)  1 (3)  21 (57) |
| Editor/publisher  *Yes*  *No*  *Unclear*  *NA* | 0 (0)  2 (13)  1 (7)  12 (80) | 5 (23)  5 (23)  1 (5)  11 (50) | 5 (14)  7 (19)  2 (5)  23 (62) |
| Funder  *Yes*  *No*  *Unclear*  *NA* | 3 (20)  2 (13)  0 (0)  10 (76) | 4 (18)  5 (23)  0 (0)  13 (59) | 7 (19)  7 (19)  0 (0)  23 (62) |
| Patient  *Yes*  *No*  *Unclear*  *NA* | 0 (0)  3 (20)  1 (7)  11 (73) | 1 (5)  6 (27)  2 (9)  13 (59) | 1 (3)  9 (24)  3 (8)  24 (65) |
| Researcher  *Yes*  *No*  *Unclear*  *NA* | 4 (27)  0 (0)  0 (0)  11 (73) | 10 (45)  0 (0)  0 (0)  12 (55) | 14 (38)  0 (0)  0 (0)  23 (26) |

NA : no panel group identified
