# Supplementary material for: Informing the development of the SUCCEED reporting guideline for studies on the scaling of health interventions: A systematic review
Source: Medicine (Baltimore). 2024 Feb 16;103(7):e37079. doi: 10.1097/MD.0000000000037079 (PMC10869056; doi:10.1097/MD.0000000000037079)
Supplement: Supplementary file 8 [file medi-103-e37079-s008.docx]

**S8 Figure**: Trends in the evidence-based development of included guidelines

Based on a 3-item internal validity assessment tool (high if ≥ 2 “yes” and low if < 2 “yes”)
