# Supplementary material for: Informing the development of the SUCCEED reporting guideline for studies on the scaling of health interventions: A systematic review
Source: Medicine (Baltimore). 2024 Feb 16;103(7):e37079. doi: 10.1097/MD.0000000000037079 (PMC10869056; doi:10.1097/MD.0000000000037079)
Supplement: Supplementary file 9 [file medi-103-e37079-s009.pdf]

Items id	study_Id	Study title	author	Items	Description	dimension covered	format	location	Title	abstract	introduction	method	results	discussion	other information
1	s1	Development of a checklist to assess the (	Albrecht, Lauren	Detailed description of interventions in published	na	na	list	Table 1	no	yes	no	yes	yes	no	no
2	s1	Development of a checklist to assess the (	Albrecht, Lauren	Detailed description of interventions in published	na	na	list	Table 1	no	yes	no	yes	yes	no	no
3	s1	Development of a checklist to assess the (	Albrecht, Lauren	Detailed description of interventions in published	na	na	list	Table 1	yes	yes	yes	yes	yes	no	no
4	s1	Development of a checklist to assess the (	Albrecht, Lauren	Detailed description of interventions in published	na	na	list	Table 1	no	yes	no	yes	no	no	no
5	s1	Development of a checklist to assess the (	Albrecht, Lauren	Detailed description of interventions in published	na	na	list	Table 1	no	yes	no	yes	yes	no	no
6	s1	Development of a checklist to assess the (	Albrecht, Lauren	Detailed description of interventions in published	na	na	list	Table 1	no	yes	no	yes	yes	no	no
7	s1	Development of a checklist to assess the (	Albrecht, Lauren	Detailed description of interventions in published	na	na	list	Table 1	no	yes	no	yes	yes	no	no
8	s1	Development of a checklist to assess the (	Albrecht, Lauren	Detailed description of interventions in published	na	na	list	Table 1	yes	yes	yes	no	yes	no	no
9	S1	Development of a checklist to assess the (	Albrecht, Lauren	Clarification of assumed change process and desi	na	na	list	Table 1	yes	yes	yes	no	yes	no	no
10	s1	Development of a checklist to assess the (	Albrecht, Lauren	Clarification of assumed change process and desi	na	na	list	Table 1	yes	yes	yes	no	yes	no	no
11	s1	Development of a checklist to assess the (	Albrecht, Lauren	Clarification of assumed change process and desi	na	na	list	Table 1	yes	yes	yes	yes	yes	yes	no
12	s1	Development of a checklist to assess the (	Albrecht, Lauren	Access to intervention manuals/protocols:	na	na	list	Table 1	no	no	yes	yes	no	no	no
13	s1	Development of a checklist to assess the (	Albrecht, Lauren	Detailed description of active control conditions:	na	na	list	Table 1	no	yes	no	yes	yes	no	no
14	s1	Development of a checklist to assess the (	Albrecht, Lauren	Detailed description of active control conditions:	na	na	list	Table 1	no	yes	no	yes	yes	no	no
15	s1	Development of a checklist to assess the (	Albrecht, Lauren	Detailed description of active control conditions:	na	na	list	Table 1	yes	yes	yes	yes	yes	yes	no
16	s1	Development of a checklist to assess the (	Albrecht, Lauren	Detailed description of active control conditions:	na	na	list	Table 1	yes	yes	yes	yes	yes	yes	no
17	s1	Development of a checklist to assess the (	Albrecht, Lauren	Detailed description of active control conditions:	na	na	list	Table 1	no	no	yes	yes	yes	yes	no
18	s1	Development of a checklist to assess the (	Albrecht, Lauren	Detailed description of active control conditions:	na	na	list	Table 1	yes	yes	yes	yes	yes	yes	no
19	s1	Development of a checklist to assess the (	Albrecht, Lauren	Detailed description of active control conditions:	na	na	list	Table 1	no	no	no	yes	yes	yes	no
20	s1	Development of a checklist to assess the (	Albrecht, Lauren	Detailed description of active control conditions:	na	na	list	Table 1	yes	yes	yes	yes	yes	yes	no
21	s2	Evaluating the public health impact of hea	Glasgow, Russell E	Reach:Exclusion Criteria	% excluded or characteristics	Evaluation and monitoring	list	Checklist website	no	no	no	yes	no	no	no
22	s2	Evaluating the public health impact of hea	Glasgow, Russell E	Reach:Percent individuals who participate, based	not of volunteers who indicate inte	Evaluation and monitoring	list	Checklist website	no	no	no	yes	yes	no	no
23	s2	Evaluating the public health impact of hea	Glasgow, Russell E	Reach:Characteristics of participants compared t	na	Evaluation and monitoring	list	Checklist website	no	no	no	no	no	no	no
24	s2	Evaluating the public health impact of hea	Glasgow, Russell E	Reach:Use of qualitative methods to understand	na	Evaluation and monitoring	list	Checklist website	no	yes	no	yes	no	no	no
25	s2	Evaluating the public health impact of hea	Glasgow, Russell E	Effectiveness: Measure of primary outcome with	e.g. HP 2020 goals, exercise 30 min,	Evaluation and monitoring	list	Checklist website	no	yes	no	yes	yes	no	no
26	s2	Evaluating the public health impact of hea	Glasgow, Russell E	Effectiveness: Measure of broader outcomes	e.g., other outcomes, measure of Q	Evaluation and monitoring	list	Checklist website	no	no	no	yes	yes	no	no
27	s2	Evaluating the public health impact of hea	Glasgow, Russell E	Effectiveness: Measure of robustness across subg	e.g. moderation analyses	Evaluation and monitoring	list	Checklist website	no	no	no	yes	yes	no	no
28	s2	Evaluating the public health impact of hea	Glasgow, Russell E	Effectiveness: Measure of short-term attrition (%	%) and differential rates by patient	Evaluation and monitoring	list	Checklist website	no	no	no	yes	yes	no	no
29	s2	Evaluating the public health impact of hea	Glasgow, Russell E	Effectiveness: Use of qualitative methods/data to	na	Evaluation and monitoring	list	Checklist website	no	no	no	yes	no	no	no
30	s2	Evaluating the public health impact of hea	Glasgow, Russell E	Adoption – Setting LevelSetting Exclusions	% or reasons	Evaluation and monitoring	list	Checklist website	no	no	no	no	no	no	no
31	s2	Evaluating the public health impact of hea	Glasgow, Russell E	Adoption – Setting LevelPercent of settings appro	valid denominator	Evaluation and monitoring	list	Checklist website	no	no	no	no	no	no	no
32	s2	Evaluating the public health impact of hea	Glasgow, Russell E	Adoption – Setting LevelCharacteristics of setting	na	Evaluation and monitoring	list	Checklist website	no	no	no	yes	yes	no	no
33	s2	Evaluating the public health impact of hea	Glasgow, Russell E	Adoption – Setting LevelUse of qualitative metho	na	Evaluation and monitoring	list	Checklist website	no	no	no	yes	no	no	no
34	s2	Evaluating the public health impact of hea	Glasgow, Russell E	Adoption – Staff LevelStaff Exclusions	% or reasons	Evaluation and monitoring	list	Checklist website	no	no	no	yes	yes	no	no
35	s2	Evaluating the public health impact of hea	Glasgow, Russell E	Adoption – Staff LevelPercent of staff invited that	na	Evaluation and monitoring	list	Checklist website	no	no	no	yes	yes	no	no
36	s2	Evaluating the public health impact of hea	Glasgow, Russell E	Adoption – Staff LevelCharacteristics of staff part	na	Evaluation and monitoring	list	Checklist website	no	no	no	yes	yes	no	no
37	s2	Evaluating the public health impact of hea	Glasgow, Russell E	Adoption – Staff LevelUse of qualitative methods	na	Evaluation and monitoring	list	Checklist website	no	no	no	no	no	no	no
38	s2	Evaluating the public health impact of hea	Glasgow, Russell E	ImplementationPercent of perfect delivery or cal	e.g., adherence or consistency)	Evaluation and monitoring	list	Checklist website	no	no	no	yes	yes	no	no
39	s2	Evaluating the public health impact of hea	Glasgow, Russell E	ImplementationAdaptations made to interventio	na	Evaluation and monitoring	list	Checklist website	no	no	no	yes	yes	yes	no
40	s2	Evaluating the public health impact of hea	Glasgow, Russell E	ImplementationCost of intervention	time or money	Evaluation and monitoring	list	Checklist website	no	no	no	yes	yes	no	no
41	s2	Evaluating the public health impact of hea	Glasgow, Russell E	ImplementationConsistency of implementation a	not about differential outcomes, bu	Evaluation and monitoring	list	Checklist website	no	no	no	yes	yes	no	no
42	s2	Evaluating the public health impact of hea	Glasgow, Russell E	ImplementationUse of qualitative methods to un	na	Evaluation and monitoring	list	Checklist website	no	no	no	yes	no	no	no
43	s2	Evaluating the public health impact of hea	Glasgow, Russell E	Maintenance – Individual LevelMeasure of prima	with or w/o comparison to a public	Evaluation and monitoring	list	Checklist website	no	yes	no	yes	no	no	no
44	s2	Evaluating the public health impact of hea	Glasgow, Russell E	Maintenance – Individual LevelMeasure of broad	e.g., measure of QoL or potential ne	Evaluation and monitoring	list	Checklist website	no	no	no	yes	no	no	no
45	s2	Evaluating the public health impact of hea	Glasgow, Russell E	Maintenance – Individual LevelRobustness data -	na	Evaluation and monitoring	list	Checklist website	no	no	no	yes	yes	no	no
46	s2	Evaluating the public health impact of hea	Glasgow, Russell E	Maintenance – Individual LevelMeasure of long-t	na	Evaluation and monitoring	list	Checklist website	no	no	no	yes	yes	no	no
47	s2	Evaluating the public health impact of hea	Glasgow, Russell E	Maintenance – Individual LevelUse of qualitative	na	Evaluation and monitoring	list	Checklist website	no	no	no	yes	no	no	no
48	s2	Evaluating the public health impact of hea	Glasgow, Russell E	Maintenance- Setting LevelIf program is still ongo	na	Evaluation and monitoring	list	Checklist website	no	no	no	yes	yes	no	no
49	s2	Evaluating the public health impact of hea	Glasgow, Russell E	Maintenance- Setting LevelIf and how program w	which elements retained AFTER pro	Evaluation and monitoring	list	Checklist website	no	no	no	yes	no	no	no
50	s2	Evaluating the public health impact of hea	Glasgow, Russell E	Maintenance- Setting LevelSome measure/discus	na	Evaluation and monitoring	list	Checklist website	no	no	no	yes	no	yes	no
51	s2	Evaluating the public health impact of hea	Glasgow, Russell E	Maintenance- Setting LevelUse of qualitative met	na	Evaluation and monitoring	list	Checklist website	no	no	no	yes	no	no	no
52	s3	A framework for scaling up health interve	Barker, PM	Setup	This phase establishes an entry poir	preparing for scaling up	Narrative	Page 5	no	no	no	yes	no	no	no
53	s3	A framework for scaling up health interve	Barker, PM	Develop the scalable unit	This phase develops the "scalable u	preparing for scaling up	Narrative	Page 5	no	no	no	yes	no	no	no
54	s3	A framework for scaling up health interve	Barker, PM	Test of scale-up (i.e., testing the set of interventions to be	The underlying theory of change an	Assessment of scalability of th	Narrative	Page 6	no	no	no	yes	no	no	no
55	s3	A framework for scaling up health interve	Barker, PM	Go to full scale	This is a rapid deployment phase in	Implementation of the strate	Narrative	Page 6	no	no	no	no	no	no	no
56	s4	Protecting the power of interventions thr	Vicki S	Theory: Why was a particular intervention (or set	Explicitly identify the theory• Link the intervention attributes to theoretical concepts	na	list	Table 1	no	yes	yes	yes	no	no	no
57	s4	Protecting the power of interventions thr	Vicki S	Intervention Recipient: Who received the interve	Differentiate between the intervention target and recipient	na	list	Table 1	yes	yes	no	yes	no	no	no
58	s4	Protecting the power of interventions thr	Vicki S	Interventionist: Who delivered the intervention t	Identify the interventionist and any relationship to the recipient	na	list	Table 1	no	yes	no	yes	no	no	no
59	s4	Protecting the power of interventions thr	Vicki S	Intervention Content: What does the interventio	Provide clear operational descriptions	na	list	Table 1	no	yes	no	yes	no	no	no
60	s4	Protecting the power of interventions thr	Vicki S	Intervention Delivery: How was the intervention	Describe the delivery mode• Describe the physical and contextual setting	na	list	Table 1	no	yes	no	yes	no	no	no
61	s5	Improving the reporting quality of nonran	Des Jarlais	Title and abstract : Information on how units wer	na	na	list	Table 1	yes	yes	no	no	no	no	no
62	s5	Improving the reporting quality of nonran	Des Jarlais	Title and abstract : Structured abstract recomme	na	na	list	Table 1	yes	yes	no	no	no	no	no
63	s5	Improving the reporting quality of nonran	Des Jarlais	Title and abstract : Information on target populat	na	na	list	Table 1	yes	yes	no	no	no	no	no
64	s5	Improving the reporting quality of nonran	Des Jarlais	Background : Scientific background and explanati	na	na	list	Table 1	no	no	yes	no	no	no	no
65	s5	Improving the reporting quality of nonran	Des Jarlais	Background : Theories used in designing behavior	na	na	list	Table 1	no	no	yes	no	no	no	no
66	s5	Improving the reporting quality of nonran	Des Jarlais	Participants : Eligibility criteria for participants, in	na	na	list	Table 1	no	no	no	yes	no	no	no
67	s5	Improving the reporting quality of nonran	Des Jarlais	Participants : Method of recruitment (e.g., referr	na	na	list	Table 1	no	no	no	yes	no	no	no

68	s5	Improving the reporting quality of nonran	Des Jarlais	Participants : Recruitment setting	na	na	list	Table 1	no	no	no	yes	no	no	no
69	s5	Improving the reporting quality of nonran	Des Jarlais	Participants : Settings and locations where the da	na	na	list	Table 1	no	no	no	yes	no	no	no
70	s5	Improving the reporting quality of nonran	Des Jarlais	Interventions : Details of the interventions intenc	na	na	list	Table 1	no	no	no	yes	no	no	no
71	s5	Improving the reporting quality of nonran	Des Jarlais	Interventions : Details of the interventions intenc	na	na	list	Table 1	no	no	no	yes	no	no	no
72	s5	Improving the reporting quality of nonran	Des Jarlais	Interventions : Details of the interventions intenc	na	na	list	Table 1	no	no	no	yes	no	no	no
73	s5	Improving the reporting quality of nonran	Des Jarlais	Interventions : Details of the interventions intenc	na	na	list	Table 1	no	no	no	yes	no	no	no
74	s5	Improving the reporting quality of nonran	Des Jarlais	Interventions : Details of the interventions intenc	na	na	list	Table 1	no	no	no	yes	no	no	no
75	s5	Improving the reporting quality of nonran	Des Jarlais	Interventions : Details of the interventions intenc	na	na	list	Table 1	no	no	no	yes	no	no	no
76	s5	Improving the reporting quality of nonran	Des Jarlais	Interventions : Details of the interventions intenc	na	na	list	Table 1	no	no	no	yes	no	no	no
77	s5	Improving the reporting quality of nonran	Des Jarlais	Interventions : Details of the interventions intenc	na	na	list	Table 1	no	no	no	yes	no	no	no
78	s5	Improving the reporting quality of nonran	Des Jarlais	Objectives : Specific objectives and hypotheses	na	na	list	Table 1	no	no	no	yes	no	no	no
79	s5	Improving the reporting quality of nonran	Des Jarlais	Outcomes : Clearly defined primary and secondar	na	na	list	Table 1	no	no	no	yes	no	no	no
80	s5	Improving the reporting quality of nonran	Des Jarlais	Outcomes : Methods used to collect data and an	na	na	list	Table 1	no	no	no	yes	no	no	no
81	s5	Improving the reporting quality of nonran	Des Jarlais	Outcomes : Information on validated instruments	na	na	list	Table 1	no	no	no	yes	no	no	no
82	s5	Improving the reporting quality of nonran	Des Jarlais	Sample size : How sample size was determined ar	na	na	list	Table 1	no	no	no	yes	no	no	no
83	s5	Improving the reporting quality of nonran	Des Jarlais	Assignment method : Unit of assignment (the uni	na	na	list	Table 1	no	no	no	yes	no	no	no
84	s5	Improving the reporting quality of nonran	Des Jarlais	Assignment method : Method used to assign unit	na	na	list	Table 1	no	no	no	yes	no	no	no
85	s5	Improving the reporting quality of nonran	Des Jarlais	Assignment method : Inclusion of aspects employ	na	na	list	Table 1	no	no	no	yes	no	no	no
86	s5	Improving the reporting quality of nonran	Des Jarlais	Blinding (masking) : Whether or not participants,	na	na	list	Table 1	no	no	no	yes	no	no	no
87	s5	Improving the reporting quality of nonran	Des Jarlais	Unit of analysis : Description of the smallest unit	na	na	list	Table 1	no	no	no	yes	no	no	no
88	s5	Improving the reporting quality of nonran	Des Jarlais	Unit of analysis : If the unit of analysis differs fron	na	na	list	Table 1	no	no	no	yes	no	no	no
89	s5	Improving the reporting quality of nonran	Des Jarlais	Statistical methods : Statistical methods used to c	na	na	list	Table 1	no	no	no	yes	no	no	no
90	s5	Improving the reporting quality of nonran	Des Jarlais	Statistical methods : Statistical methods used for	na	na	list	Table 1	no	no	no	yes	no	no	no
91	s5	Improving the reporting quality of nonran	Des Jarlais	Statistical methods : Methods for imputing missir	na	na	list	Table 1	no	no	no	yes	no	no	no
92	s5	Improving the reporting quality of nonran	Des Jarlais	Statistical methods : Statistical software or progra	na	na	list	Table 1	no	no	no	yes	yes	no	no
93	s5	Improving the reporting quality of nonran	Des Jarlais	Participant flow : Flow of participants through ea	na	na	list	Table 1	no	no	no	no	yes	no	no
94	s5	Improving the reporting quality of nonran	Des Jarlais	Participant flow : Enrollment: the numbers of par	na	na	list	Table 1	no	no	no	no	yes	no	no
95	s5	Improving the reporting quality of nonran	Des Jarlais	Participant flow : Assignment: the numbers of pa	na	na	list	Table 1	no	no	no	no	yes	no	no
96	s5	Improving the reporting quality of nonran	Des Jarlais	Participant flow : Allocation and intervention exp	na	na	list	Table 1	no	no	no	no	yes	no	no
97	s5	Improving the reporting quality of nonran	Des Jarlais	Participant flow : Follow-up: the number of partic	na	na	list	Table 1	no	no	no	no	yes	no	no
98	s5	Improving the reporting quality of nonran	Des Jarlais	Participant flow : Analysis: the number of particip	na	na	list	Table 1	no	no	no	no	yes	no	no
99	s5	Improving the reporting quality of nonran	Des Jarlais	Participant flow : Description of protocol deviatio	na	na	list	Table 1	no	no	no	no	yes	no	no
100	s5	Improving the reporting quality of nonran	Des Jarlais	Recruitment : Dates defining the periods of recru	na	na	list	Table 1	no	no	no	no	yes	no	no
101	s5	Improving the reporting quality of nonran	Des Jarlais	Baseline data : Baseline demographic and clinical	na	na	list	Table 1	no	no	no	no	yes	no	no
102	s5	Improving the reporting quality of nonran	Des Jarlais	Baseline data : Baseline characteristics for each s	na	na	list	Table 1	no	no	no	no	yes	no	no
103	s5	Improving the reporting quality of nonran	Des Jarlais	Baseline data : Baseline comparisons of those los	na	na	list	Table 1	no	no	no	no	yes	no	no
104	s5	Improving the reporting quality of nonran	Des Jarlais	Baseline data : Comparison between study popul	na	na	list	Table 1	no	no	no	no	yes	no	no
105	s5	Improving the reporting quality of nonran	Des Jarlais	Baseline equivalence : Data on study group equiv	na	na	list	Table 1	no	no	no	no	yes	no	no
106	s5	Improving the reporting quality of nonran	Des Jarlais	Numbers analyzed : Number of participants (denc	na	na	list	Table 1	no	no	no	no	yes	no	no
107	s5	Improving the reporting quality of nonran	Des Jarlais	Numbers analyzed : Indication of whether the an	na	na	list	Table 1	no	no	no	no	yes	no	no
108	s5	Improving the reporting quality of nonran	Des Jarlais	Outcomes and estimation : For each primary and	na	na	list	Table 1	no	no	no	no	yes	no	no
109	s5	Improving the reporting quality of nonran	Des Jarlais	Outcomes and estimation : Inclusion of null and r	na	na	list	Table 1	no	no	no	no	yes	no	no
110	s5	Improving the reporting quality of nonran	Des Jarlais	Outcomes and estimation : Inclusion of results frc	na	na	list	Table 1	no	no	no	no	yes	no	no
111	s5	Improving the reporting quality of nonran	Des Jarlais	Ancillary analyses : Summary of other analyses pe	na	na	list	Table 1	no	no	no	no	yes	no	no
112	s5	Improving the reporting quality of nonran	Des Jarlais	Adverse events : Summary of all important adver	na	na	list	Table 1	no	no	no	no	no	yes	no
113	s5	Improving the reporting quality of nonran	Des Jarlais	Discussion Interpretation : Interpretation of the r	na	na	list	Table 1	no	no	no	no	no	yes	no
114	s5	Improving the reporting quality of nonran	Des Jarlais	Discussion Interpretation : Discussion of results ta	na	na	list	Table 1	no	no	no	no	no	yes	no
115	s5	Improving the reporting quality of nonran	Des Jarlais	Discussion Interpretation : Discussion of the succ	na	na	list	Table 1	no	no	no	no	no	yes	no
116	s5	Improving the reporting quality of nonran	Des Jarlais	Discussion Interpretation : Discussion of research	na	na	list	Table 1	no	no	no	no	no	yes	no
117	s5	Improving the reporting quality of nonran	Des Jarlais	Generalizability : Generalizability (external validit	na	na	list	Table 1	no	no	no	no	no	yes	no
118	s5	Improving the reporting quality of nonran	Des Jarlais	Overall evidence : General interpretation of the r	na	na	list	Table 1	no	no	no	no	no	yes	no
140	s7	Reviewing evidence on complex social int	Egan, M	Motivation	Does the study describe why the ma	na	list	Table 2	no	yes	yes	no	no	yes	no
141	s7	Reviewing evidence on complex social int	Egan, M	Theory of change	Was the intervention design influer	na	list	Table 2	no	yes	yes	no	no	yes	no
142	s7	Reviewing evidence on complex social int	Egan, M	Implementation context	Does the study provide any useful c	na	list	Table 2	yes	yes	yes	yes	yes	yes	no
143	s7	Reviewing evidence on complex social int	Egan, M	Experience	Does the study establish whether th	na	list	Table 2	no	yes	yes	yes	yes	yes	no
144	s7	Reviewing evidence on complex social int	Egan, M	Planning consultations	Is there a report of consultation/col	na	list	Table 2	no	no	no	yes	no	yes	no
145	s7	Reviewing evidence on complex social int	Egan, M	Delivery collaborations	Is there a report of consultation/col	na	list	Table 2	no	no	no	yes	no	yes	no
146	s7	Reviewing evidence on complex social int	Egan, M	Manager support	Were on-site managers/supervisors	na	list	Table 2	no	no	no	yes	no	yes	no
147	s7	Reviewing evidence on complex social int	Egan, M	Employee support	Were employees supportive of the	na	list	Table 2	no	no	no	yes	no	yes	no
148	s7	Reviewing evidence on complex social int	Egan, M	Resources	Does the study give information abc	na	list	Table 2	no	no	no	yes	no	yes	no
149	s7	Reviewing evidence on complex social int	Egan, M	Differential effects and population characteristics	Does the study provide information	na	list	Table 2	no	yes	no	no	yes	yes	no
150	S8	Reporting guidelines for implementation a	Hales, Simon	Title and abstract : Identify as implementation or	na	na	list	Table 2	yes	yes	no	no	no	no	no
151	S8	Reporting guidelines for implementation a	Hales, Simon	Background : "Explain the scientific background r	na	na	list	Table 2	no	no	yes	no	no	no	no
152	S8	Reporting guidelines for implementation a	Hales, Simon	Background : Describe the policy or programme c	na	na	list	Table 2	no	no	yes	no	no	no	no
153	S8	Reporting guidelines for implementation a	Hales, Simon	Background : What is it about implementation in	na	na	list	Table 2	no	no	yes	no	no	no	no
154	S8	Reporting guidelines for implementation a	Hales, Simon	Problem : "Briefly describe the nature and severit	na	na	list	Table 2	no	no	yes	no	no	no	no
155	S8	Reporting guidelines for implementation a	Hales, Simon	Problem : Specify who (champions/supporters) w	na	na	list	Table 2	no	no	yes	no	no	no	no
156	S8	Reporting guidelines for implementation a	Hales, Simon	Implementation strategy : Describe mechanisms	na	na	list	Table 2	no	no	yes	no	no	no	no
157	S8	Reporting guidelines for implementation a	Hales, Simon	Intervention : What evidence-based intervention	na	na	list	Table 2	no	no	yes	no	no	no	no

158	S8	Reporting guidelines for implementation a	Hales, Simon	Intended outcomes : Describe the specific aim of	na	na	list	Table 2	no	no	yes	no	no	no	no
159	S8	Reporting guidelines for implementation a	Hales, Simon	Study design : Identify the study design (for exam	na	na	list	Table 2	no	no	no	yes	no	no	no
160	S8	Reporting guidelines for implementation a	Hales, Simon	Setting : Exact details of study locations, baseline	na	na	list	Table 2	no	no	no	yes	no	no	no
161	S8	Reporting guidelines for implementation a	Hales, Simon	Implementation : "Give a description of the imple	na	na	list	Table 2	no	no	no	yes	no	no	no
162	S8	Reporting guidelines for implementation a	Hales, Simon	Implementation : Describe the intervention, (if re	na	na	list	Table 2	no	no	no	yes	no	no	no
163	S8	Reporting guidelines for implementation a	Hales, Simon	Implementation : Explain methods used to assure	na	na	list	Table 2	no	no	no	yes	no	no	no
164	S8	Reporting guidelines for implementation a	Hales, Simon	Participants : "For qualitative studies: what was t	na	na	list	Table 2	no	no	no	yes	no	no	no
165	S8	Reporting guidelines for implementation a	Hales, Simon	Participants : Indicate how size of target populati	na	na	list	Table 2	no	no	no	yes	no	no	no
166	S8	Reporting guidelines for implementation a	Hales, Simon	Participants : Cohort study – Give the eligibility cr	na	na	list	Table 2	no	no	no	yes	no	no	no
167	S8	Reporting guidelines for implementation a	Hales, Simon	Participants : Case-control study – Give the eligib	na	na	list	Table 2	no	no	no	yes	no	no	no
168	S8	Reporting guidelines for implementation a	Hales, Simon	Participants : Cross-sectional study – Give the elig	na	na	list	Table 2	no	no	no	yes	no	no	no
169	S8	Reporting guidelines for implementation a	Hales, Simon	Participants : For matched studies, give matching	na	na	list	Table 2	no	no	no	yes	no	no	no
170	S8	Reporting guidelines for implementation a	Hales, Simon	Participants : For randomized studies, how was ra	na	na	list	Table 2	no	no	no	yes	no	no	no
171	S8	Reporting guidelines for implementation a	Hales, Simon	Variables : Clearly define all outcomes, exposures	na	na	list	Table 2	no	no	no	yes	no	no	no
172	S8	Reporting guidelines for implementation a	Hales, Simon	Data sources/measurement : "For each variable c	na	na	list	Table 2	no	no	no	yes	no	no	no
173	S8	Reporting guidelines for implementation a	Hales, Simon	Data sources/measurement : Methods for proces	na	na	list	Table 2	no	no	no	yes	no	no	no
174	S8	Reporting guidelines for implementation a	Hales, Simon	Data sources/measurement : Explain how variabl	na	na	list	Table 2	no	no	no	yes	no	no	no
175	S8	Reporting guidelines for implementation a	Hales, Simon	Analyses : "Which analyses were pre-specified, ar	na	na	list	Table 2	no	no	no	yes	no	no	no
176	S8	Reporting guidelines for implementation a	Hales, Simon	Analyses : For qualitative analyses: process by wh	na	na	list	Table 2	no	no	no	yes	no	no	no
177	S8	Reporting guidelines for implementation a	Hales, Simon	Analyses : For quantitative analyses: describe stat	na	na	list	Table 2	no	no	no	yes	no	no	no
178	S8	Reporting guidelines for implementation a	Hales, Simon	Analyses : Where both qualitative and quantitativ	na	na	list	Table 2	no	no	no	yes	no	no	no
179	S8	Reporting guidelines for implementation a	Hales, Simon	Analyses : Describe any methods used to examine	na	na	list	Table 2	no	no	no	yes	no	no	no
180	S8	Reporting guidelines for implementation a	Hales, Simon	Analyses : Explain how missing data were address	na	na	list	Table 2	no	no	no	yes	no	no	no
181	S8	Reporting guidelines for implementation a	Hales, Simon	Analyses : Cohort study: explain how loss to follo	na	na	list	Table 2	no	no	no	yes	no	no	no
182	S8	Reporting guidelines for implementation a	Hales, Simon	Analyses : Case-control study: describe matching	na	na	list	Table 2	no	no	no	yes	no	no	no
183	S8	Reporting guidelines for implementation a	Hales, Simon	Ethical considerations : "Including consent proce	na	na	list	Table 2	no	no	no	yes	no	no	no
184	S8	Reporting guidelines for implementation a	Hales, Simon	Ethical considerations : How was the balance bet	na	na	list	Table 2	no	no	no	yes	no	no	no
185	S8	Reporting guidelines for implementation a	Hales, Simon	Descriptive data : "Report numbers of individuals	na	na	list	Table 2	no	no	no	no	yes	no	no
186	S8	Reporting guidelines for implementation a	Hales, Simon	Descriptive data : Cross tabulate the number of p	na	na	list	Table 2	no	no	no	no	yes	no	no
187	S8	Reporting guidelines for implementation a	Hales, Simon	Outcomes : "Explain the actual course of the inte	na	na	list	Table 2	no	no	no	no	yes	no	no
188	S8	Reporting guidelines for implementation a	Hales, Simon	Outcomes : Document the degree of success in ir	na	na	list	Table 2	no	no	no	no	yes	no	no
189	S8	Reporting guidelines for implementation a	Hales, Simon	Outcomes : Document the degree of success in ir	na	na	list	Table 2	no	no	no	no	yes	no	no
190	S8	Reporting guidelines for implementation a	Hales, Simon	Outcomes : Document the degree of success in ir	na	na	list	Table 2	no	no	no	no	yes	no	no
191	S8	Reporting guidelines for implementation a	Hales, Simon	Outcome data : Report numbers of outcome eve	na	na	list	Table 2	no	no	no	no	yes	no	no
192	S8	Reporting guidelines for implementation a	Hales, Simon	Main results : "Main findings (e.g. interpretations	na	na	list	Table 2	no	no	no	no	yes	no	no
193	S8	Reporting guidelines for implementation a	Hales, Simon	Main results : Provide unadjusted estimates of in	na	na	list	Table 2	no	no	no	no	yes	no	no
194	S8	Reporting guidelines for implementation a	Hales, Simon	Main results : Consider translating estimates of re	na	na	list	Table 2	no	no	no	no	yes	no	no
195	S8	Reporting guidelines for implementation a	Hales, Simon	Main results : Synthesis of quantitative and qualit	na	na	list	Table 2	no	no	no	no	yes	no	no
196	S8	Reporting guidelines for implementation a	Hales, Simon	Other analyses : Report other analyses done – e.g	na	na	list	Table 2	no	no	no	no	yes	no	no
197	S8	Reporting guidelines for implementation a	Hales, Simon	Key results : Summarize key results with referenc	na	na	list	Table 2	no	no	no	no	no	yes	no
198	S8	Reporting guidelines for implementation a	Hales, Simon	Limitations : "Discuss limitations of the study, tak	na	na	list	Table 2	no	no	no	no	no	yes	no
199	S8	Reporting guidelines for implementation a	Hales, Simon	Limitations : Discuss both direction and magnituc	na	na	list	Table 2	no	no	no	no	no	yes	no
200	S8	Reporting guidelines for implementation a	Hales, Simon	Interpretation : "Interpret the results considering	na	na	list	Table 2	no	no	no	no	no	yes	no
201	S8	Reporting guidelines for implementation a	Hales, Simon	Interpretation : Compare and contrast study resu	na	na	list	Table 2	no	no	no	no	no	yes	no
202	S8	Reporting guidelines for implementation a	Hales, Simon	Interpretation : Suggest steps that might be mod	na	na	list	Table 2	no	no	no	no	no	yes	no
203	S8	Reporting guidelines for implementation a	Hales, Simon	Interpretation : Review issues of opportunity cost	na	na	list	Table 2	no	no	no	no	no	yes	no
204	S8	Reporting guidelines for implementation a	Hales, Simon	Contextual factors : Success factors, barriers and	na	na	list	Table 2	no	no	no	no	no	yes	no
205	S8	Reporting guidelines for implementation a	Hales, Simon	Generalizability : "Discuss the generalizability (ex	na	na	list	Table 2	no	no	no	no	no	yes	no
206	S8	Reporting guidelines for implementation a	Hales, Simon	Generalizability : Explore factors that could affect	na	na	list	Table 2	no	no	no	no	no	yes	no
207	S8	Reporting guidelines for implementation a	Hales, Simon	Generalizability : Applicability to other settings; P	na	na	list	Table 2	no	no	no	no	no	yes	no
208	S8	Reporting guidelines for implementation a	Hales, Simon	Conclusion : "Consider overall practical usefulnes	na	na	list	Table 2	no	no	no	no	no	yes	no
209	S8	Reporting guidelines for implementation a	Hales, Simon	Conclusion : Suggest implications for the impleme	na	na	list	Table 2	no	no	no	no	no	yes	no
210	S8	Reporting guidelines for implementation a	Hales, Simon	Conclusion : Suggest implications for further stud	na	na	list	Table 2	no	no	no	no	no	yes	no
211	S8	Reporting guidelines for implementation a	Hales, Simon	Other information : "Indicate if the study is regist	na	na	list	Table 2	no	no	no	no	no	no	yes
212	S8	Reporting guidelines for implementation a	Hales, Simon	Other information : Give the source of funding ar	na	na	list	Table 2	no	no	no	no	no	no	yes
213	S8	Reporting guidelines for implementation a	Hales, Simon	Other information : State the role of individuals ir	na	na	list	Table 2	no	no	no	no	no	no	yes
214	S9	Reporting standards for studies of tailorec	Harrington, Nancy G	Title, abstract, keywords : Include some variation	na	na	list	Table 1	yes	yes	no	no	no	no	yes
215	S9	Reporting standards for studies of tailorec	Harrington, Nancy G	Variables/constructs : Specify variables/construct	na	na	list	Table 1	no	no	no	yes	no	no	no
216	S9	Reporting standards for studies of tailorec	Harrington, Nancy G	Theoretical foundation : Describe how theory gui	na	na	list	Table 1	no	no	no	yes	no	no	no
217	S9	Reporting standards for studies of tailorec	Harrington, Nancy G	Tailored messages : Describe the type of the tailo	na	na	list	Table 1	no	no	no	yes	no	no	no
218	S9	Reporting standards for studies of tailorec	Harrington, Nancy G	Tailoring system : Describe the tailoring system a	na	na	list	Table 1	no	no	no	yes	no	no	no
219	S9	Reporting standards for studies of tailorec	Harrington, Nancy G	Intervention channel, format, dosage and contex	na	na	list	Table 1	no	no	no	yes	no	no	no
220	S9	Reporting standards for studies of tailorec	Harrington, Nancy G	Intervention implementation and assessment : Describe how frequently participants received intervention content and when they received		na	list	Table 1	no	no	no	yes	no	no	no
221	S10	Better reporting of interventions: templat	Hoffmann, Tammy C	Brief name: Provide the name or a phrase that de	Precision in the name, or brief description, of an	na	list	Table 1	no	no	no	yes	no	no	no
222	S10	Better reporting of interventions: templat	Hoffmann, Tammy C	Why: Describe any rationale, theory, or goal of th	Inclusion of the rationale, theory, or goals that	na	list	Table 1	no	no	no	yes	no	no	no
223	S10	Better reporting of interventions: templat	Hoffmann, Tammy C	What (materials): Describe any physical or inform	A full description of an intervention should	na	list	Table 1	no	no	no	yes	no	no	no
224	S10	Better reporting of interventions: templat	Hoffmann, Tammy C	What (procedures): Describe each of the procedu	Describe what processes, activities, or procedures	na	list	Table 1	no	no	no	yes	no	no	no
225	S10	Better reporting of interventions: templat	Hoffmann, Tammy C	Who provided: For each category of intervention	The term “intervention provider” refers to who	na	list	Table 1	no	no	no	yes	no	no	no
226	S10	Better reporting of interventions: templat	Hoffmann, Tammy C	How: Describe the modes of delivery (such as fac	Specify whether the intervention was provided	na	list	Table 1	no	no	no	yes	no	no	no

227	S10	Better reporting of interventions: templat	Hoffmann, Tammy C	Where: Describe the type(s) of location(s) where	In some studies the intervention can be delivered	na	list	Table 1	no	no	no	yes	no	no	no
228	S10	Better reporting of interventions: templat	Hoffmann, Tammy C	When and how much: Describe the number of tir	The type of information needed about the "when	na	list	Table 1	no	no	no	yes	no	no	no
229	S10	Better reporting of interventions: templat	Hoffmann, Tammy C	Tailoring: If the intervention wasplanned to be personalised, titrated or	In tailored interventions, not all participants	na	list	Table 1	no	no	no	yes	no	no	no
230	S10	Better reporting of interventions: templat	Hoffmann, Tammy C	Modifications: If the intervention was modified d	This item refers to modifications that occur at	na	list	Table 1	no	no	no	yes	no	no	no
231	S10	Better reporting of interventions: templat	Hoffmann, Tammy C	How well (planned): If intervention adherence or	Fidelity refers to the degree to whic	na	list	Table 1	no	no	no	yes	no	no	no
232	S10	Better reporting of interventions: templat	Hoffmann, Tammy C	How well (actual): If intervention adherence or fi	For various reasons, an intervention, or parts of	na	list	Table 1	no	no	no	yes	no	no	no
233	S11	Reporting on innovative public health inte	Huston, Patricia	Has the author clearly identified the issue that pr	na	na	list	Table 1	no	no	yes	no	no	no	no
234	S11	Reporting on innovative public health inte	Huston, Patricia	Is this issue pertinent to public health?	na	na	list	Table 1	no	no	yes	no	no	no	no
235	S11	Reporting on innovative public health inte	Huston, Patricia	Has the appropriate literature been	na	na	list	Table 1	no	no	yes	no	no	no	no
236	S11	Reporting on innovative public health inte	Huston, Patricia	Are the objectives of the intervention cle	na	na	list	Table 1	no	no	yes	no	no	no	no
237	S11	Reporting on innovative public health inte	Huston, Patricia	Target Population : Is the target population clearl	na	na	list	Table 1	no	no	no	yes	no	no	no
238	S11	Reporting on innovative public health inte	Huston, Patricia	Target Population : Is the method used to reach t	na	na	list	Table 1	no	no	no	yes	no	no	no
239	S11	Reporting on innovative public health inte	Huston, Patricia	setting : Is the context of the study clear?	na	na	list	Table 1	no	no	no	yes	no	no	no
240	S11	Reporting on innovative public health inte	Huston, Patricia	Public Health Intervention : Is the intervention cl	na	na	list	Table 1	no	no	no	yes	no	no	no
241	S11	Reporting on innovative public health inte	Huston, Patricia	Public Health Intervention : Is the preliminary eva	na	na	list	Table 1	no	no	no	yes	no	no	no
242	S11	Reporting on innovative public health inte	Huston, Patricia	Public Health Intervention : Were meaningful out	na	na	list	Table 1	no	no	no	yes	no	no	no
243	S11	Reporting on innovative public health inte	Huston, Patricia	Is a descriptive profile of participants given?	na	na	list	Table 1	no	no	no	no	yes	no	no
244	S11	Reporting on innovative public health inte	Huston, Patricia	Is the level of participation noted	na	na	list	Table 1	no	no	no	no	yes	no	no
245	S11	Reporting on innovative public health inte	Huston, Patricia	Are the successes and challenges of delivering th	na	na	list	Table 1	no	no	no	no	yes	no	no
246	S11	Reporting on innovative public health inte	Huston, Patricia	Are the outcomes appropriately described	na	na	list	Table 1	no	no	no	no	yes	no	no
247	S11	Reporting on innovative public health inte	Huston, Patricia	Are the major findings highlighted	na	na	list	Table 1	no	no	no	no	yes	no	no
248	S11	Reporting on innovative public health inte	Huston, Patricia	Are findings discussed with reference to existing	na	na	list	Table 1	no	no	no	no	no	yes	no
249	S11	Reporting on innovative public health inte	Huston, Patricia	Have strengths and weaknesses been considered	na	na	list	Table 1	no	no	no	no	no	yes	no
250	S11	Reporting on innovative public health inte	Huston, Patricia	Are the lessons learned from the intervention dis	na	na	list	Table 1	no	no	no	no	no	yes	no
251	S11	Reporting on innovative public health inte	Huston, Patricia	Are the implications for future initiatives explore	na	na	list	Table 1	no	no	no	no	no	yes	no
252	S12	Programme Reporting Standards (PRS) for	Kågesten, Anna E	Programme Overview : Rationale and objectives :	na	na	list	Table 5	no	yes	yes	no	no	no	no
253	S12	Programme Reporting Standards (PRS) for	Kågesten, Anna E	Programme Overview : Rationale and objectives :	na	na	list	Table 5	no	yes	yes	no	no	no	no
254	S12	Programme Reporting Standards (PRS) for	Kågesten, Anna E	Programme Overview : Rationale and objectives :	na	na	list	Table 5	no	yes	yes	yes	no	no	no
255	S12	Programme Reporting Standards (PRS) for	Kågesten, Anna E	Programme Overview : Start and end date : "Plan	na	na	list	Table 5	no	yes	no	yes	no	no	no
256	S12	Programme Reporting Standards (PRS) for	Kågesten, Anna E	Programme Overview : Start and end date : b. De	na	na	list	Table 5	no	yes	no	yes	no	no	no
257	S12	Programme Reporting Standards (PRS) for	Kågesten, Anna E	Programme Overview : Setting and Context : "Wh	na	na	list	Table 5	no	yes	no	yes	no	no	no
258	S12	Programme Reporting Standards (PRS) for	Kågesten, Anna E	Programme Overview : Setting and Context : b. O	na	na	list	Table 5	no	yes	no	yes	no	no	no
259	S12	Programme Reporting Standards (PRS) for	Kågesten, Anna E	Programme Overview : Stakeholders : "a. Prograr	na	na	list	Table 5	no	yes	no	yes	no	no	no
260	S12	Programme Reporting Standards (PRS) for	Kågesten, Anna E	Programme Overview : Stakeholders : b. Impleme	na	na	list	Table 5	no	yes	no	yes	no	no	no
261	S12	Programme Reporting Standards (PRS) for	Kågesten, Anna E	Programme Overview : Stakeholders : c. Partners	na	na	list	Table 5	no	yes	no	yes	no	no	no
262	S12	Programme Reporting Standards (PRS) for	Kågesten, Anna E	Programme Overview : Stakeholders : d. How the	na	na	list	Table 5	no	yes	no	yes	no	no	no
263	S12	Programme Reporting Standards (PRS) for	Kågesten, Anna E	Programme Overview : Funding source(s) : Name	na	na	list	Table 5	no	yes	no	no	no	no	yes
264	S12	Programme Reporting Standards (PRS) for	Kågesten, Anna E	Programme Overview : Theory of change and/or	na	na	list	Table 5	no	yes	no	yes	no	no	no
265	S12	Programme Reporting Standards (PRS) for	Kågesten, Anna E	Programme Overview : Human rights perspective	na	na	list	Table 5	no	yes	no	yes	no	no	no
266	S12	Programme Reporting Standards (PRS) for	Kågesten, Anna E	Programme Overview : Human rights perspective	na	na	list	Table 5	no	no	no	yes	no	no	no
267	S12	Programme Reporting Standards (PRS) for	Kågesten, Anna E	Programme Components and Implementation : P	na	na	list	Table 5	no	no	no	yes	no	no	no
268	S12	Programme Reporting Standards (PRS) for	Kågesten, Anna E	Programme Components and Implementation : P	na	na	list	Table 5	no	no	no	yes	no	no	no
269	S12	Programme Reporting Standards (PRS) for	Kågesten, Anna E	Programme Components and Implementation : C	na	na	list	Table 5	no	no	no	yes	no	no	no
270	S12	Programme Reporting Standards (PRS) for	Kågesten, Anna E	Programme Components and Implementation : C	na	na	list	Table 5	no	no	no	yes	no	no	no
271	S12	Programme Reporting Standards (PRS) for	Kågesten, Anna E	Programme Components and Implementation : C	na	na	list	Table 5	no	no	no	yes	no	no	no
272	S12	Programme Reporting Standards (PRS) for	Kågesten, Anna E	Programme Components and Implementation : C	na	na	list	Table 5	no	no	no	yes	no	no	no
273	S12	Programme Reporting Standards (PRS) for	Kågesten, Anna E	Programme Components and Implementation : C	na	na	list	Table 5	no	no	no	yes	no	no	no
274	S12	Programme Reporting Standards (PRS) for	Kågesten, Anna E	Programme Components and Implementation : C	na	na	list	Table 5	no	no	no	yes	no	no	no
275	S12	Programme Reporting Standards (PRS) for	Kågesten, Anna E	Programme Components and Implementation : C	na	na	list	Table 5	no	no	no	yes	no	no	no
276	S12	Programme Reporting Standards (PRS) for	Kågesten, Anna E	Programme Components and Implementation : C	na	na	list	Table 5	no	no	no	yes	no	no	no
277	S12	Programme Reporting Standards (PRS) for	Kågesten, Anna E	Monitoring of Implementation : Monitoring mech	na	na	list	Table 5	no	no	no	yes	no	no	no
278	S12	Programme Reporting Standards (PRS) for	Kågesten, Anna E	Monitoring of Implementation : Coverage/Reach	na	na	list	Table 5	no	no	no	yes	no	no	no
279	S12	Programme Reporting Standards (PRS) for	Kågesten, Anna E	Monitoring of Implementation : Coverage/Reach	na	na	list	Table 5	no	no	no	yes	no	no	no
280	S12	Programme Reporting Standards (PRS) for	Kågesten, Anna E	Monitoring of Implementation : Coverage/Reach	na	na	list	Table 5	no	no	no	yes	no	no	no
281	S12	Programme Reporting Standards (PRS) for	Kågesten, Anna E	Monitoring of Implementation : Adaptations : "W	na	na	list	Table 5	no	no	no	yes	no	no	no
282	S12	Programme Reporting Standards (PRS) for	Kågesten, Anna E	Monitoring of Implementation : Adaptations : b. (	na	na	list	Table 5	no	no	no	yes	no	no	no
283	S12	Programme Reporting Standards (PRS) for	Kågesten, Anna E	Monitoring of Implementation : Acceptability : "A	na	na	list	Table 5	no	no	no	yes	no	no	no
284	S12	Programme Reporting Standards (PRS) for	Kågesten, Anna E	Monitoring of Implementation : Feasibility : "Asse	na	na	list	Table 5	no	no	no	yes	no	no	no
285	S12	Programme Reporting Standards (PRS) for	Kågesten, Anna E	Monitoring of Implementation : Factors affecting	na	na	list	Table 5	no	no	no	yes	no	no	no
286	S12	Programme Reporting Standards (PRS) for	Kågesten, Anna E	Evaluation and Results : Evaluation : "a. Type of e	na	na	list	Table 5	no	no	no	yes	no	no	no
287	S12	Programme Reporting Standards (PRS) for	Kågesten, Anna E	Evaluation and Results : Evaluation : b. Evaluatior	na	na	list	Table 5	no	no	no	yes	no	no	no
288	S12	Programme Reporting Standards (PRS) for	Kågesten, Anna E	Evaluation and Results : Results : "Description of	na	na	list	Table 5	no	no	no	no	yes	no	no
289	S12	Programme Reporting Standards (PRS) for	Kågesten, Anna E	Evaluation and Results : Results : b. Whether the	na	na	list	Table 5	no	no	no	no	yes	no	no
290	S12	Programme Reporting Standards (PRS) for	Kågesten, Anna E	Evaluation and Results : Results : c. Whether the	na	na	list	Table 5	no	no	no	no	yes	no	no
291	S12	Programme Reporting Standards (PRS) for	Kågesten, Anna E	Evaluation and Results : Costs : "Summary of the	na	na	list	Table 5	no	no	no	no	yes	no	no
292	S12	Programme Reporting Standards (PRS) for	Kågesten, Anna E	Evaluation and Results : Costs : b. If and how a co	na	na	list	Table 5	no	no	no	no	yes	no	no
293	S12	Programme Reporting Standards (PRS) for	Kågesten, Anna E	Synthesis : Lessons learnt : "Appraised weakness	na	na	list	Table 5	no	no	no	no	no	yes	no
294	S12	Programme Reporting Standards (PRS) for	Kågesten, Anna E	Synthesis : Sustainability : "Reflections on the sus	na	na	list	Table 5	no	no	no	no	no	yes	no
295	S12	Programme Reporting Standards (PRS) for	Kågesten, Anna E	Synthesis : Scalability : "Description of the scale-u	na	na	list	Table 5	no	no	no	no	no	yes	no

296	S12	Programme Reporting Standards (PRS) for	Kågesten, Anna E	Synthesis : Possibilities for implementation in oth	na	na	list	Table 5	no	no	no	no	no	yes	no
297	S12	Programme Reporting Standards (PRS) for	Kågesten, Anna E	Synthesis : Additional information (optional) : "Re	na	na	list	Table 5	no	no	no	no	no	yes	no
298	S12	Programme Reporting Standards (PRS) for	Kågesten, Anna E	Synthesis : Additional information (optional) : Any	na	na	list	Table 5	no	no	no	no	no	yes	no
299	S13	Reporting of context and implementation	Luoto Jill	Intervention characteristics: Intervention/Progra	na	na	list	Table 1	no	no	no	yes	no	no	no
300	S13	Reporting of context and implementation	Luoto Jill	Intervention characteristics: A description of why	na	na	list	Table 1	no	no	no	yes	no	no	no
301	S13	Reporting of context and implementation	Luoto Jill	Intervention characteristics: Rationale for the ain	na	na	list	Table 1	no	no	no	yes	no	no	no
302	S13	Reporting of context and implementation	Luoto Jill	Intervention Characteristics: Detailed description	na	na	list	Table 1	no	no	no	yes	no	no	no
303	S13	Reporting of context and implementation	Luoto Jill	Intervention Characteristics: Detailed description	na	na	list	Table 1	no	no	no	yes	no	no	no
304	S13	Reporting of context and implementation	Luoto Jill	Intervention Characteristics: Detailed description	na	na	list	Table 1	no	no	no	yes	no	no	no
305	S13	Reporting of context and implementation	Luoto Jill	Intervention Characteristics: Detailed description	na	na	list	Table 1	no	no	no	yes	no	no	no
306	S13	Reporting of context and implementation	Luoto Jill	Intervention Characteristics: Detailed description	na	na	list	Table 1	no	no	no	yes	no	no	no
307	S13	Reporting of context and implementation	Luoto Jill	Intervention Characteristics: Detailed description	na	na	list	Table 1	no	no	no	yes	no	no	no
308	S13	Reporting of context and implementation	Luoto Jill	Intervention Characteristics: Detailed description	na	na	list	Table 1	no	no	no	yes	no	no	no
309	S13	Reporting of context and implementation	Luoto Jill	Intervention Characteristics: Detailed description	na	na	list	Table 1	no	no	no	yes	no	no	no
310	S13	Reporting of context and implementation	Luoto Jill	Intervention Characteristics: Costs of the interver	na	na	list	Table 1	no	no	no	yes	no	no	no
311	S13	Reporting of context and implementation	Luoto Jill	Outer Setting: External policies and incentives	na	na	list	Table 1	no	no	no	yes	no	no	no
312	S13	Reporting of context and implementation	Luoto Jill	Population needs	na	na	list	Table 1	no	no	no	yes	no	no	no
313	S13	Reporting of context and implementation	Luoto Jill	Process of implementation: Description of facilita	na	na	list	Table 1	no	no	no	yes	no	no	no
314	S13	Reporting of context and implementation	Luoto Jill	Description of materials: Description of all materi	na	na	list	Table 1	no	no	no	yes	no	no	no
315	S13	Reporting of context and implementation	Luoto Jill	Process of Implementation: Description of an ass	na	na	list	Table 1	no	no	no	yes	no	no	no
348	S15	A Framework for Enhancing the Value of F	Neta Gila	Goal	Improvementsin populationhealth,I	na	list	fig 1	no	no	Yes	no	no	no	no
349	S15	A Framework for Enhancing the Value of F	Neta Gila	Planning : Clinical, health system, orpublic health intervention: Evidence Base	Cross-Cutting Issues:Multilevel context (including history, policy climate, and incentives)	na	list	fig 1	no	no	no	Yes	no	no	no
350	S15	A Framework for Enhancing the Value of F	Neta Gila	Planning : Clinical, health system, orpublic health intervention: Program Logic	Cross-Cutting Issues:Multilevel context (including history, policy climate, and incentives)	na	list	fig 1	no	no	no	Yes	no	no	no
351	S15	A Framework for Enhancing the Value of F	Neta Gila	Planning : Clinical, health system, orpublic health intervention: Mechanisms of Change	Cross-Cutting Issues:Multilevel context (including history, policy climate, and incentives)	na	list	fig 1	no	no	no	Yes	no	no	no
352	S15	A Framework for Enhancing the Value of F	Neta Gila	Planning : Setting characteristics : Setting characteristics	Cross-Cutting Issues:Multilevel context (including history, policy climate, and incentives)	na	list	fig 1	no	no	no	Yes	no	no	no
353	S15	A Framework for Enhancing the Value of F	Neta Gila	Planning : Setting characteristics : organizational	Cross-Cutting Issues:Multilevel context (including history, policy climate, and incentives)	na	list	fig 1	no	no	no	Yes	no	no	no
354	S15	A Framework for Enhancing the Value of F	Neta Gila	Planning : Setting characteristics : climate and cu	Cross-Cutting Issues:Multilevel context (including history, policy climate, and incentives)	na	list	fig 1	no	no	no	Yes	no	no	no
355	S15	A Framework for Enhancing the Value of F	Neta Gila	Planning : Setting characteristics : capacityand rea	Cross-Cutting Issues:Multilevel context (including history, policy climate, and incentives)	na	list	fig 1	no	no	no	Yes	no	no	no
356	S15	A Framework for Enhancing the Value of F	Neta Gila	Planning : Implementation strategy : Evaluability	Cross-Cutting Issues:Multilevel context (including history, policy climate, and incentives)	na	list	fig 1	no	no	no	Yes	no	no	no
357	S15	A Framework for Enhancing the Value of F	Neta Gila	Planning : Implementation strategy : Scalability	Cross-Cutting Issues:Multilevel context (including history, policy climate, and incentives)	na	list	fig 1	no	no	no	Yes	no	no	no
358	S15	A Framework for Enhancing the Value of F	Neta Gila	Planning : Partnership	Cross-Cutting Issues:Multilevel context (including history, policy climate, and incentives)	na	list	fig 1	no	no	no	Yes	no	no	no
359	S15	A Framework for Enhancing the Value of F	Neta Gila	Planning : D&I study design	Cross-Cutting Issues:Multilevel context (including history, policy climate, and incentives)	na	list	fig 1	no	no	no	Yes	no	no	no
360	S15	A Framework for Enhancing the Value of F	Neta Gila	Delivery : Reach	Cross-Cutting Issues:Multilevel context (including history, policy climate, and incentives)	na	list	fig 1	no	no	no	Yes	no	no	no
361	S15	A Framework for Enhancing the Value of F	Neta Gila	Delivery : Adoptiona	Cross-Cutting Issues:Multilevel context (including history, policy climate, and incentives)	na	list	fig 1	no	no	no	Yes	no	no	no
362	S15	A Framework for Enhancing the Value of F	Neta Gila	Delivery : Evolution of intervention andimplementation strategy to	Cross-Cutting Issues:Multilevel context (including history, policy climate, and incentives)	na	list	fig 1	no	no	no	Yes	no	no	no
363	S15	A Framework for Enhancing the Value of F	Neta Gila	Delivery : Implementation : Fidelity,	Cross-Cutting Issues:Multilevel context (including history, policy climate, and incentives)	na	list	fig 1	no	no	no	Yes	no	no	no
364	S15	A Framework for Enhancing the Value of F	Neta Gila	Delivery : Implementation : dose,	Cross-Cutting Issues:Multilevel context (including history, policy climate, and incentives)	na	list	fig 1	no	no	no	Yes	no	no	no
365	S15	A Framework for Enhancing the Value of F	Neta Gila	Delivery : Implementation : and adaptation	Cross-Cutting Issues:Multilevel context (including history, policy climate, and incentives)	na	list	fig 1	no	no	no	Yes	no	no	no
366	S15	A Framework for Enhancing the Value of F	Neta Gila	Delivery : Implementation costs andresources exp	Cross-Cutting Issues:Multilevel context (including history, policy climate, and incentives)	na	list	fig 1	no	no	no	Yes	no	no	no
367	S15	A Framework for Enhancing the Value of F	Neta Gila	Evaluation/ResultsReporting : Effectiveness	Cross-Cutting Issues:Multilevel context (including history, policy climate, and incentives)	na	list	fig 1	no	no	no	Yes	Yes	no	no
368	S15	A Framework for Enhancing the Value of F	Neta Gila	Evaluation/ResultsReporting : Primary outcome	Cross-Cutting Issues:Multilevel context (including history, policy climate, and incentives)	na	list	fig 1	no	no	no	Yes	Yes	no	no
369	S15	A Framework for Enhancing the Value of F	Neta Gila	Evaluation/ResultsReporting : Broader consequences (e.g.,	Cross-Cutting Issues:Multilevel context (including history, policy climate, and incentives)	na	list	fig 1	no	no	no	Yes	Yes	no	no
370	S15	A Framework for Enhancing the Value of F	Neta Gila	Evaluation/ResultsReporting : External validity of findings,	Cross-Cutting Issues:Multilevel context (including history, policy climate, and incentives)	na	list	fig 1	no	no	no	Yes	Yes	no	no
371	S15	A Framework for Enhancing the Value of F	Neta Gila	Evaluation/ResultsReporting : Robustness	Cross-Cutting Issues:Multilevel context (including history, policy climate, and incentives)	na	list	fig 1	no	no	no	Yes	Yes	no	no
372	S15	A Framework for Enhancing the Value of F	Neta Gila	Evaluation/ResultsReporting : Pragmatic criteriaa	Cross-Cutting Issues:Multilevel context (including history, policy climate, and incentives)	na	list	fig 1	no	no	no	Yes	Yes	no	no
373	S15	A Framework for Enhancing the Value of F	Neta Gila	Long-Term Outcomes : Sustainability	Cross-Cutting Issues:Multilevel context (including history, policy climate, and incentives)	na	list	fig 1	no	no	no	Yes	Yes	no	no
374	S15	A Framework for Enhancing the Value of F	Neta Gila	Long-Term Outcomes : Evolvability	Cross-Cutting Issues:Multilevel context (including history, policy climate, and incentives)	na	list	fig 1	no	no	no	Yes	Yes	no	no
375	S15	A Framework for Enhancing the Value of F	Neta Gila	Long-Term Outcomes : Transportability	Cross-Cutting Issues:Multilevel context (including history, policy climate, and incentives)	na	list	fig 1	no	no	no	Yes	Yes	no	no
376	S15	A Framework for Enhancing the Value of F	Neta Gila	Long-TermOutcomes : Replication and uptake:Conditions under which	Cross-Cutting Issues:Multilevel context (including history, policy climate, and incentives)	na	list	fig 1	no	no	no	Yes	Yes	no	no
377	S15	A Framework for Enhancing the Value of F	Neta Gila	Long-TermOutcomes : Economic evaluationaeffectiveness, budget impact,	Cross-Cutting Issues:Multilevel context (including history, policy climate, and incentives)	na	list	fig 1	no	no	no	Yes	Yes	no	no
378	S16	Development of a framework for reportin	O'Donnell Siobhan	why: Why was the health service deliverymodel f	Goals of the model Goals related to patient outcome(s);	na	list	table 2	no	no	no	yes	no	no	no
379	S16	Development of a framework for reportin	O'Donnell Siobhan	Who: Who was involved?	Provider(s) Health care professional	na	list	table 2	no	no	no	yes	no	no	no
380	S16	Development of a framework for reportin	O'Donnell Siobhan	what: What were the roles of theindividuals invol	Role of provider(s)Role of user(s)	na	list	table 2	no	no	no	yes	no	no	no
381	S16	Development of a framework for reportin	O'Donnell Siobhan	When: When were the health services/interventi	Duration of disease since onset ofsy	na	list	table 2	no	no	no	yes	no	no	no
382	S16	Development of a framework for reportin	O'Donnell Siobhan	where: Where were the health services/intervent	SettingCountryLevel of care: commu	na	list	table 2	no	no	no	yes	no	no	no
383	S16	Development of a framework for reportin	O'Donnell Siobhan	How: How did patients access the service(s)/inter	Referral process	na	list	table 2	no	no	no	yes	no	no	no
384	S16	Development of a framework for reportin	O'Donnell Siobhan	How: How were the services/interventions imple	Method(s) in which the interventions	na	list	table 2	no	no	no	yes	no	no	no
385	S16	Development of a framework for reportin	O'Donnell Siobhan	How: How long was the intervention?	Duration of the intervention	na	list	table 2	no	no	no	yes	no	no	no
386	S16	Development of a framework for reportin	O'Donnell Siobhan	How: How did individuals involved communicate?	Mode(s) of communication (Among providers; between	na	list	table 2	no	no	no	yes	no	no	no
387	S16	Development of a framework for reportin	O'Donnell Siobhan	How: How was the health service delivery model	Resources needed to support orsus	na	list	table 2	no	no	no	yes	no	no	no
388	S17	SQUIRE Guidelines for reporting improver	Ogrinc G	Indicate that the manuscript concerns an initiativ	na	na	list	table 1	yes	no	no	no	no	no	no
389	S17	SQUIRE Guidelines for reporting improver	Ogrinc G	Provide adequate information to aid in searching	na	na	list	table 1	no	yes	no	no	no	no	no
390	S17	SQUIRE Guidelines for reporting improver	Ogrinc G	Summarise all key information from various sections of the text using the abstract format of the intended	na	na	list	table 1	no	yes	no	no	no	no	no
391	S17	SQUIRE Guidelines for reporting improver	Ogrinc G	Problem description: Nature and significance of t	na	na	list	table 1	no	no	yes	no	no	no	no
392	S17	SQUIRE Guidelines for reporting improver	Ogrinc G	Available knowledge: Summary of what is current	na	na	list	table 1	no	no	yes	no	no	no	no
393	S17	SQUIRE Guidelines for reporting improver	Ogrinc G	Rationale: Informal or formal frameworks, model	na	na	list	table 1	no	no	yes	no	no	no	no
394	S17	SQUIRE Guidelines for reporting improver	Ogrinc G	Specific aims: Purpose of the project and of this r	na	na	list	table 1	no	no	yes	no	no	no	no
395	S17	SQUIRE Guidelines for reporting improver	Ogrinc G	Context: Contextual elements considered import	na	na	list	table 1	no	no	no	yes	no	no	no
396	S17	SQUIRE Guidelines for reporting improver	Ogrinc G	Intervention(s): Description of the intervention(s)	na	na	list	table 1	no	no	no	yes	no	no	no

397	S17	SQUIRE Guidelines for reporting improver	Ogrinc G	Intervention(s): Specifics of the team involved in	na	na	list	table 1	no	no	no	yes	no	no	no
398	S17	SQUIRE Guidelines for reporting improver	Ogrinc G	Study of the intervention(s): Approach chosen fo	na	na	list	table 1	no	no	no	yes	no	no	no
399	S17	SQUIRE Guidelines for reporting improver	Ogrinc G	Study of the intervention(s): Approach used to es	na	na	list	table 1	no	no	no	yes	no	no	no
400	S17	SQUIRE Guidelines for reporting improver	Ogrinc G	Measures: Measures chosen for studying process	na	na	list	table 1	no	no	no	yes	no	no	no
401	S17	SQUIRE Guidelines for reporting improver	Ogrinc G	Measures: Description of the approach to the on	na	na	list	table 1	no	no	no	yes	no	no	no
402	S17	SQUIRE Guidelines for reporting improver	Ogrinc G	Measures: Methods employed for assessing com	na	na	list	table 1	no	no	no	yes	no	no	no
403	S17	SQUIRE Guidelines for reporting improver	Ogrinc G	Analysis: Qualitative and quantitative methods u	na	na	list	table 1	no	no	no	yes	no	no	no
404	S17	SQUIRE Guidelines for reporting improver	Ogrinc G	Analysis: Methods for understanding variation wi	na	na	list	table 1	no	no	no	yes	no	no	no
405	S17	SQUIRE Guidelines for reporting improver	Ogrinc G	Ethical considerations: Ethical aspects of implem	na	na	list	table 1	no	no	no	yes	no	no	no
406	S17	SQUIRE Guidelines for reporting improver	Ogrinc G	Initial steps of the intervention(s) and their evolu	na	na	list	table 1	no	no	no	no	yes	no	no
407	S17	SQUIRE Guidelines for reporting improver	Ogrinc G	Details of the process measures and outcomes	na	na	list	table 1	no	no	no	no	yes	no	no
408	S17	SQUIRE Guidelines for reporting improver	Ogrinc G	Contextual elements that interacted with the int	na	na	list	table 1	no	no	no	no	yes	no	no
409	S17	SQUIRE Guidelines for reporting improver	Ogrinc G	Observed associations between outcomes, interv	na	na	list	table 1	no	no	no	no	yes	no	no
410	S17	SQUIRE Guidelines for reporting improver	Ogrinc G	Unintended consequences such as unexpected b	na	na	list	table 1	no	no	no	no	yes	no	no
411	S17	SQUIRE Guidelines for reporting improver	Ogrinc G	Details about missing data.	na	na	list	table 1	no	no	no	no	yes	no	no
412	S17	SQUIRE Guidelines for reporting improver	Ogrinc G	Summary: Key findings, including relevance to th	na	na	list	table 1	no	no	no	no	no	yes	no
413	S17	SQUIRE Guidelines for reporting improver	Ogrinc G	Summary: Particular strengths of the project.	na	na	list	table 1	no	no	no	no	no	yes	no
414	S17	SQUIRE Guidelines for reporting improver	Ogrinc G	Interpretation: Nature of the association betwee	na	na	list	table 1	no	no	no	no	no	yes	no
415	S17	SQUIRE Guidelines for reporting improver	Ogrinc G	Interpretation: Comparison of results with finding	na	na	list	table 1	no	no	no	no	no	yes	no
416	S17	SQUIRE Guidelines for reporting improver	Ogrinc G	Interpretation: Impact of the project on people a	na	na	list	table 1	no	no	no	no	no	yes	no
417	S17	SQUIRE Guidelines for reporting improver	Ogrinc G	Interpretation: Reasons for any differences betw	na	na	list	table 1	no	no	no	no	no	yes	no
418	S17	SQUIRE Guidelines for reporting improver	Ogrinc G	Interpretation: Costs and strategic trade-offs, incl	na	na	list	table 1	no	no	no	no	no	yes	no
419	S17	SQUIRE Guidelines for reporting improver	Ogrinc G	Limitations: Limits to the generalisability of the w	na	na	list	table 1	no	no	no	no	no	yes	no
420	S17	SQUIRE Guidelines for reporting improver	Ogrinc G	Limitations: Factors that might have limited inter	na	na	list	table 1	no	no	no	no	no	yes	no
421	S17	SQUIRE Guidelines for reporting improver	Ogrinc G	Limitations: Efforts made to minimise and adjust	na	na	list	table 1	no	no	no	no	no	yes	no
422	S17	SQUIRE Guidelines for reporting improver	Ogrinc G	Conclusions: Usefulness of the work.	na	na	list	table 1	no	no	no	no	no	yes	no
423	S17	SQUIRE Guidelines for reporting improver	Ogrinc G	Conclusions: Sustainability.	na	na	list	table 1	no	no	no	no	no	yes	no
424	S17	SQUIRE Guidelines for reporting improver	Ogrinc G	Conclusions: Potential for spread to other contex	na	na	list	table 1	no	no	no	no	no	yes	no
425	S17	SQUIRE Guidelines for reporting improver	Ogrinc G	Conclusions: Implications for practice and for furt	na	na	list	table 1	no	no	no	no	no	yes	no
426	S17	SQUIRE Guidelines for reporting improver	Ogrinc G	Conclusions: Suggested next steps.	na	na	list	table 1	no	no	no	no	no	yes	no
427	S17	SQUIRE Guidelines for reporting improver	Ogrinc G	Funding: Sources of funding that supported this v	na	na	list	table 1	no	no	no	no	no	no	yes
452	S20	Economic evaluations of public health im	Reeves, p	Background and objectives : Provide an explicit statement of the economic evidence on the index policy or programme targeted by the implementation intervention			list	Table 6	no	no	yes	no	no	no	no
453	S20	Economic evaluations of public health im	Reeves, p	Background and objectives : Consider the policy	na	na	list	Table 6	no	no	yes	no	no	no	no
454	S20	Economic evaluations of public health im	Reeves, p	Target population andsubgroups : Include study participant characteristics relevant for informing equity considerations, e.g., the health status and 'gap' by sex, age and socio-economic status			list	Table 6	no	no	no	yes	no	no	no
455	S20	Economic evaluations of public health im	Reeves, p	Study perspective : Where possible adopt a socie	na	na	list	Table 6	no	no	no	yes	no	no	no
456	S20	Economic evaluations of public health im	Reeves, p	Choice of health outcomesand measurement of effectiveness : Depending on the known evidence of cost-effectiveness of the index policy/programme, consider measuring intermediate outcomes relevant to implementation and use economic modelling to extrapolate to the fina			list	Table 6	no	no	no	yes	no	no	no
457	S20	Economic evaluations of public health im	Reeves, p	Estimating resource use andcosts : Consider identifying, measuring and valuing resource use associated with both development and execution of implementation interventions			list	Table 6	no	no	no	yes	no	no	no
458	S20	Economic evaluations of public health im	Reeves, p	Characterising uncertaintyand heterogeneity : Consider variation in parameters relevant to implementation, e.g., reach and uptake			list	Table 6	no	no	no	yes	no	no	no
459	S20	Economic evaluations of public health im	Reeves, p	Distributional impacts : If applicable (refer item n	na	na	list	Table 6	no	no	no	yes	no	no	no
460	S21	Is reporting on interventions a weak link i	Riley, Barbara L	Integration of relevant theories	Descriptions of theories, including a	na	list	table 2 to 7	no	no	yes	yes	no	no	no
461	S21	Is reporting on interventions a weak link i	Riley, Barbara L	Combinations and sequencing/staging of interver	Descriptions of the deliberate combination of	na	list	table 2 to 7	no	no	no	yes	no	no	no
462	S21	Is reporting on interventions a weak link i	Riley, Barbara L	Coordinating and integrating intervention efforts	Descriptions of complementary interventions	na	list	table 2 to 7	no	no	no	yes	no	no	no
463	S21	Is reporting on interventions a weak link i	Riley, Barbara L	Adequate implementation:Quantitative descripti	time period	na	list	table 2 to 7	no	no	no	yes	no	no	no
464	S21	Is reporting on interventions a weak link i	Riley, Barbara L	Adequate implementation:Quantitative descripti	depth of engagement such as passi	na	list	table 2 to 7	no	no	no	yes	no	no	no
465	S21	Is reporting on interventions a weak link i	Riley, Barbara L	Adequate implementation:Quantitative descripti	depth of engagement such as passi	na	list	table 2 to 7	no	no	no	yes	no	no	no
466	S21	Is reporting on interventions a weak link i	Riley, Barbara L	Adequate implementation:Quantitative descripti	total educational time, total minute	na	list	table 2 to 7	no	no	no	yes	no	no	no
467	S21	Is reporting on interventions a weak link i	Riley, Barbara L	Adequate implementation:Quantitative descripti	direct funding or in-kind contributic	na	list	table 2 to 7	no	no	no	yes	no	no	no
468	S21	Is reporting on interventions a weak link i	Riley, Barbara L	Adequate implementation:Quantitative descripti	e.g., total number of participants, p	na	list	table 2 to 7	no	no	no	yes	no	no	no
469	S21	Is reporting on interventions a weak link i	Riley, Barbara L	Appropriate implementation: Qualitative descrip	implementing all essential compone	na	list	table 2 to 7	no	no	no	no	yes	no	no
470	S21	Is reporting on interventions a weak link i	Riley, Barbara L	Appropriate implementation: Qualitative descrip	na	na	list	table 2 to 7	no	no	no	no	yes	no	no
471	S21	Is reporting on interventions a weak link i	Riley, Barbara L	Appropriate implementation: Qualitative descrip	na	na	list	table 2 to 7	no	no	no	no	yes	no	no
472	S21	Is reporting on interventions a weak link i	Riley, Barbara L	Enabling structures and conditions	Descriptions of the creation of structures	na	list	table 2 to 7	no	no	no	yes	no	no	no
473	S21	Is reporting on interventions a weak link i	Riley, Barbara L	Adaptation to the contextual environment	Descriptions regarding the adjusting or	na	list	table 2 to 7	no	no	no	no	yes	no	no
474	S21	Is reporting on interventions a weak link i	Riley, Barbara L	Responsive to evaluation feedback	Descriptions regarding the collection and	na	list	table 2 to 7	no	no	no	no	yes	no	no
475	S21	Is reporting on interventions a weak link i	Riley, Barbara L	Sustainability	Discussion regarding the continuation or extension of	na	list	table 2 to 7	no	no	no	no	yes	yes	no
476	S22	Reporting guidelines for implementation	Yousafzai, Aisha K	Previous evidence about the intervention	(1) Briefly describe the nature and s	na	list	Table 1	no	no	yes	no	no	no	no
477	S22	Reporting guidelines for implementation	Yousafzai, Aisha K	Effectiveness of the present intervention (if know	If the effectiveness of the specific ir	na	list	Table 1	no	no	yes	no	no	no	no
478	S22	Reporting guidelines for implementation	Yousafzai, Aisha K	Rationale for the implementation research	Explain why the implementation re	na	list	Table 1	no	no	yes	no	no	no	no
479	S22	Reporting guidelines for implementation	Yousafzai, Aisha K	Aims and objectives of the implementation resea	List the aims and objectives of the i	na	list	Table 1	no	no	yes	no	no	no	no
480	S22	Reporting guidelines for implementation	Yousafzai, Aisha K	Context of implementation for the intervention	Briefly describe the setting for the i	na	list	Table 1	no	no	no	yes	no	no	no
481	S22	Reporting guidelines for implementation	Yousafzai, Aisha K	Implementation strategy for the intervention	Describe the implementation strate	na	list	Table 1	no	no	no	yes	no	no	no
482	S22	Reporting guidelines for implementation	Yousafzai, Aisha K	Implementation recipients for the intervention	Include a summary about the recipi	na	list	Table 1	no	no	no	yes	no	no	no
483	S22	Reporting guidelines for implementation	Yousafzai, Aisha K	Intended intervention content	Describe the intervention in the int	na	list	Table 1	no	no	no	yes	no	no	no
484	S22	Reporting guidelines for implementation	Yousafzai, Aisha K	Changes to intervention content	Provide information about, and exp	na	list	Table 1	no	no	no	yes	no	no	no
485	S22	Reporting guidelines for implementation	Yousafzai, Aisha K	Intended intensity/total exposure to the interven	Provide information for the interve	na	list	Table 1	no	no	no	yes	no	no	no
486	S22	Reporting guidelines for implementation	Yousafzai, Aisha K	Personnel involved in supporting the implementa	Briefly describe information about	na	list	Table 1	no	no	no	yes	no	no	no
487	S22	Reporting guidelines for implementation	Yousafzai, Aisha K	Personnel delivering the intervention (e.g., moth	(1) Describe the delivery agent for t	na	list	Table 1	no	no	no	yes	no	no	no
488	S22	Reporting guidelines for implementation	Yousafzai, Aisha K	Methods to assess fidelity regarding delivery of ir	Describe the methods and tools use	na	list	Table 1	no	no	no	yes	no	no	no
489	S22	Reporting guidelines for implementation	Yousafzai, Aisha K	Methods to assess understanding and enactmen	Describe the methods and tools use	na	list	Table 1	no	no	no	yes	no	no	no

490	S22	Reporting guidelines for implementation	Yousafzai, Aisha K	Implementation research data collection team	Describe the data collection team v	na	list	Table 1	no	no	no	yes	no	no	no
491	S22	Reporting guidelines for implementation	Yousafzai, Aisha K	Sampling and data management procedures	(1) Describe the sample size, sampli	na	list	Table 1	no	no	no	yes	no	no	no
492	S22	Reporting guidelines for implementation	Yousafzai, Aisha K	Plan of analysis for implementation data	Describe the plan of analysis. For qu	na	list	Table 1	no	no	no	yes	no	no	no
493	S22	Reporting guidelines for implementation	Yousafzai, Aisha K	Results of the implementation evaluation	Present the results for all variables	na	list	Table 1	no	no	no	no	yes	no	no
494	S22	Reporting guidelines for implementation	Yousafzai, Aisha K	Interpretation of findings of the implementation	Provide interpretation of the findin	na	list	Table 1	no	no	no	no	no	yes	no
495	S22	Reporting guidelines for implementation	Yousafzai, Aisha K	Strengths and limitations of the implementation	Describe the strengths and limitatic	na	list	Table 1	no	no	no	no	no	yes	no
496	S22	Reporting guidelines for implementation	Yousafzai, Aisha K	Scalability and sustainability of the intervention a	Consider any implications of the fin	na	list	Table 1	no	no	no	no	no	yes	no
497	S23	A reporting guide for implementation scie	CCDR	Title	Compose a title that includes the p	na	list	table 1	yes	no	no	no	no	no	no
498	S23	A reporting guide for implementation scie	CCDR	Abstract	Provide a 200 to 250-word abstract	na	list	table 1	no	yes	no	no	no	no	no
499	S23	A reporting guide for implementation scie	CCDR	Issue identification	Identify the topic of the study and v	na	list	table 1	no	no	yes	no	no	no	no
500	S23	A reporting guide for implementation scie	CCDR	What is known to date	Provide a summary of the literature	na	list	table 1	no	no	yes	no	no	no	no
501	S23	A reporting guide for implementation scie	CCDR	Rationale for study	Identify the rationale for the impler	na	list	table 1	no	no	yes	no	no	no	no
502	S23	A reporting guide for implementation scie	CCDR	Objective	State the objective of the intervent	na	list	table 1	no	no	yes	no	no	no	no
503	S23	A reporting guide for implementation scie	CCDR	Intervention:Setting/ participants	Describe the setting and populatior	na	list	table 1	no	no	no	yes	no	no	no
504	S23	A reporting guide for implementation scie	CCDR	Intervention: Ethics review if indicated	For studies involving human partici	na	list	table 1	no	no	no	yes	no	no	no
505	S23	A reporting guide for implementation scie	CCDR	Intervention: Intervention	Describe the intervention and how	na	list	table 1	no	no	no	yes	no	no	no
506	S23	A reporting guide for implementation scie	CCDR	Intervention:Outcome measures	Describe how the intervention was	na	list	table 1	no	no	no	yes	no	no	no
507	S23	A reporting guide for implementation scie	CCDR	Outcomes: Setting/ participants	Present the findings in enough deta	na	list	table 1	no	no	no	no	yes	no	no
508	S23	A reporting guide for implementation scie	CCDR	Outcomes: Primary outcomes	Present the primary outcome meas	na	list	table 1	no	no	no	no	yes	no	no
509	S23	A reporting guide for implementation scie	CCDR	Outcomes: Secondary outcomes	Provide any secondary outcome me	na	list	table 1	no	no	no	no	yes	no	no
510	S23	A reporting guide for implementation scie	CCDR	Outcomes: Intervention experience	Describe any insights that arose as	na	list	table 1	no	no	no	no	yes	no	no
511	S23	A reporting guide for implementation scie	CCDR	Summary of key findings	Summarize and interpret the key fir	na	list	table 1	no	no	no	no	no	yes	no
512	S23	A reporting guide for implementation scie	CCDR	Comparisons	Compare the results of the interver	na	list	table 1	no	no	no	no	no	yes	no
513	S23	A reporting guide for implementation scie	CCDR	Strengths and limitations	Identify the strengths and limitatio	na	list	table 1	no	no	no	no	no	yes	no
514	S23	A reporting guide for implementation scie	CCDR	Implications and next steps	Consider implications, next steps or	na	list	table 1	no	no	no	no	no	yes	no
515	S23	A reporting guide for implementation scie	CCDR	Conclusion	Ensure the conclusion integrates th	na	list	table 1	no	no	no	no	no	yes	no
516	S23	A reporting guide for implementation scie	CCDR	Illustrating key findings	When appropriate, include an illust	na	list	table 1	no	no	no	no	yes	no	no
517	S24	A guide to scaling up population health in	Milat, Andrew J	Assess effectiveness	Determine effectiveness, interventi	Assessment of scalability of tl	list	Table 1	no	no	no	yes	no	no	no
518	S24	A guide to scaling up population health in	Milat, Andrew J	Assess potential reach and adoption	Determine if the likely reach and ac	Assessment of scalability of tl	list	Table 1	no	no	no	yes	no	no	no
519	S24	A guide to scaling up population health in	Milat, Andrew J	Assess alignment with the strategic context	Determine whether the interventio	Assessment of scalability of tl	list	Table 1	no	no	yes	yes	no	no	no
520	S24	A guide to scaling up population health in	Milat, Andrew J	Assess acceptability and feasibility	Judge whether the intervention cou	Assessment of scalability of tl	list	Table 1	no	no	yes	yes	no	no	no
521	S24	A guide to scaling up population health in	Milat, Andrew J	Document a rationale for scale-up	Draw up a rationale for scaling up fi	Development of scaling strate	list	Table 1	no	no	yes	yes	no	no	no
522	S24	A guide to scaling up population health in	Milat, Andrew J	Describe the intervention	Describe 'what' will be scaled up an	Development of scaling strate	list	Table 1	no	yes	yes	yes	no	no	no
523	S24	A guide to scaling up population health in	Milat, Andrew J	Complete a situational and stakeholder analysis	Map the social, political and organi:	Development of scaling strate	list	Table 1	no	no	no	yes	no	no	no
524	S24	A guide to scaling up population health in	Milat, Andrew J	Determine who could be involved in scale-up and	Consider who might perform key fu	Development of scaling strate	list	Table 1	no	no	no	yes	no	no	no
525	S24	A guide to scaling up population health in	Milat, Andrew J	Select an approach to scaling up	There are two main approaches to	Development of scaling strate	list	Table 1	no	no	no	yes	no	no	no
526	S24	A guide to scaling up population health in	Milat, Andrew J	Consider options for evaluation and monitoring	Determine what variables are impo	Development of scaling strate	list	Table 1	no	no	no	yes	no	no	no
527	S24	A guide to scaling up population health in	Milat, Andrew J	Estimate resources required for scale-up	Estimate the human, technical and	Development of scaling strate	list	Table 1	no	no	no	yes	no	no	no
528	S24	A guide to scaling up population health in	Milat, Andrew J	Write up the scaling up plan	The plan should present a clear and	Development of scaling strate	list	Table 1	no	no	no	yes	no	no	no
529	S24	A guide to scaling up population health in	Milat, Andrew J	Consult with stakeholders	Assess the appropriateness and acc	preparing for scaling up	list	Table 1	no	no	no	yes	no	no	no
530	S24	A guide to scaling up population health in	Milat, Andrew J	Legitimise change	Gain the support of decision maker	preparing for scaling up	list	Table 1	no	no	no	yes	no	no	no
531	S24	A guide to scaling up population health in	Milat, Andrew J	Build a constituency	Mobilise the broader 'community o	preparing for scaling up	list	Table 1	no	no	no	yes	no	no	no
532	S24	A guide to scaling up population health in	Milat, Andrew J	Realign and mobilise resources	Mobilise financial resources throug	preparing for scaling up	list	Table 1	no	no	no	yes	no	no	no
533	S24	A guide to scaling up population health in	Milat, Andrew J	Modify and strengthen organisations	When scaling up interventions, mos	Implementation of the strate	list	Table 1	no	no	no	yes	no	no	no
534	S24	A guide to scaling up population health in	Milat, Andrew J	Coordinate action and governance	Develop and implement concrete a	Implementation of the strate	list	Table 1	no	no	no	yes	no	no	no
535	S24	A guide to scaling up population health in	Milat, Andrew J	Monitor performance and efficiency	Develop systems that have an ongo	Implementation of the strate	list	Table 1	no	no	no	yes	no	no	no
536	S24	A guide to scaling up population health in	Milat, Andrew J	Ensure sustainability	Implement organisational and cultu	Implementation of the strate	list	Table 1	no	no	no	no	no	yes	no
537	S25	Standards for Reporting Implementation S	Pinnock, Hilary	Identification as an implementation study, and de	In addition to specifying the study c	na	list	Table 1 (explanatio	yes	no	no	no	no	no	no
538	S25	Standards for Reporting Implementation S	Pinnock, Hilary	Identification as an implementation study, includ	For clarity of indexing and identifica	na	list	Table 1 (explanatio	no	yes	no	no	no	no	no
539	S25	Standards for Reporting Implementation S	Pinnock, Hilary	Description of the problem, challenge, or deficier	Identifying and characterising the p	na	list	Table 1 (explanatio	no	no	yes	no	no	no	no
540	S25	Standards for Reporting Implementation S	Pinnock, Hilary	The scientific background and rationale for the im[illegible]	Authors of implementation studies need to explain the [illegible] being implemented:	[illegible]	[illegible]	[illegible]	[illegible]	[illegible]	[illegible]	[illegible]	[illegible]	no	no
541	S25	Standards for Reporting Implementation S	Pinnock, Hilary	The scientific background and rationale for the in	Authors of implementation studies need to explain the rationale for the choice of implementation strategy and for the validity of the intervention being implemented:	[illegible]	[illegible]	Table 1 (explanatio[illegible]	[illegible]	no	yes	no	[illegible]	no	no
542	S25	Standards for Reporting Implementation S	Pinnock, Hilary	The aims of the study, differentiating between im	The aims and objectives should dist	na	list	Table 1 (explanatio	no	no	yes	no	no	no	no
543	S25	Standards for Reporting Implementation S	Pinnock, Hilary	The design and key features of the evaluation (cr	The study design should be identifie	na	list	Table 1 (explanatio	no	no	no	yes	no	no	no
544	S25	Standards for Reporting Implementation S	Pinnock, Hilary	The context in which the intervention was impler	Successful implementation of evidence into practice is a planned facilitated process involving the interplay between individuals, evidence and context to promote evidence-informed practice. A rich description of the context is cr	[illegible]	[illegible]	[illegible]	[illegible]	no	no	yes	no	no	no
545	S25	Standards for Reporting Implementation S	Pinnock, Hilary	The characteristics of the targeted "site(s)" (locat	Recruitment is considered at two le	na	list	Table 1 (explanatio	no	no	no	yes	no	no	no
546	S25	Standards for Reporting Implementation S	Pinnock, Hilary	The population targeted by the intervention and any eligibility criteria.	Recruitment is considered at two le	na	list	Table 1 (explanatio	no	no	no	yes	no	no	no
547	S25	Standards for Reporting Implementation S	Pinnock, Hilary	A description of the implementation strategy	Descriptions of implementation strategies and complex interventions are criticised as being inconsistently labelled, poorly described, rarely justified, not easy to understand15 46 47 and not sufficiently detailed to enable the inte	[illegible]	[illegible]	[illegible]	[illegible]	no	no	yes	no	no	no
548	S25	Standards for Reporting Implementation S	Pinnock, Hilary	A description of the intervention	Descriptions of implementation strategies and complex interventions are criticised as being inconsistently labelled, poorly described, rarely justified, not easy to understand15 46 47 and not sufficiently detailed to enable the inte	[illegible]	[illegible]	[illegible]	[illegible]	no	no	yes	no	no	no
549	S25	Standards for Reporting Implementation S	Pinnock, Hilary	Any subgroups recruited for additional research t	Typically in implementation studies	na	list	Table 1 (explanatio	no	no	no	yes	no	no	no
550	S25	Standards for Reporting Implementation S	Pinnock, Hilary	Defined pre-specified primary and other outcome	Figure 2 illustrates the outcomes relevant to implementation science and the StaRI checklist items to which they relate. This schema borrows from the conceptual models and taxonomy of outcomes described by Proctor et al,16	[illegible]	[illegible]	[illegible]	[illegible]	no	no	yes	no	no	no
551	S25	Standards for Reporting Implementation S	Pinnock, Hilary	Defined pre-specified primary and other outcome[illegible]	Figure 2 illustrates the outcomes [illegible] to which they relate. This schema borrows from the conceptual models and taxonomy of outcomes described by Proctor et al,16	[illegible]	[illegible]	[illegible]	[illegible]	no	no	yes	no	no	no
552	S25	Standards for Reporting Implementation S	Pinnock, Hilary	Process evaluation objectives and outcomes relat	A process evaluation (or formative	na	list	Table 1 (explanatio	no	no	no	yes	no	no	no
553	S25	Standards for Reporting Implementation S	Pinnock, Hilary	Methods for resource use, costs, economic outcomes, and analysis for the implementation strategy	[illegible]		list	Table 1 (explanatio	no	no	no	yes	no	no	no
554	S25	Standards for Reporting Implementation S	Pinnock, Hilary	Methods for resource use, costs, economic outcc	Economic evaluation can inform fut	na	list	Table 1 (explanatio	no	no	no	yes	no	no	no
555	S25	Standards for Reporting Implementation S	Pinnock, Hilary	Rationale for sample sizes (including sample size	It is important to recruit sufficient p	na	list	Table 1 (explanatio	no	no	no	yes	no	no	no
556	S25	Standards for Reporting Implementation S	Pinnock, Hilary	Methods of analysis (with reasons for that choice	Design-specific advice on reporting	na	list	Table 1 (explanatio	no	no	no	yes	no	no	no
557	S25	Standards for Reporting Implementation S	Pinnock, Hilary	Any a priori subgroup analyses (such as between	Subgroups should be specified a pri	na	list	Table 1 (explanatio	no	no	no	yes	no	no	no
558	S25	Standards for Reporting Implementation S	Pinnock, Hilary	Proportion recruited and characteristics of the re	As in cluster RCTs, the populations need to be considered at two levels:	[illegible]	[illegible]	Table 1 (explanatio	no	no	no	no	yes	no	no

559	S25	Standards for Reporting Implementation S	Pinnock, Hilary	Proportion recruited and characteristics (if approp	As in other RCTs, the population needs to be considered at two levels:	[illegible]	list	Table 1 (explanatio	no	no	no	no	yes	no	no
560	S25	Standards for Reporting Implementation S	Pinnock, Hilary	Primary and other outcome(s) of the implementa	We suggest that the primary and ot	na	list	Table 1 (explanatio	no	no	no	no	yes	no	no
561	S25	Standards for Reporting Implementation S	Pinnock, Hilary	Primary and other outcome(s) of the intervention (if assessed)	We suggest that the primary and ot	na	list	Table 1 (explanatio	no	no	no	no	yes	no	no
562	S25	Standards for Reporting Implementation S	Pinnock, Hilary	Process data related to the implementation strat	Process evaluation should be relate	na	list	Table 1 (explanatio	no	no	no	no	yes	no	no
563	S25	Standards for Reporting Implementation S	Pinnock, Hilary	Resource use, costs, economic outcomes, and ana	Reporting of economic results should adhere to existing relevant guidelines.71 72 It should be clear whether the economic results relate to the implementation strategy, the intervention that is being implemented or both. Repor	[illegible]	[illegible]	[illegible]	[illegible]	no	no	[illegible]	yes	no	no
564	S25	Standards for Reporting Implementation S	Pinnock, Hilary	Resource use, costs, economic outcomes, and an	Reporting of economic results should adhere to existing relevant guidelines.71 72 It should be clear whether the economic results relate to the implementation strategy, the intervention that is being implemented or both. Repor	na	[illegible]	[illegible]	[illegible]	no	no	[illegible]	yes	no	no
565	S25	Standards for Reporting Implementation S	Pinnock, Hilary	Representativeness and outcomes of subgroups i	Subgroup analyses should be disting	na	list	Table 1 (explanatio	no	no	no	no	yes	no	no
566	S25	Standards for Reporting Implementation S	Pinnock, Hilary	Fidelity to implementation strategy as planned ar	Fidelity may be considered at two le	na	list	Table 1 (explanatio	no	no	no	no	yes	no	no
567	S25	Standards for Reporting Implementation S	Pinnock, Hilary	Fidelity to delivering the core components of intervention (where measured)	Fidelity may be considered at two le	na	list	Table 1 (explanatio	no	no	no	no	yes	no	no
568	S25	Standards for Reporting Implementation S	Pinnock, Hilary	Contextual changes (if any) which may have affec	There should be a description of an	na	list	Table 1 (explanatio	no	no	no	no	yes	no	no
569	S25	Standards for Reporting Implementation S	Pinnock, Hilary	All important harms or unintended effects in eacl	Adverse or unintended consequenc	na	list	Table 1 (explanatio	no	no	no	no	yes	no	no
570	S25	Standards for Reporting Implementation S	Pinnock, Hilary	Summary of findings, strengths and limitations, c	The structure of the discussion will	na	list	Table 1 (explanatio	no	no	no	no	no	yes	no
571	S25	Standards for Reporting Implementation S	Pinnock, Hilary	Discussion of policy, practice and/or research imp	The authors should reflect on:1. The implications of the success (or otherwise) of the implementation strategy, for research and practice. 2. The health benefits (or otherwise) of implementing the intervention.	[illegible]	list	Table 1 (explanatio	[illegible]	no	no	no	no	yes	no
572	S25	Standards for Reporting Implementation S	Pinnock, Hilary	Discussion of policy, practice and/or research implications of the intervention (specifically including sustainability)	The authors should reflect on:1. The implications of the success (or otherwise) of the implementation strategy, for research and practice. 2. The health benefits (or otherwise) of implementing the intervention.	[illegible]	list	Table 1 (explanatio	[illegible]	no	no	no	no	yes	no
573	S25	Standards for Reporting Implementation S	Pinnock, Hilary	Include statement(s) on regulatory approvals (inc	Ethical considerations, regulatory a	na	list	Table 1 (explanatio	no	no	no	no	no	no	yes
574	S26	Guide for Monitoring Scale-up of Health P	Adamou, Bridgit	Define the Innovation and the Objectives and Sco	Think about the multisector, global,	Development of scaling strate	Text	Page 12 and table 1	no	no	no	no	no	no	no
575	S26	Guide for Monitoring Scale-up of Health P	Adamou, Bridgit	Create a Framework	Develop a logic model	Development of scaling strate	Text	Page 15 and table :	no	no	no	yes	no	no	no
576	S26	Guide for Monitoring Scale-up of Health P	Adamou, Bridgit	Identify Necessary Resources to Implement the M	dentify and gather the necessary M	preparing for scaling up	Text	Page 19 and table :	no	no	no	yes	no	no	no
577	S26	Guide for Monitoring Scale-up of Health P	Adamou, Bridgit	Select Key Indicators	Select indicators and create operati	Development of scaling strate	Text	Page 21 and table :	no	no	no	yes	no	no	no
578	S26	Guide for Monitoring Scale-up of Health P	Adamou, Bridgit	Establish Data Sources and Reporting Systems	Determine the data sources	Development of scaling strate	Text	Page 25 and table :	no	no	no	yes	no	no	no
579	S26	Guide for Monitoring Scale-up of Health P	Adamou, Bridgit	Develop a Data Use and Dissemination Plan	Decide how and to which audience:	Development of scaling strate	Text	Page 30 and table :	no	no	no	yes	no	no	no
580	S26	Guide for Monitoring Scale-up of Health P	Adamou, Bridgit	Collect Data	Create a database and enter in data	Implementation of the strate	Text	Page 31 and table :	no	no	no	yes	no	no	no
581	S26	Guide for Monitoring Scale-up of Health P	Adamou, Bridgit	Analyze Data and Determine if Scale-up Is Progre	Conduct data analysis	Implementation of the strate	Text	Page 31 and table 1	no	yes	no	yes	no	no	no
582	S26	Guide for Monitoring Scale-up of Health P	Adamou, Bridgit	Make Program Adjustments Based on Findings ar	Feed the information back to the pr	Implementation of the strate	Text	Page 32 and table :	no	no	no	no	no	no	no
583	S26	Guide for Monitoring Scale-up of Health P	Adamou, Bridgit	Continue the Monitoring and Evaluation Process	Make continuous adjustments and	Evaluation and monitoring	Text	Page 33 and table :	no	no	no	yes	no	no	no
584	S27	Evaluating the scale-up for maternal and c	Bryce, Jennifer	Policies and planning : Are the interventions and	Systematic feedback to program im	Assessment of scalability of th	list	figure 2	no	no	yes	no	no	no	no
585	S27	Evaluating the scale-up for maternal and c	Bryce, Jennifer	Provision : Are adequate services being provided?	Systematic feedback to program im	Development of scaling strate	list	figure 2	no	no	no	yes	no	no	no
586	S27	Evaluating the scale-up for maternal and c	Bryce, Jennifer	Utilization : Are these services being used by the	Systematic feedback to program im	Development of scaling strate	list	figure 2	no	no	yes	yes	no	no	no
587	S27	Evaluating the scale-up for maternal and c	Bryce, Jennifer	Effective cove rage : Have adequate levels of effective coverage	Systematic feedback to program im	Evaluation and monitoring	list	figure 2	no	no	no	yes	yes	no	no
588	S27	Evaluating the scale-up for maternal and c	Bryce, Jennifer	Impact : Is there an impact on health and nutritio	Systematic feedback to program im	Evaluation and monitoring	list	figure 2	no	no	no	yes	yes	no	no
589	S27	Evaluating the scale-up for maternal and c	Bryce, Jennifer	Cost-effectiveness : Is the program good value fo	na	Evaluation and monitoring	list	figure 2	no	no	no	yes	yes	yes	no
590	S27	Evaluating the scale-up for maternal and c	Bryce, Jennifer	Learning & capacity building : Do key stakeholder	na	preparing for scaling up	list	figure 2	no	no	no	no	no	yes	no
591	S28	Every Newborn: health-systems bottlenec	Dickson, Kim E	Step 1: Assess thesituation, determinepriorities based on	[illegible]	Development of scaling strate	list	Figure 2 page 451	no	no	no	yes	no	no	no
592	S28	Every Newborn: health-systems bottlenec	Dickson, Kim E	Step 2: Seizeopportunitieswithin theconstraints o	na	Development of scaling strate	list	Figure 2 page 451	no	no	no	yes	no	no	no
593	S28	Every Newborn: health-systems bottlenec	Dickson, Kim E	Step 3:Systematicallyscale up care	na	Implementation of the strate	list	Figure 2 page 451	no	no	no	yes	no	no	no
594	S28	Every Newborn: health-systems bottlenec	Dickson, Kim E	Step 4: Monitorcoverage, measureeffect and cost	na	Evaluation and monitoring	list	Figure 2 page 451	no	no	no	yes	no	no	no
595	S29	Scaling science	McLean, Robert	guiding principles for scaling impact: Justification	Justification encourages that scaling is governed by accountability to those who will be impacted by scaling.	Assessment of scalability of th	Text	Page 34 (scaling im	no	no	yes	yes	no	no	no
596	S29	Scaling science	McLean, Robert	guiding principles for scaling impact: Optimal Sca	Scaling will nearly always imply trade-offs. The search for optimal impact—not maximum impact—governs scaling toward balanced and judicious results,	Development of scaling strate	Text	Page 52 (scaling im	[illegible]	yes	no	yes	no	no	no
597	S29	Scaling science	McLean, Robert	guiding principles for scaling impact: Coordinatio	Scaling impact for the public good requires a dynamic mix of relationships. Coordination is crucial in designing, engaging and adapting within this system	preparing for scaling up	Text	Page 65 (scaling im	[illegible]	no	no	yes	no	no	no
598	S29	Scaling science	McLean, Robert	guiding principles for scaling impact: Dynamic Eva	Dynamic Evaluation encourages that evaluation underpins scaling from start to finish	Evaluation and monitoring	Text	Page 82 (scaling im	no	no	no	yes	no	no	no
599	S29	Scaling science	McLean, Robert	framing: Focus and questions: Based on the resea	na	Development of scaling strate	list	illustration 6 part 3	no	no	yes	yes	no	no	no
600	S29	Scaling science	McLean, Robert	framing: Focus and questions: Which users and b	na	Development of scaling strate	list	illustration 6 part 3	no	no	yes	yes	no	no	no
601	S29	Scaling science	McLean, Robert	framing: Focus and questions:Who/what are the	na	Development of scaling strate	list	illustration 6 part 3	no	yes	yes	yes	no	no	no
602	S29	Scaling science	McLean, Robert	doing: Data collection, analysis/synthesis, and int	na	Development of scaling strate	list	illustration 6 part 3	no	no	no	yes	no	yes	no
603	S29	Scaling science	McLean, Robert	doing: Data collection, analysis/synthesis, and int	na	Implementation of the strate	list	illustration 6 part 3	no	no	no	yes	no	no	no
604	S29	Scaling science	McLean, Robert	doing: Data collection, analysis/synthesis, and int	na	Implementation of the strate	list	illustration 6 part 3	no	no	no	yes	no	no	no
605	S29	Scaling science	McLean, Robert	sharing: Communicating research results: What s	na	Development of scaling strate	list	illustration 6 part 3	no	no	no	yes	no	no	no
606	S29	Scaling science	McLean, Robert	sharing: Communicating research results: Are the	na	Implementation of the strate	list	illustration 6 part 3	no	no	no	no	no	yes	no
607	S30	Pathways for scaling up public health inte	Indig, Devon	development : Was the program developed from	na	Development of scaling strate	list	figure 2	no	no	no	no	no	no	no
608	S30	Pathways for scaling up public health inte	Indig, Devon	efficacy testing : was a pilot test of the program c	na	Assessment of scalability of th	list	figure 2	no	no	yes	yes	no	no	no
609	S30	Pathways for scaling up public health inte	Indig, Devon	real world trial : was a larger scale trial conducted	na	Implementation of the strate	list	figure 2	no	no	yes	yes	no	no	no
610	S30	Pathways for scaling up public health inte	Indig, Devon	Dissemination : was there large scale disseminat	na	Implementation of the strate	list	figure 2	no	no	no	yes	no	yes	no
611	S30	Pathways for scaling up public health inte	Indig, Devon	Dissemination : Was the program integrated into	na	Implementation of the strate	list	figure 2	no	no	no	yes	no	no	no
612	S30	Pathways for scaling up public health inte	Indig, Devon	Dissemination : Was the program replicated, ada	na	Implementation of the strate	list	figure 2	no	no	no	yes	no	yes	no
613	S31	Beginning with the end in mind: planning	Simmons, Ruth	Engage in a participatory process involving keysta	Is input about the project being sou	Development of scaling strate	Text	page 3	no	no	yes	yes	no	no	no
614	S31	Beginning with the end in mind: planning	Simmons, Ruth	Ensure the relevance of the proposed innovation	Does the innovation address a persistent health or service delivery	[illegible]	Text	page 3	no	no	yes	no	no	no	no
615	S31	Beginning with the end in mind: planning	Simmons, Ruth	Reach consensus on expectations for scale-up	Is the project being designed in ligh	Development of scaling strate	Text	page4	no	no	yes	yes	no	no	no
616	S31	Beginning with the end in mind: planning	Simmons, Ruth	Tailor the innovation to the sociocultural and inst	Has the project identified and taken into consideration	[illegible]	Text	page 5	no	no	yes	yes	no	no	no
617	S31	Beginning with the end in mind: planning	Simmons, Ruth	Keep the innovation as simple as possible	Has the package of interventions been kept as simple as	[illegible]	Text	page 5	no	no	no	yes	no	no	no
618	S31	Beginning with the end in mind: planning	Simmons, Ruth	Test the innovation in the variety of sociocultural	Is the innovation being tested in the variety of sociocultural	[illegible]	Text	page 6	no	no	no	no	no	no	no
619	S31	Beginning with the end in mind: planning	Simmons, Ruth	Test the innovation under the routine operating c	Does the innovation being tested require human and financial resources that can reasonably be expected to be available during scale-up?	[illegible]	[illegible]	[illegible]	[illegible]	[illegible]	yes	yes	no	no	no
620	S31	Beginning with the end in mind: planning	Simmons, Ruth	Develop plans to assess and document the proce	Are appropriate steps being taken t	Development of scaling strate	Text	page 7	no	no	no	yes	no	no	no
621	S31	Beginning with the end in mind: planning	Simmons, Ruth	Advocate with donors and other sources of fundi	Is there provision for early and cont	Development of scaling strate	Text	page 8	no	no	no	yes	no	no	no
622	S31	Beginning with the end in mind: planning	Simmons, Ruth	Prepare to advocate for necessary changes in pol	Are there plans to advocate for cha	Development of scaling strate	Text	page 8	no	no	no	yes	no	no	no
623	S31	Beginning with the end in mind: planning	Simmons, Ruth	Develop plans for how to promote learning and d	Does the project design include mechanisms to review progress and incorporate new learning into the implementation process?	[illegible]	[illegible]	[illegible]	[illegible]	[illegible]	yes	yes	no	no	no
624	S31	Beginning with the end in mind: planning	Simmons, Ruth	Plan on being cautious about initiating scale-up b	Is there a shared understanding am	Development of scaling strate	Text	page 9	no	no	no	no	no	no	no
625	S32	The MAPS toolkit: mHealth assessment ar	WHO	Groundwork: Parameters of scale: Growth (client	Increase in number of users or clien	Development of scaling strate	LIST	p3, figure3, p11	no	no	no	yes	yes	no	no
626	S32	The MAPS toolkit: mHealth assessment ar	WHO	Groundwork: Parameters of scale:Adoption (insti	Increase in number of institutions tl	Development of scaling strate	LIST	p3, figure3, p11	no	no	no	yes	yes	no	no
627	S32	The MAPS toolkit: mHealth assessment ar	WHO	Groundwork: Parameters of scale: Expansion (gec	Increase in number of districts, regi	Development of scaling strate	LIST	p3, figure3, p11	no	no	no	yes	yes	no	no

628	S32	The MAPS toolkit: mHealth assessment ar	WHO	Groundwork: Contexual environment	Conducting a thorough assessment	Assessment of scalability of th	LIST	p3, figure3, p11	no	no	yes	yes	no	no	no
629	S32	The MAPS toolkit: mHealth assessment ar	WHO	Groundwork: Scientific basis	Evidence that your project team ha	Development of scaling strate	LIST	p3, figure3, p12	no	no	yes	yes	no	no	no
630	S32	The MAPS toolkit: mHealth assessment ar	WHO	Parnerships: Strategic engagement: Identificatior	that have the diversity of expertise	preparing for scaling up	LIST	p3, figure3, p25	no	no	no	yes	no	no	no
631	S32	The MAPS toolkit: mHealth assessment ar	WHO	Parnerships: Strategic engagement: Fostering buy	Assessing each potential partner's r	preparing for scaling up	LIST	p3, figure3, p25	no	no	no	yes	no	no	no
632	S32	The MAPS toolkit: mHealth assessment ar	WHO	Parnerships: Partnership sustainability:cultivating	na	preparing for scaling up	LIST	p3, figure3, p26	no	no	no	yes	no	no	no
633	S32	The MAPS toolkit: mHealth assessment ar	WHO	Parnerships: Partnership sustainability:establishn	na	preparing for scaling up	LIST		no	no	no	yes	no	yes	no
634	S32	The MAPS toolkit: mHealth assessment ar	WHO	Financial health: Financial management: Program	The project team should differentia	Implementation of the strate	LIST	p3, figure3, p37	no	no	no	no	no	no	no
635	S32	The MAPS toolkit: mHealth assessment ar	WHO	Financial health: Financial management: End-use	the cost incurred by the user, which	Implementation of the strate	LIST	p3, figure3, p37	no	yes	no	yes	yes	no	no
636	S32	The MAPS toolkit: mHealth assessment ar	WHO	Financial health: Financial management: Health s	na	Implementation of the strate	LIST	p3, figure3, p37	no	yes	no	yes	yes	no	no
637	S32	The MAPS toolkit: mHealth assessment ar	WHO	Financial health: Financial management: Forecast	the economic costs of scaling up	Implementation of the strate	LIST	p3, figure3, p37	no	no	no	yes	yes	no	no
638	S32	The MAPS toolkit: mHealth assessment ar	WHO	Financial health: Financial model:Value chain ana	exploring and documenting the inte	Implementation of the strate	LIST	p3, figure3, p38	no	no	no	yes	no	no	no
639	S32	The MAPS toolkit: mHealth assessment ar	WHO	Financial health: Financial model:Business plan	na	Implementation of the strate	LIST	p3, figure3, p38	no	no	no	yes	no	no	no
640	S32	The MAPS toolkit: mHealth assessment ar	WHO	Financial health: Financial model:Sustainability of	required to seek out and secure diff	Implementation of the strate	LIST		no	no	no	no	no	yes	no
641	S32	The MAPS toolkit: mHealth assessment ar	WHO	Technology and architecture: Data: Data access a	na	Implementation of the strate	LIST	p48	no	no	no	yes	no	no	no
642	S32	The MAPS toolkit: mHealth assessment ar	WHO	Technology and architecture: Data: Data transmis	na	Implementation of the strate	LIST	p48	no	no	no	yes	no	no	no
643	S32	The MAPS toolkit: mHealth assessment ar	WHO	Technology and architecture: Data: Data security	na	Implementation of the strate	LIST	p48	no	no	no	yes	no	no	no
644	S32	The MAPS toolkit: mHealth assessment ar	WHO	Technology and architecture: Interoperability: Sy	na	Implementation of the strate	LIST	p48	no	no	no	yes	no	no	no
645	S32	The MAPS toolkit: mHealth assessment ar	WHO	Technology and architecture: Interoperability: Da	na	Implementation of the strate	LIST	p48	no	no	no	yes	no	no	no
646	S32	The MAPS toolkit: mHealth assessment ar	WHO	Technology and architecture: Adaptability: Adapt	na	Implementation of the strate	LIST	p50	no	no	yes	yes	no	no	no
647	S32	The MAPS toolkit: mHealth assessment ar	WHO	Technology and architecture: Adaptability: Adapt	na	Implementation of the strate	LIST	p50	no	no	yes	yes	no	no	no
648	S32	The MAPS toolkit: mHealth assessment ar	WHO	Technology and architecture: Adaptability: Transl	describes the capacity of an mHealt	Implementation of the strate	LIST	p50	no	no	yes	yes	no	no	no
649	S32	The MAPS toolkit: mHealth assessment ar	WHO	Operations: Personnel: Workforce development	na	Implementation of the strate	LIST	p62	no	no	no	yes	no	no	no
650	S32	The MAPS toolkit: mHealth assessment ar	WHO	Operations: Personnel:Leadership	na	Implementation of the strate	LIST	p62	no	no	no	yes	no	no	no
651	S32	The MAPS toolkit: mHealth assessment ar	WHO	Operations: Training & support: User training	na	Implementation of the strate	LIST	p62	no	no	no	yes	no	no	no
652	S32	The MAPS toolkit: mHealth assessment ar	WHO	Operations: Training & support: Supervision	na	Implementation of the strate	LIST	p62	no	no	no	yes	no	no	no
653	S32	The MAPS toolkit: mHealth assessment ar	WHO	Operations: Training & support: User and technic	na	Implementation of the strate	LIST	p62	no	no	no	yes	no	no	no
654	S32	The MAPS toolkit: mHealth assessment ar	WHO	Operations: Outreach & sensitization: Stakeholde	na	Implementation of the strate	LIST	p62	no	no	no	yes	no	no	no
655	S32	The MAPS toolkit: mHealth assessment ar	WHO	Operations: Outreach & sensitization: Community	na	Implementation of the strate	LIST	p62	no	no	no	yes	no	no	no
656	S32	The MAPS toolkit: mHealth assessment ar	WHO	Operations: Contingency planning: Technical con	na	Implementation of the strate	LIST	p62	no	no	no	yes	no	no	no
657	S32	The MAPS toolkit: mHealth assessment ar	WHO	Operations: Contingency planning: Retention of c	na	Implementation of the strate	LIST	p62	no	no	no	yes	no	no	no
658	S32	The MAPS toolkit: mHealth assessment ar	WHO	Monitoring and evaluation: Process monitoring: F	PM: Routine and ongoing monitorir	Evaluation and monitoring	LIST	p76	no	no	no	yes	yes	no	no
659	S32	The MAPS toolkit: mHealth assessment ar	WHO	Monitoring and evaluation: Process monitoring: (	na	Evaluation and monitoring	LIST	p76	no	no	no	yes	yes	no	no
660	S32	The MAPS toolkit: mHealth assessment ar	WHO	Monitoring and evaluation: Evaluation research:	na	Evaluation and monitoring	LIST	p76	no	no	no	yes	yes	no	no
661	S32	The MAPS toolkit: mHealth assessment ar	WHO	Monitoring and evaluation: Evaluation research:	na	Evaluation and monitoring	LIST	p76	no	no	no	yes	no	no	no
662	S32	The MAPS toolkit: mHealth assessment ar	WHO	Monitoring and evaluation: Evaluation research:	na	Evaluation and monitoring	LIST	p76	no	no	no	yes	no	yes	no
663	S33	Ten dimensions of scaling up reproductive	US Aggency for Inter	question of change: How do we know when we h	Is there enough capacity to scale up	Development of scaling strate	list	box page 1	no	no	yes	yes	no	no	no
664	S33	Ten dimensions of scaling up reproductive	US Aggency for Inter	A question of capacity: What management, techn[illegible]	[illegible]	Development of scaling strate	list	box page 1	no	no	no	yes	no	no	no
665	S33	Ten dimensions of scaling up reproductive	US Aggency for Inter	A question of strategy: What strategies most effe	How does scaling up occur? Replica	Development of scaling strate	list	box page 1	no	no	no	no	no	no	no
666	S33	Ten dimensions of scaling up reproductive	US Aggency for Inter	A question of impact: How should the desired im	The impact of a scaled-up program:	Development of scaling strate	list	box page 1	no	no	no	yes	no	no	no
667	S33	Ten dimensions of scaling up reproductive	US Aggency for Inter	A question of sustainability: How do we maintain	What does sustainability mean in re	Development of scaling strate	list	box page 1	no	no	no	no	no	no	no
668	S33	Ten dimensions of scaling up reproductive	US Aggency for Inter	A question of access: What kind of coverage is en	Breadth; Depth	Development of scaling strate	list	box page 1	no	no	no	yes	no	no	no
669	S33	Ten dimensions of scaling up reproductive	US Aggency for Inter	A question of supply and demand: What is being	Recognizing demand and supply co	Development of scaling strate	list	box page 1	no	yes	yes	yes	no	no	no
670	S33	Ten dimensions of scaling up reproductive	US Aggency for Inter	A question of cost: How much will it cost to scale	Estimating start-up costs; Estimatin	Development of scaling strate	list	box page 1	no	no	no	yes	no	no	no
671	S33	Ten dimensions of scaling up reproductive	US Aggency for Inter	A question of resources: What resources are nee	Financing scaling up: How much for	Development of scaling strate	list	box page 1	no	no	no	yes	no	no	no
672	S33	Ten dimensions of scaling up reproductive	US Aggency for Inter	A question of timing: When is the right time to sc	Is the technology compatible with c	Development of scaling strate	list	box page 1	no	no	yes	yes	no	no	no
673	S34	Scaling up health service delivery: from pi	Simmons, Ruth	Innovation	health interventions and/or other p	Development of scaling strate	list	Figure 1.1 (Scaling u	no	yes	yes	yes	yes	yes	no
674	S34	Scaling up health service delivery: from pi	Simmons, Ruth	User organization	the institution(s) or organization(s)	Development of scaling strate	list	Figure 1.1 (Scaling u	no	yes	yes	yes	no	yes	no
675	S34	Scaling up health service delivery: from pi	Simmons, Ruth	Environment	conditions and institutions which ar	Assessment of scalability of th	list	Figure 1.1 (Scaling u	no	no	no	yes	no	no	no
676	S34	Scaling up health service delivery: from pi	Simmons, Ruth	Resource team	individuals and organizations that s	preparing for scaling up	list	Figure 1.1 (Scaling u	no	no	no	yes	no	no	no
677	S34	Scaling up health service delivery: from pi	Simmons, Ruth	Scaling-up strategy	plans and actions necessary to fully	Development of scaling strate	list	Figure 1.1 (Scaling u	no	yes	no	yes	no	no	no
678	S34	Scaling up health service delivery: from pi	Simmons, Ruth	the type of scaling up : vertical scaling up	institutionalization through policy, p	Development of scaling strategy		(Practical guidance	no	yes	no	yes	no	no	no
679	S34	Scaling up health service delivery: from pi	Simmons, Ruth	the type of scaling up : horizontal scaling up (expa	different geographic sites or can be	Development of scaling strategy		(Practical guidance	no	yes	no	yes	no	no	no
680	S34	Scaling up health service delivery: from pi	Simmons, Ruth	the type of scaling up : diversification	Diversification, also called functiona	Development of scaling strategy		(Practical guidance	no	yes	no	yes	no	no	no
681	S34	Scaling up health service delivery: from pi	Simmons, Ruth	the type of scaling up : spontaneous scaling up	refers to diffusion of the innovation	Development of scaling strategy		(Practical guidance	no	no	no	yes	no	no	no
682	S34	Scaling up health service delivery: from pi	Simmons, Ruth	Strategic choice: dissemination and advocacy: pe	na	Development of scaling strategy		(Practical guidance	no	no	no	yes	no	no	no
683	S34	Scaling up health service delivery: from pi	Simmons, Ruth	Strategic choice: dissemination and advocacy:imp	na	Development of scaling strategy		(Practical guidance	no	no	no	yes	no	yes	no
684	S34	Scaling up health service delivery: from pi	Simmons, Ruth	Strategic choice: the organizational process: scop	na	Development of scaling strategy		(Practical guidance	no	no	no	yes	no	no	no
685	S34	Scaling up health service delivery: from pi	Simmons, Ruth	Strategic choice: the organizational process: pac	na	Development of scaling strategy		(Practical guidance	no	no	no	yes	no	no	no
686	S34	Scaling up health service delivery: from pi	Simmons, Ruth	Strategic choice: the organizational process: num	na	Development of scaling strategy		(Practical guidance	no	no	no	no	no	no	no
687	S34	Scaling up health service delivery: from pi	Simmons, Ruth	Strategic choice: the organizational process: cent	na	Development of scaling strategy		(Practical guidance	no	no	no	yes	no	no	no
688	S34	Scaling up health service delivery: from pi	Simmons, Ruth	Strategic choice: the organizational process: adap	na	Development of scaling strategy		(Practical guidance	no	no	no	yes	no	no	no
689	S34	Scaling up health service delivery: from pi	Simmons, Ruth	Strategic choice: the organizational process: parti	na	Development of scaling strategy		(Practical guidance	no	no	no	yes	no	no	no
690	S34	Scaling up health service delivery: from pi	Simmons, Ruth	Strategic choice: costs and resource mobilization	na	preparing for scaling up		(Practical guidance	no	no	no	yes	no	no	no
691	S34	Scaling up health service delivery: from pi	Simmons, Ruth	Strategic choice: costs and resource mobilization	na	preparing for scaling up		(Practical guidance	no	no	no	yes	no	no	no
692	S34	Scaling up health service delivery: from pi	Simmons, Ruth	Strategic choice: costs and resource mobilization	na	preparing for scaling up		(Practical guidance	no	no	no	yes	no	no	no
693	S34	Scaling up health service delivery: from pi	Simmons, Ruth	Strategic choice: monitoring and evaluation: indi	examples: extent to which essential features of the innovation (e.g. training, management, facility construction) are being implemented;	Evaluation and monitoring		(Practical guidance	[illegible]	no	no	yes	yes	no	no
694	S34	Scaling up health service delivery: from pi	Simmons, Ruth	Strategic choice: monitoring and evaluation: indi	Examples: number of sites implementing the innovation;	Evaluation and monitoring		(Practical guidance	no	no	no	yes	yes	no	no
695	S34	Scaling up health service delivery: from pi	Simmons, Ruth	Strategic choice: monitoring and evaluation: indi	Examples: number of people with access to quality services over time has increased;	Evaluation and monitoring		(Practical guidance	no	no	no	yes	yes	no	no
696	S34	Scaling up health service delivery: from pi	Simmons, Ruth	Strategic choice: monitoring and evaluation: serv	na	Evaluation and monitoring		(Practical guidance	no	no	no	yes	no	no	no

697	S34	Scaling up health service delivery: from pi	Simmons, Ruth	Strategic choice: monitoring and evaluation: spec	na	Evaluation and monitoring		(Practical guidance	no	no	no	yes	no	no	no
698	S34	Scaling up health service delivery: from pi	Simmons, Ruth	Strategic choice: monitoring and evaluation: loca	na	Evaluation and monitoring		(Practical guidance	no	no	no	yes	no	no	no
699	S34	Scaling up health service delivery: from pi	Simmons, Ruth	Strategic choice: monitoring and evaluation: envi	na	Evaluation and monitoring		(Practical guidance	no	no	no	yes	no	no	no
700	S35	An approach to rapid scale up Using HIV/A	WHO	Establish the country's leadership and coordinating	na	preparing for scaling up	LIST	Box 6	no	no	no	yes	no	no	no
701	S35	An approach to rapid scale up Using HIV/A	WHO	Identify the [AIDS care] expert group	na	preparing for scaling up	LIST	Box 6	no	no	no	yes	no	no	no
702	S35	An approach to rapid scale up Using HIV/A	WHO	Understand the full scale intended	na	Development of scaling strate	LIST	Box 6	no	no	no	yes	no	no	no
703	S35	An approach to rapid scale up Using HIV/A	WHO	Organize the fi rst phase in the multiplicative app	na	Implementation of the strate	LIST	Box 6	no	no	no	no	no	no	no
704	S35	An approach to rapid scale up Using HIV/A	WHO	Organize the fi rst phase in the multiplicative app	na	Implementation of the strate	LIST	Box 6	no	no	no	yes	no	no	no
705	S35	An approach to rapid scale up Using HIV/A	WHO	Organize the fi rst phase in the multiplicative app	na	Implementation of the strate	LIST	Box 6	no	no	no	yes	no	no	no
706	S35	An approach to rapid scale up Using HIV/A	WHO	Organize the fi rst phase in the multiplicative app	na	Implementation of the strate	LIST	Box 6	no	no	no	no	no	no	no
707	S35	An approach to rapid scale up Using HIV/A	WHO	Organize the fi rst phase in the multiplicative app	na	Implementation of the strate	LIST	Box 6	no	no	no	yes	no	no	no
708	S35	An approach to rapid scale up Using HIV/A	WHO	Organize the fi rst phase in the multiplicative app	na	Implementation of the strate	LIST	Box 6	no	no	no	yes	no	no	no
709	S35	An approach to rapid scale up Using HIV/A	WHO	Organize the second phase in the multiplicative a	na	Implementation of the strate	LIST	Box 6	no	no	no	yes	no	no	no
710	S35	An approach to rapid scale up Using HIV/A	WHO	Organize the second phase in the multiplicative a	na	Implementation of the strate	LIST	Box 6	no	no	no	yes	no	no	no
711	S35	An approach to rapid scale up Using HIV/A	WHO	Organize the second phase in the multiplicative a	na	Implementation of the strate	LIST	Box 6	no	no	no	yes	no	no	no
712	S35	An approach to rapid scale up Using HIV/A	WHO	Organize the second phase in the multiplicative a	na	Implementation of the strate	LIST	Box 6	no	no	no	no	no	no	no
713	S35	An approach to rapid scale up Using HIV/A	WHO	Organize the second phase in the multiplicative a	na	Implementation of the strate	LIST	Box 6	no	no	no	yes	no	no	no
714	S35	An approach to rapid scale up Using HIV/A	WHO	Organize additional phases of scale-up	na	Implementation of the strate	LIST	Box 6	no	no	no	yes	no	no	no
715	S35	An approach to rapid scale up Using HIV/A	WHO	List and understand the different factors involvec	na	Evaluation and monitoring	LIST	Box 6	no	no	no	yes	no	no	no
716	S35	An approach to rapid scale up Using HIV/A	WHO	Be aware of the factors that constrain scale-up	na	Evaluation and monitoring	LIST	Box 6	no	no	no	yes	no	no	no
717	S36	Scaling Up Global Health Interventions: A	Yamey, Gavin	Attributes of the Tool or Service Being Scaled Up	Simplicity; Scientifically robust tech	Development of scaling strate	LIST	Page 2	no	no	no	no	no	no	no
718	S36	Scaling Up Global Health Interventions: A	Yamey, Gavin	Attributes of the Implementers:	Strong leadership and governance;	Development of scaling strate	LIST	Page 2	no	no	no	yes	no	no	no
719	S36	Scaling Up Global Health Interventions: A	Yamey, Gavin	The Chosen Delivery Strategy:	Applying diffusion and social netwo	Development of scaling strate	LIST	Page 2	no	no	no	yes	no	no	no
720	S36	Scaling Up Global Health Interventions: A	Yamey, Gavin	Attributes of the ''Adopting'' Community	An engaged, ''activated'' communi	Development of scaling strate	LIST	Page 2	no	no	no	no	no	no	no
721	S36	Scaling Up Global Health Interventions: A	Yamey, Gavin	Socio-Political Context	Political will and national policies; (	Development of scaling strate	LIST	Page 2	no	no	no	yes	no	no	no
722	S36	Scaling Up Global Health Interventions: A	Yamey, Gavin	Research Context	Incorporating research into implem	Development of scaling strate	LIST	Page 2	no	no	no	yes	no	no	no
723	S37	Scaling Up—From Vision to Large-Scale Ch	Larry, Cooley	Develop a Scaling Up Plan : Create a Vision: the M	What Is Being Scaled Up?	Development of scaling strate	LIST	page1	no	no	no	yes	no	no	no
724	S37	Scaling Up—From Vision to Large-Scale Ch	Larry, Cooley	Develop a Scaling Up Plan : Create a Vision: the M	How Will Scaling Up Be Accomplish	Development of scaling strate	LIST	page1	no	no	no	yes	no	no	no
725	S37	Scaling Up—From Vision to Large-Scale Ch	Larry, Cooley	Develop a Scaling Up Plan : Create a Vision: Orga	Who Performs the Key Functions?	Development of scaling strate	LIST	page1	no	no	no	yes	no	no	no
726	S37	Scaling Up—From Vision to Large-Scale Ch	Larry, Cooley	Develop a Scaling Up Plan : Create a Vision: Dime	Where and For Whom Does Scaling	Development of scaling strate	LIST	page1	no	no	no	yes	no	no	no
727	S37	Scaling Up—From Vision to Large-Scale Ch	Larry, Cooley	Develop a Scaling Up Plan : Assess Scalability: det	na	Development of scaling strate	LIST	page1	no	no	no	yes	no	no	no
728	S37	Scaling Up—From Vision to Large-Scale Ch	Larry, Cooley	Develop a Scaling Up Plan : Assess Scalability:Ana	na	Development of scaling strate	LIST	page1	no	no	no	yes	no	no	no
729	S37	Scaling Up—From Vision to Large-Scale Ch	Larry, Cooley	Develop a Scaling Up Plan : Fill Information Gaps	Documentation of the model, Analy	Development of scaling strate	LIST	page1	no	no	yes	yes	no	no	no
730	S37	Scaling Up—From Vision to Large-Scale Ch	Larry, Cooley	Develop a Scaling Up Plan : Prepare a Scaling Up	na	Development of scaling strate	LIST	page1	no	no	no	yes	no	no	no
731	S37	Scaling Up—From Vision to Large-Scale Ch	Larry, Cooley	Establish the Pre-Conditions for Scaling Up: Legit	na	preparing for scaling up	LIST	page1	no	no	yes	yes	no	no	no
732	S37	Scaling Up—From Vision to Large-Scale Ch	Larry, Cooley	Establish the Pre-Conditions for Scaling Up: Build	na	preparing for scaling up	LIST	page1	no	no	yes	yes	no	no	no
733	S37	Scaling Up—From Vision to Large-Scale Ch	Larry, Cooley	Establish the Pre-Conditions for Scaling Up: Realig	na	preparing for scaling up	LIST	page1	no	no	yes	yes	no	no	no
734	S37	Scaling Up—From Vision to Large-Scale Ch	Larry, Cooley	Implement the Scaling Up Process: Modify Orgar	na	Implementation of the strate	LIST	page1	no	no	no	yes	no	no	no
735	S37	Scaling Up—From Vision to Large-Scale Ch	Larry, Cooley	Implement the Scaling Up Process: Coordinate Ac	na	Implementation of the strate	LIST	page1	no	no	no	yes	no	no	no
736	S37	Scaling Up—From Vision to Large-Scale Ch	Larry, Cooley	Implement the Scaling Up Process : Adapt Strateg	na	Implementation of the strate	LIST	page1	no	no	no	yes	no	no	no
737	S38	A model for scale up of family health inno	Elizabeth H, Bradley;	Assess: Understand user group receptivity and er	na	Assessment of scalability of th	list	Figure 3: Schematic	no	no	no	yes	no	no	no
738	S38	A model for scale up of family health inno	Elizabeth H, Bradley;	Innovate: Design and package innovation to fit w	na	Development of scaling strate	list	Figure 3: Schematic	no	no	no	yes	no	no	no
739	S38	A model for scale up of family health inno	Elizabeth H, Bradley;	Develop: Build support and address resistance in	na	preparing for scaling up	list	Figure 3: Schematic	no	no	no	yes	no	no	no
740	S38	A model for scale up of family health inno	Elizabeth H, Bradley;	Engage: introduce, translate, and integrate the in	na	Implementation of the strate	list	Figure 3: Schematic	no	no	no	yes	no	no	no
741	S38	A model for scale up of family health inno	Elizabeth H, Bradley;	Devolve: User group release and spread the innov	na	Implementation of the strate	list	Figure 3: Schematic	no	no	no	yes	no	no	no
742	s39	Scale up of services for mental health in lc	Julian, Eaton	Situation analysis: Gather information about need	Engage with local stakeholders including patients and caregivers30,45,59 to understand their needs and	preparing for scaling up	list	figure3	no	no	yes	yes	no	no	no
743	s39	Scale up of services for mental health in lc	Julian, Eaton	Situation analysis: Identify available resources:• H	Identify available human resources at different levels of existing health services	preparing for scaling up	list	figure3	no	no	no	yes	no	no	no
744	s39	Scale up of services for mental health in lc	Julian, Eaton	Planning: Define priority conditions for service pro	Develop consensus among key stakeholders about the priority conditions requiring services9; Review evidence for	Development of scaling strate	list	figure3	no	no	yes	yes	no	no	no
745	s39	Scale up of services for mental health in lc	Julian, Eaton	Planning: Design a method of service delivery tha	Develop a strong planning and implementation group with effective representation from all stakeholders and external experts as appropriate	Development of scaling strate	list	figure3	no	no	no	yes	no	no	no
746	s39	Scale up of services for mental health in lc	Julian, Eaton	Planning: Identify the barriers to scaling up and d	Identify a mental health focal person at national and district levels59 with responsibility and mandate for seeing changes through	preparing for scaling up	list	figure3	no	no	no	yes	no	no	no
747	s39	Scale up of services for mental health in lc	Julian, Eaton	Implementation: Build coalition of stakeholders t	Strengthen management structures at national and district levels to implement and oversee scaling up41 Include advocacy for sustainable resources in their remit	Implementation of the strate	list	figure3	no	no	no	yes	no	no	no
748	s39	Scale up of services for mental health in lc	Julian, Eaton	Implementation: Implement scaling up strategy a	Raise community awareness about mental health, identification, promotion, prevention, human rights/stigma, how to access services; include patients in this process	Implementation of the strate	list	figure3	no	no	no	yes	no	no	no
749	s39	Scale up of services for mental health in lc	Julian, Eaton	Evaluation:Monitor/evaluate the intervention Mc	Integrate mental health into existing health information systems	Evaluation and monitoring	list	figure3	no	no	no	yes	no	no	no
750	s39	Scale up of services for mental health in lc	Julian, Eaton	Evaluation:Disseminate findings in relevant forun	Develop partnership with academic institutions for formal evaluation and research	Evaluation and monitoring	list	figure3	no	no	no	yes	yes	no	no
